# Clinical Efficacy and Safety of Surgical Treatments in Patients With Pure Cervical Radiculopathy

**DOI:** 10.3389/fpubh.2022.892042

**Published:** 2022-07-14

**Authors:** Quan-You Gao, Fei-Long Wei, Kai-Long Zhu, Cheng-Pei Zhou, Hu Zhang, Wen-Xing Cui, Tian Li, Ji-Xian Qian, Ding-Jun Hao

**Affiliations:** ^1^Health Science Center of Xi'an Jiaotong University, Xi'an, China; ^2^Department of Orthopedics, Tangdu Hospital, Fourth Military Medical University, Xi'an, China; ^3^Department of Spine Surgery, Honghui Hospital, Xi'an Jiao Tong University, Xi'an, China; ^4^School of Basic Medicine, Fourth Military Medical University, Xi'an, China; ^5^Department of Neurosurgery, Tangdu Hospital, Fourth Military Medical University, Xi'an, China

**Keywords:** cervical radiculopathy, surgical treatments, spine surgery, efficacy, safety

## Abstract

**Background:**

Traditionally paired meta-analysis revealed inconsistencies in the safety and effectiveness of surgical interventions. We conducted a network meta-analysis to assess various treatments' clinical efficacy and safety for pure cervical radiculopathy.

**Methods:**

The Embase, PubMed, and Cochrane Library databases were searched for randomized controlled trials (RCTs) comparing different treatment options for patients with pure cervical radiculopathy from inception until October 23, 2021. The primary outcomes were postoperative success rates, postoperative complication rates, and postoperative reoperation rates. The pooled data were subjected to a random-effects consistency model. The protocol was published in PROSPERO (CRD42021284819).

**Results:**

This study included 23 RCTs (*n* = 1,844) that evaluated various treatments for patients with pure cervical radiculopathy. There were no statistical differences between treatments in the consistency model in terms of major clinical effectiveness and safety outcomes. Postoperative success rates were higher for anterior cervical foraminotomy (ACF: probability 38%), posterior cervical foraminotomy (PCF: 24%), and anterior cervical discectomy with fusion and additional plating (ACDFP: 21%). Postoperative complication rates ranked from high to low as follows: cervical disc replacement (CDR: probability 32%), physiotherapy (25%), ACF (25%). Autologous bone graft (ABG) had better relief from arm pain (probability 71%) and neck disability (71%). Among the seven surgical interventions with a statistical difference, anterior cervical discectomy with allograft bone graft plus plating (ABGP) had the shortest surgery time.

**Conclusions:**

According to current results, all surgical interventions can achieve satisfactory results, and there are no statistically significant differences. As a result, based on their strengths and patient-related factors, surgeons can exercise discretion in determining the appropriate surgical intervention for pure cervical radiculopathy.

**Systematic Review Registration:** CRD42021284819.

## Introduction

Cervical radiculopathy is an aging-related disease that typically manifests as neck and shoulder pain (1). The age-adjusted incidence of cervical radiculopathy is 83 per 100,000 people, with men having a slightly higher incidence than women (2). Cervical radiculopathy could be attributed to cervical disks degeneration, cervical disc herniation, osteophytosis of the vertebral bodies, hypertrophy of the facets and laminal arches, ligamentous and segmental instability, and other factors that cause nerve root compression. The most common causes of cervical radiculopathy are degenerative changes in the intervertebral disks and osteophytosis of the vertebral bodies (3, 4). Cervical radiculopathy has a significant impact on the quality of life of the elderly.

Conservative treatment is the first option to treat myelopathy or severe muscle weakness (5). Conservative treatments commonly used include immobilization, anti-inflammatory drugs, physical therapy, and cervical traction. Cervical radiculopathy is a self-limiting disease. Non-surgical treatments relieve symptoms of cervical radiculopathy in more than half of patients (5, 6). Surgical treatments are recommended for patients not responding to conservative treatment (1).

Some meta-analyses compared the effectiveness of surgical treatments for cervical radiculopathy. However, many studies failed to differentiate between patients with myelopathy and those with nerve root symptoms, resulting in unreliable research findings (7–11). Three recent systematic reviews assessed the surgical management of cervical radiculopathy (12–14). Two of these studies only compared two or three types of surgeries (12, 13). Another study performed a paired meta-analysis and did not comprehensively assess the surgical methods used (14). No comprehensive comparison has been conducted to determine which surgery is most beneficial for patients. As a result, evidence-based recommendations are critical to guide clinical practice. To address the limitations of traditionally paired meta-analysis, we developed this network meta-analysis, which can collect data from clinical trials of at least two interventions simultaneously by including direct and indirect information and strengthening inferences on relative efficacy. We presented a comprehensive network meta-analysis comparing the safety and effectiveness of various interventions to provide evidence-based guidance for physicians and patients.

## Methods

### Data Sources and Search Strategy

This was a meta-analysis of randomized clinical trials (RCTs) conducted according to the Cochrane Handbook for Systematic Reviews of Interventions and the Preferred Reporting Items for Systematic Reviews and Meta-Analyses (PRISMA) (15) and Assessing the methodological quality of systematic reviews (AMSTAR) guidelines (16). The Embase, PubMed, and Cochrane Library databases were searched with no language limitations from inception to October 23, 2021. The search strategy is described in detail in Supplementary Table 1. Following the preliminary screening of titles and abstracts, two independent reviewers assessed related publications. The protocol of this study was published and registered in PROSPERO (CRD42021284819).

### Selection Criteria and Study Design

The studies were screened according to the PICOS (population, intervention, comparison, outcome, study design) criteria. The selection criteria are detailed in Supplementary Table 2.

### Data Extraction and Outcomes

We extracted data from the included articles, including investigator characteristics, surgical methods, participant characteristics, and main results. Two authors independently worked on this section. The primary outcomes were postoperative success rates, postoperative complication rates, and postoperative reoperation rates. The secondary outcomes included postoperative work status, arm and neck pain scores, the neck disability index (NDI), and surgery time.

### Quality and Risk of Bias Assessment

The Cochrane Collaboration risk-of-bias assessment tool (17) was used by two reviewers to evaluate the included studies for potential bias independently. Disagreements between the two investigators were resolved by bringing in a third investigator. The overall risk of bias is calculated and classified as “high risk,” “low risk,” or “unclear risk.” The tool to assess the risk of bias has been described in detail in Supplementary Table 3.

### Data Synthesis and Statistical Analysis

Firstly, a random-effects model was used for pairwise analysis to pool relative risks (RRs) or mean difference (MD) and 95% confidence intervals (CIs) (18). *P* < 0.05 was considered significant. Forest plots and *I*^2^ were used to explore sources of heterogeneity (19). Secondly, the network geometry was generated using Stata version 16.0 (Stata Corp). Then a Bayesian network meta-analysis was performed using Markov chain Monte Carlo methods in WinBUGS version 1.4.3 (MRC Biostatistics Unit, Cambridge, United Kingdom) (20) using a random-effects consistency model (21). Each surgical intervention's safest and most effective probability was ranked first, followed by second, third, etc., based on the average difference and the risk ratio. As the stability of the results is crucial for network meta-analysis, we used various methods to assess the inconsistency of the results.

The consistency and inconsistency models are compared, and the inconsistency is initially estimated roughly. The entire network on detailed comparisons (nodes) was tested by node splitting analysis; *P* < 0.05 manifested a significant inconsistency. The indirect results (network meta-analysis results) were then compared with the pairwise direct results (meta-analysis results) to determine the source of the inconsistency. The intervertebral spacer was used to conduct a sensitivity analysis of anterior cervical discectomy and fusion (ACDF) (Zero-P and the other).

## Results

### A Systematic Review and Qualitative Assessment

**Figure 1:**
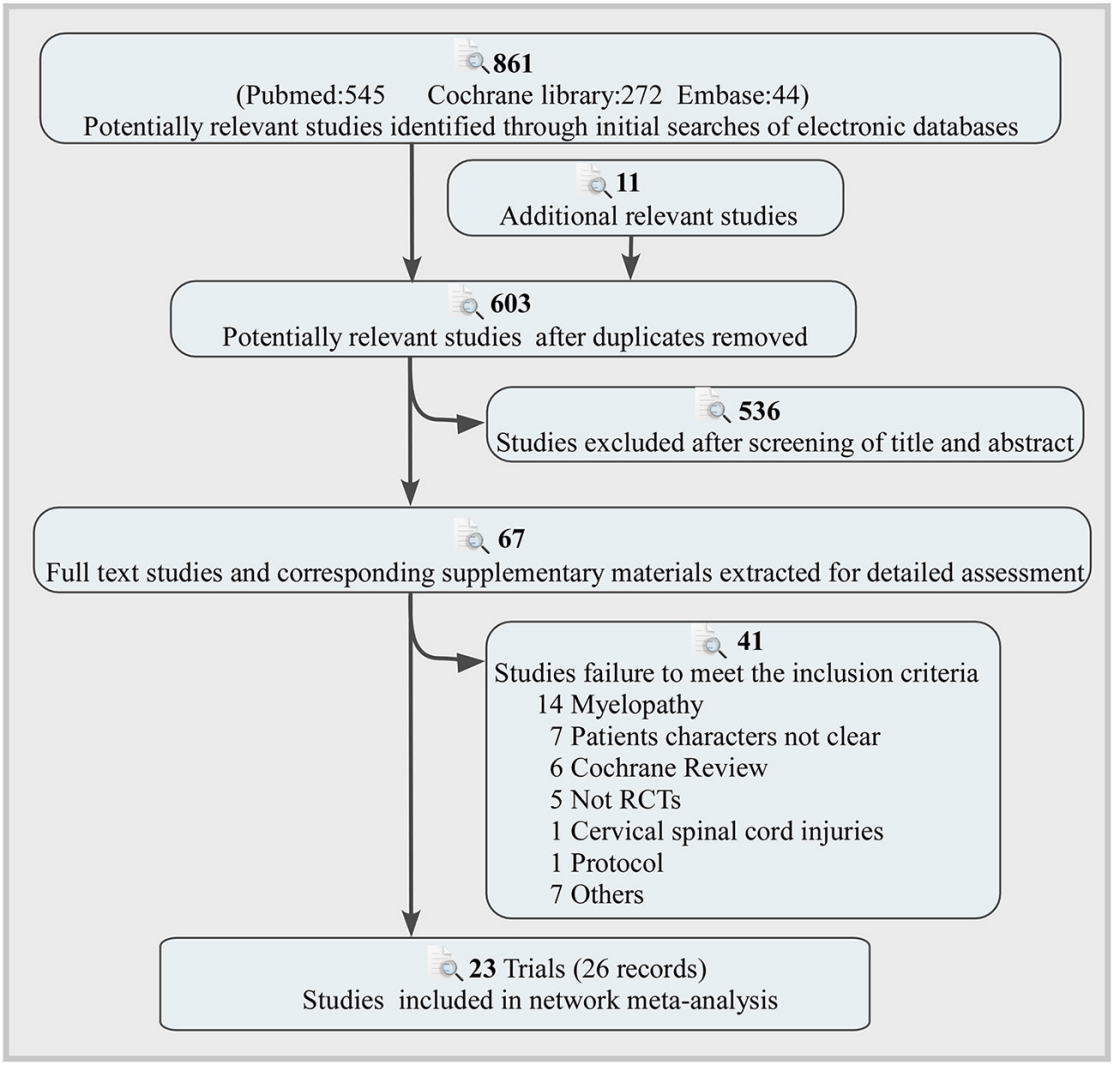
Literature search and screening process.

The flow of the selection process and the reasons for exclusion are depicted in Figure 1. These electronic searches yielded 861 potentially relevant studies, of which 67 potentially relevant articles were thoroughly evaluated. Finally, 23 trials (26 records) including 1,844 participants were included in the final analysis (22–44). Ten interventions were performed that had anterior cervical discectomy with autologous bone graft (ABG), anterior cervical discectomy with allograft bone graft plus plating (ABGP), anterior cervical discectomy (ACD), ACDF, anterior cervical discectomy with fusion and additional plating (ACDFP), anterior cervical foraminotomy (ACF), cervical disc replacement (CDR), posterior cervical foraminotomy (PCF), anterior cervical discectomy with polymethylmethacrylate (PMMA) and physiotherapy. Most of these studies (69.6%) were conducted in Europe. The characteristics of the included trials and participants are shown in Supplementary Table 4. Two studies showed high risk for generating the randomization sequence (30, 34). Two studies showed high risk in concealing allocation (30, 34). Ten studies showed high risk in blinding of participants and personnel (26, 29, 30, 32, 34–36, 41). Two studies showed high risk in blinding of outcome assessment (37, 40). One study showed high risk in incomplete outcome data (32). Four studies showed high risk in selective outcome reporting (26, 27, 29, 37). Supplementary Figures 1, 2 summarize the risk of bias assessment.

### Primary Outcomes

#### Postoperative Success Rates

**Figure 2:**
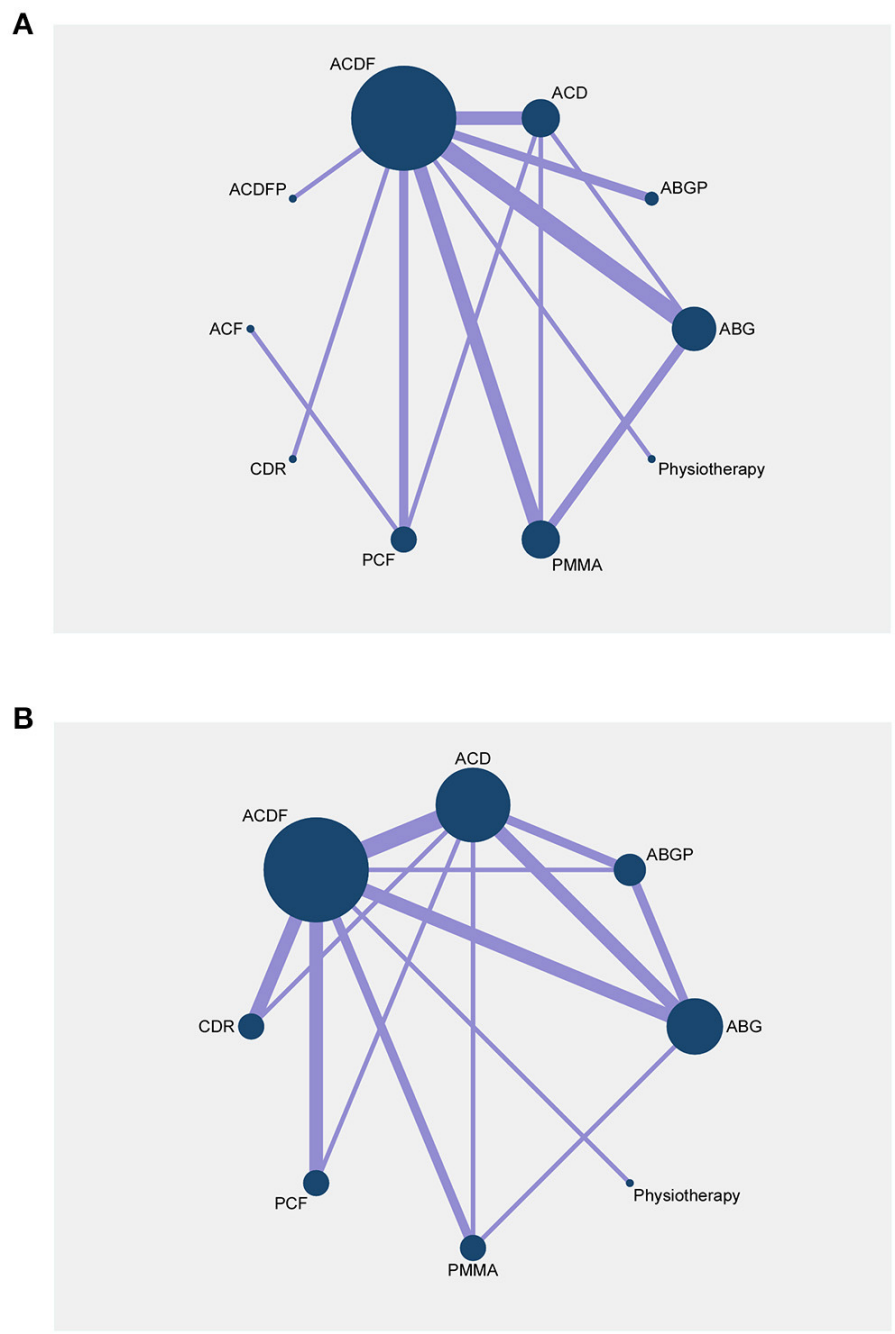
Network plots of comparisons for network meta-analyses based on postoperative success rates **(A)** and postoperative complication rates **(B)**. Each circular node represents a type of treatment. The size of the circle is proportional to the total number of patients. The width of the lines is proportional to the number of studies performing head-to-head comparisons in the same study. ABG, anterior cervical discectomy with autologous bone graft; ABGP, anterior cervical discectomy with allograft bone graft plus plating; ACD, anterior cervical discectomy; ACDF, anterior cervical discectomy and fusion; ACDFP, anterior cervical discectomy with fusion and additional plating; ACF, anterior cervical foraminotomy; CDR, cervical disc replacement; PCF, posterior cervical foraminotomy; PMMA, anterior cervical discectomy with polymethylmethacrylate.

**Figure 3:**
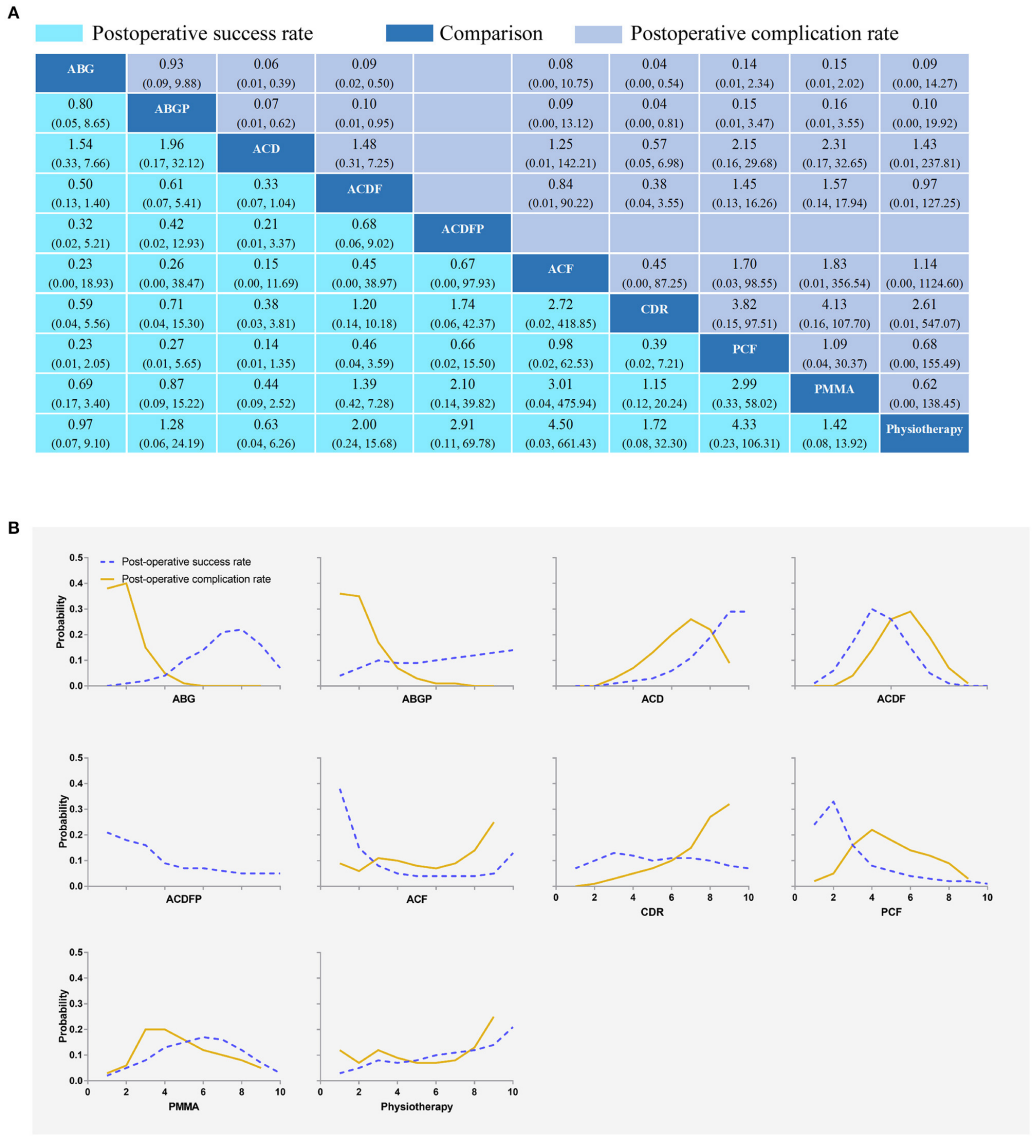
Network plots of comparisons **(A)** and rank probability **(B)** for post-operative success rates and postoperative complication rates based on network meta-analyses. Each cell profile **(A)** contains the pooled RR and 95% CI; significant results are bold. The ranking curves **(B)** indicate the probability of the highest postoperative success rates and postoperative complication rates, the second-lowest, the third-lowest, etc. ABG, anterior cervical discectomy with autologous bone graft; ABGP, anterior cervical discectomy with allograft bone graft plus plating; ACD, anterior cervical discectomy; ACDF, anterior cervical discectomy and fusion; ACDFP, anterior cervical discectomy with fusion and additional plating; ACF, anterior cervical foraminotomy; CDR, cervical disc replacement; PCF, posterior cervical foraminotomy; PMMA, anterior cervical discectomy with polymethylmethacrylate.

**Figure 4:**
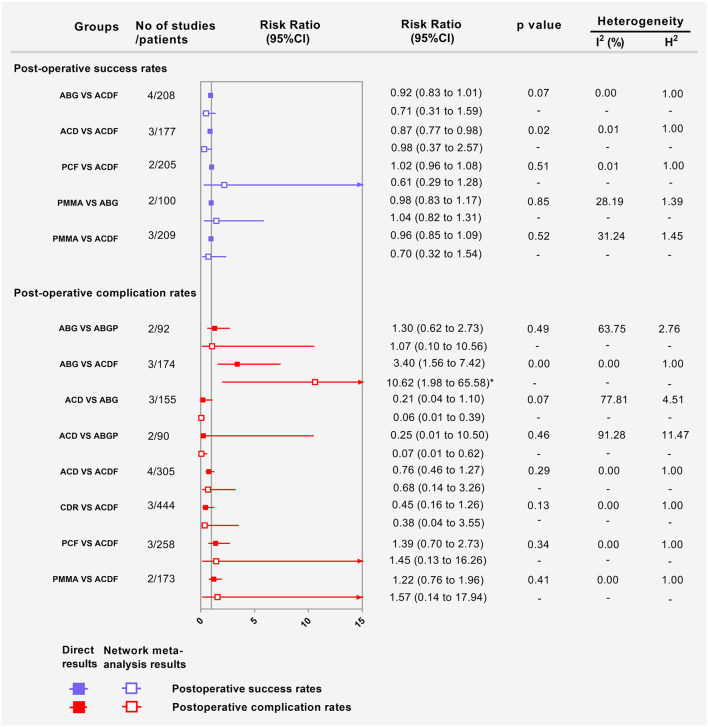
Forest plots showing the direct and indirect results of postoperative success rates and postoperative complication rates of head-to-head comparisons. ABG, anterior cervical discectomy with autologous bone graft; ABGP, anterior cervical discectomy with allograft bone graft plus plating; ACD, anterior cervical discectomy; ACDF, anterior cervical discectomy and fusion; CDR, cervical disc replacement; PCF, posterior cervical foraminotomy; PMMA, anterior cervical discectomy with polymethylmethacrylate. ^*^Values in brackets are 95% CI.

Fourteen RCTs with 1,053 participants compared the differences in postoperative success rates under various interventions (Figure 2A) (22, 24, 26, 28, 29, 31–35, 37–39, 42, 43). There were no statistical differences in the postoperative success rates of various interventions, including physical therapy (Figure 3A). The results from consistency model fit well-with the results from inconsistency model; node splitting analyses revealed no significant inconsistency (all *P* > 0.05; Supplementary Table 5). Direct results were detailed in Supplementary Figures 3–7. Figure 4 shows the direct and indirect results of comparing different interventions. The direct results were identical to the corresponding indirect results regarding significance and tendency. Figure 3B depicted the probability distribution of postoperative success rates for each intervention arranged in ten possible positions. Postoperative success rates ranking from high to low were as follows: ACF (probability 38%), PCF (24%), ACDFP (21%), CDR (7%), ABGP (4%), physiotherapy (3%), PMMA (2%), ACDF (1%), ACD (0%), and ABG (0%). The probabilities are detailed in Supplementary Table 6.

#### Postoperative Complication Rates

Fifteen RCTs with 1,470 participants compared the differences in postoperative complication rates under different interventions (Figure 2B) (22, 24, 26, 28, 29, 31–35, 37–39, 42, 43). There were no statistical differences in postoperative complication rates between interventions, including physiotherapy (Figure 3A). The results obtained using the consistency model fit well-with the inconsistency model; Node splitting analyzes did not show significant inconsistency (all *P* > 0.05; Supplementary Table 7). The direct results were detailed in Supplementary Figures 8–15. Figure 4 shows the direct and indirect results of comparing different interventions. The direct results were prominently consistent with the corresponding indirect results insignificance and tendency. Figure 3B showed the distribution of postoperative complication rates probability for each intervention arranged in nine possible positions. Postoperative complication rates ranging from high to low was as follows: CDR (probability 32%), physiotherapy (25%), ACF (25%), ACD (9%), PMMA (5%), PCF (3%), ACDF (1%), ABG (0%), and ABGP (0%). The probabilities are detailed in Supplementary Table 8.

#### Postoperative Reoperation Rates

**Figure 5:**
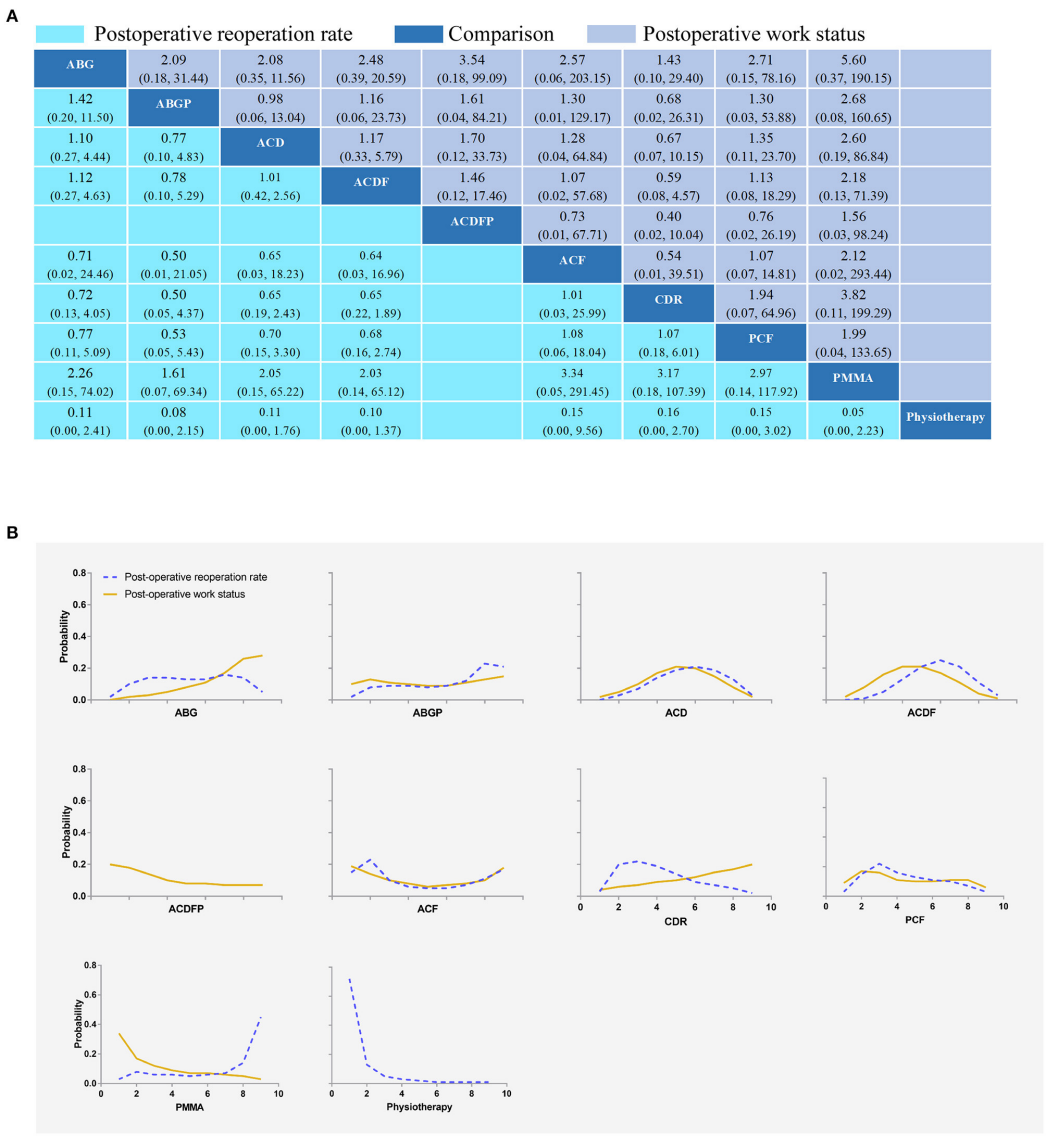
Network plots of comparisons **(A)** and rank probability **(B)** for postoperative reoperation rates and postoperative work status-based network meta-analyses. Each cell profile **(A)** contains the pooled RR and 95% CI; significant results are bold. Ranking curves **(B)** indicate the probability of the highest postoperative reoperation rates and postoperative work status, the second-lowest, the third-lowest, etc. ABG, anterior cervical discectomy with autologous bone graft; ABGP, anterior cervical discectomy with allograft bone graft plus plating; ACD, anterior cervical discectomy; ACDF, anterior cervical discectomy and fusion; ACDFP, anterior cervical discectomy with fusion and additional plating; ACF, anterior cervical foraminotomy; CDR, cervical disc replacement; PCF, posterior cervical foraminotomy; PMMA, anterior cervical discectomy with polymethylmethacrylate.

**Figure 6:**
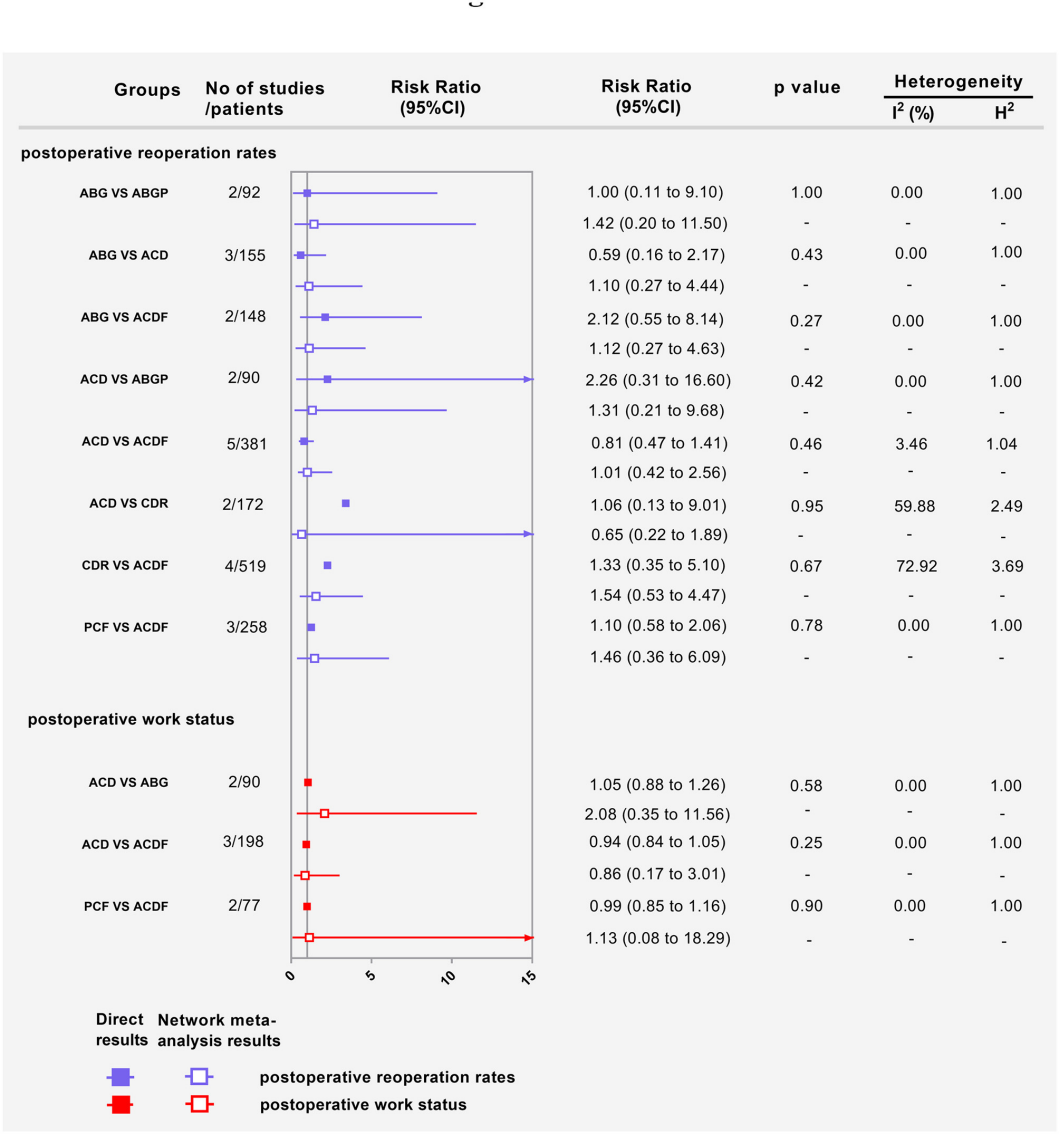
Forest plots showing the direct and indirect results of post-operative reoperation rates and postoperative work status of head-to-head comparisons. ABG, anterior cervical discectomy with autologous bone graft; ABGP, anterior cervical discectomy with allograft bone graft plus plating; ACD, anterior cervical discectomy; ACDF, anterior cervical discectomy and fusion; CDR, cervical disc replacement; PCF, posterior cervical foraminotomy.

Fourteen RCTs with 1,449 participants compared the differences in postoperative complication rates under various interventions (Supplementary Figure 16) (24, 26, 28, 32–35, 37–39, 42–44). No statistical differences were found in postoperative reoperation rates of different interventions, including physical therapy (Figure 5A). The results obtained using the consistency model fit well-with the inconsistency model; Node splitting analyzes did not show significant inconsistency (all *P* > 0.05; Supplementary Table 9). Direct results were detailed in Supplementary Figures 17–24. Figure 6 shows the direct and indirect results of comparing different interventions. The direct results were prominently consistent with the corresponding indirect results insignificance and tendency. Figure 5B depicted the probability distribution of postoperative reoperation rates for each intervention arranged in nine possible positions. Postoperative reoperation rates ranging from high to low were as follows: CDR (probability 32%), physiotherapy (25%), ACF (25%), ACD (9%), PMMA (5%), PCF (3%), ACDF (1%), ABG (0%) and ABGP (0%). The probabilities are detailed in Supplementary Table 10.

### Secondary Outcomes

#### Postoperative Work Status

Eight RCTs with 493 participants compared differences in postoperative work status under different interventions (Supplementary Figure 25) (23, 24, 26, 28, 33, 37, 43). There were no statistical differences in postoperative work status between interventions, including physical therapy (Figure 5A). The results obtained using the consistency model fit well-with the inconsistency model; Node splitting analyzes did not show significant inconsistency (all *P* > 0.05; Supplementary Table 11). Direct results were detailed in Supplementary Figures 26–28. Figure 6 shows the direct and indirect results of comparing different interventions. The direct results were prominently consistent with the corresponding indirect results insignificance and tendency. Figure 5B depicted the postoperative work status probability distribution for each intervention, which was arranged into nine possible positions. Postoperative work status ranking from high to low was as follows: PMMA (probability 34%), ACDFP (20%), ACF (19%), ABGP (10%), PCF (9%), CDR (4%), ACD (2%), ACDF (2%), and ABG (0%). The probabilities are detailed in Supplementary Table 12.

### Scores for Arm and Neck Pain

**Figure 7:**
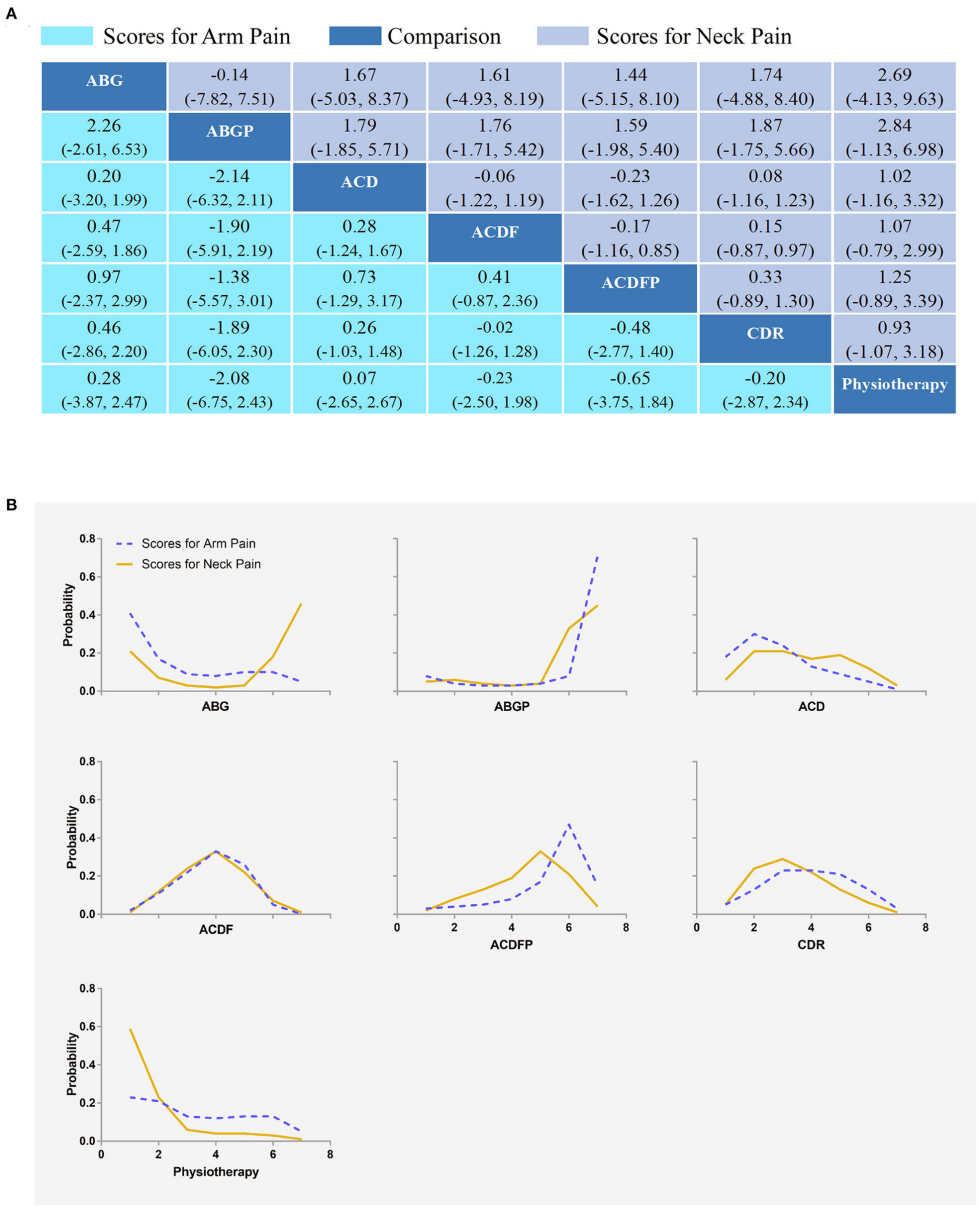
Network plots of comparisons **(A)** and rank probability **(B)** for arm and neck pain scores based on network meta-analyses. Each cell profile **(A)** contains the pooled RR and 95% CI; significant results are bold. The ranking curves **(B)** indicate the probability of the highest rate of scores for arm and neck pain, the second-lowest, the third-lowest, etc. ABG, anterior cervical discectomy with autologous bone graft; ABGP, anterior cervical discectomy with allograft bone graft plus plating; ACD, anterior cervical discectomy; ACDF, anterior cervical discectomy and fusion; ACDFP, anterior cervical discectomy with fusion and additional plating; CDR, cervical disc replacement.

Eight RCTs with a sum of 562 participants compared the differences in arm pain scores under different interventions (Supplementary Figure 29) (23, 27, 29, 38, 42–44). Eight RCTs (627 participants) compared the differences in neck pain scores under different interventions (Supplementary Figure 30) (23, 27, 35, 38, 40, 42–44). No statistical differences were found in scores for arm and neck pain of different interventions (Figure 7A). The results obtained using the consistency model fit well-with the inconsistency model; Node splitting analyzes did not show significant inconsistency (all *P* > 0.05; Supplementary Tables 13, 15). Direct results were detailed in Supplementary Figures 31–36. Supplementary Figure 37 shows the direct and indirect results of comparing different interventions. The direct results were prominently consistent with the corresponding indirect results insignificance and tendency. Figure 7B showed the distribution of arm and neck pain probability scores for each intervention arranged in seven possible positions. Scores for arm pain ranging from low to high were as follows: ABGP (probability 71%), ACDFP (15%), ABG (5%), Physiotherapy (5%), CDR (3%), ACD (1%), and ACDF (0%). The probabilities are detailed in Supplementary Table 14. The scores for neck pain ranging from low to high was as follows: ABG (probability 46%), ABGP (45%), ACDFP (4%), ACD (3%), ACDF (1%), CDR (1%), and physiotherapy (1%). The probabilities are detailed in Supplementary Table 16.

### Neck Disability Index (NDI)

**Figure 8:**
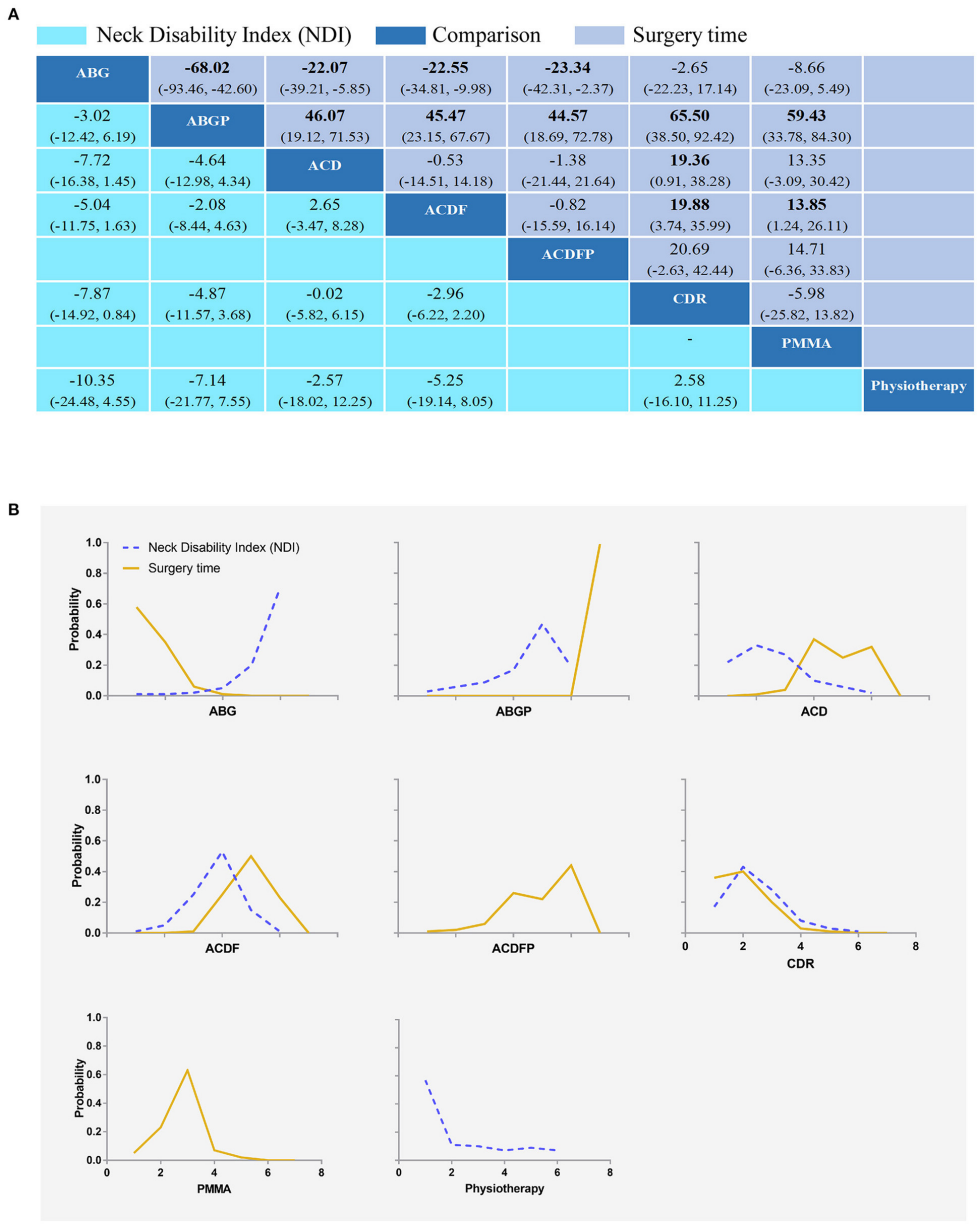
Network plots of comparisons **(A)** and rank probability **(B)** for neck disability index (NDI) and surgery time-based network meta-analyses. Each cell profile **(A)** contains the pooled RR and 95% CI; significant results are bold. The ranking curves **(B)** indicate the probability of the highest rate of scores for arm and neck pain, the second-lowest, the third-lowest, etc. ABG, anterior cervical discectomy with autologous bone graft; ABGP, anterior cervical discectomy with allograft bone graft plus plating; ACD, anterior cervical discectomy; ACDF, anterior cervical discectomy and fusion; ACDFP, anterior cervical discectomy with fusion and additional plating; CDR, cervical disc replacement; PMMA, anterior cervical discectomy with polymethylmethacrylate.

Six RCTs (575 participants) compared the differences in neck disability index under different interventions (Supplementary Figure 38) (32, 35, 38, 42–44). No statistical differences were found in the neck disability index of different interventions (Figure 8A). The results obtained using the consistency model fit well-with the inconsistency model. The direct results were detailed in Supplementary Figures 39–41. Supplementary Figure 42 showed the direct and indirect results of comparing different interventions. The direct results were prominently consistent with the corresponding indirect results insignificance and tendency. Figure 8B showed the distribution of the probability of neck disability index for each intervention organized in six possible positions. The neck disability index ranging from low to high was as follows: ABG (probability 71%), ABGP (19%), physiotherapy (7%), ACD (2%), ACDF (1%), and CDR (1%). The probabilities are detailed in Supplementary Table 17.

### Surgery Time

Nine RCTs with 820 participants compared the differences in surgery time under different interventions (Supplementary Figure 43) (26, 31, 32, 35, 36, 39–41, 44). In terms of surgery time (Figure 8A), ABGP (MD, −68.02; [95% CI, −93.46 to −42.60]), ACD (MD, −22.07; [95% CI, −39.21 to −5.85]), ACDF (MD, −22.55; [95% CI, −34.81 to −9.98]), and ACDFP (MD, −23.34; [95% CI, −42.31 to −2.37]) were with shorter surgery time compared with ABG in the consistency model. ACD (MD, 46.07; [95% CI, 19.12 to 71.53]), ACDF (MD, 45.47; [95% CI, 23.15 to 67.67]), ACDFP (MD, 44.57; [95% CI, 18.69 to 72.78]), CDR (MD, 65.50; [95% CI, 38.50 to 92.42]), and PMMA (MD, 59.43; [95% CI, 33.78 to 84.30]) had a longer surgery time compared with ABGP in the consistency model. ACD (MD, −19.36; [95% CI, −38.28 to −0.91]) and ACDF (MD, −19.88; [95% CI, −35.99 to −3.74]) were with shorter surgery time compared to CDR in the consistency model. Furthermore, ACDF (MD, −13.85; [95% CI, −26.11 to −1.24]) were with shorter surgery time compared with PMMA in the consistency model. The results obtained using the consistency model fit well-with the results using the inconsistency model; Node splitting analyzes did not show significant inconsistency (all *P* > 0.05; Supplementary Table 18). Direct results were detailed in Supplementary Figures 44–49. Supplementary Figure 42 showed the direct and indirect results of the comparison of different interventions. The direct results were prominently consistent with the corresponding indirect results in significance and tendency. Figure 8B showed the distribution of the probability of surgery time for each intervention organized into seven possible positions. Among the seven surgical interventions, ABGP had the shortest surgery time. The probabilities are detailed in the Supplementary Table 19.

## Discussion

One of the most common reasons for spinal surgery is cervical radiculopathy. However, the evidence on the most effective surgical technique is conflicting. As a result, we conducted a comprehensive network meta-analysis to compare the safety and efficacy of various interventions. This network meta-analysis included 23 RCTs involving 1,844 cervical radiculopathy without myelopathy treated with ten different types of interventions. In summary, we did not find statistically significant differences in the safety and efficacy of ten various interventions. ABGP achieved the shortest surgery time.

For the treatment of cervical radiculopathy, the anterior approach is the most commonly used surgical option. ACDF, reported in 1958 (45), is a mature and effective treatment that removes all diseased intervertebral disks, including compressed disc material and osteophytes from the anterior spinal cord canal and the nerve root foramen. Segmental fixation after fusion has been established to cause additional biomechanical stress and degeneration of adjacent segments, which usually results in symptoms (46, 47). We found no statistical difference in postoperative complications or reoperation rates between ACDF and other treatments in our study, which is consistent with the findings of most studies (14, 42, 44). The ideal fusion substrate is still debatable. The autologous bone graft is still a popular fusion substrate (36). The ABGP operation time was found to be the shortest in our study, but autogenous bone grafting may cause iliac discomfort.

Total disc replacement, like fusion, aims to remove the entire disc and restore the segment's stability. Total disc replacement, unlike fusion, allows the surgically treated disc to move (48). This continuous movement at the surgical treatment level may protect adjacent moving segments (49). However, the current study did not find that the reoperation rate of CDR is lower than that of ACDF, which is consistent with the findings of the previous study (14).

With the popularity of minimally invasive techniques in recent years, the minimally invasive posterior cervical foraminal incision (MI-PCF) has become a popular alternative treatment option. Based on a solid body of evidence, MI-PCF is a successful alternative surgery to reduce problems such as false joints, adjacent segment diseases, and anterior-related complications. MI-PCF does not necessitate the patient giving up a cervical spine motion segment, and it has a lower complication rate, a lower cost, and a faster return to movement (50, 51). Based on the findings of this study, PCF, similar to other interventions, produced satisfactory results, with no statistical difference in postoperative success rates, post-operative complication rates, or postoperative working status, which is consistent with previous study findings (52).

### Strengths and Limitations

This study is the first network meta-analysis that provides an evidence-based comparative evaluation of all surgical interventions for cervical radiculopathy. We have used an innovative method of comparing indirect results (network meta-analysis results) and pairwise direct results (meta-analysis results) to investigate the source of heterogeneity. Our research does, however, have limitations. The sample sizes in the studies were insufficient, which reduced the reliability of the results. The prognostic indicators were reported at various time points, resulting in heterogeneity. Furthermore, the surgical level of different surgeons may contribute to heterogeneity. In addition, although all RCTs included patients with pure cervical radiculopathy, most of the included studies did not report the localization of the degenerative disease (e.g., central, paracentral, foraminal). This is an important factor in the surgeon's decision-making process, as some surgical techniques have specific contraindications.

## Conclusions

The best surgical treatment for cervical radiculopathy has been a source of controversy. Numerous factors influence the choice of surgery, in addition to clinical outcomes and surgical safety. All surgical interventions, in general, can produce satisfactory results, and there is no statistical difference. Consequently, surgeons can select the appropriate surgical interventions based on their strengths and the particular characteristics of patients with pure cervical radiculopathy.

## Data Availability Statement

The original contributions presented in the study are included in the article/Supplementary Material, further inquiries can be directed to the corresponding author/s.

## Author Contributions

Concept and design: DJ-H, J-XQ, Q-YG, F-LW, and TL. Acquisition, analysis, and interpretation of data: F-LW, Q-YG, K-LZ, HZ, W-XC, TL, J-XQ, and D-JH. Drafting of the manuscript: Q-YG, F-LW, and K-LZ. Statistical analysis: F-LW, TL, and Q-YG. Administrative, technical, or material support: J-XQ, D-JH, TL, F-LW, and Q-YG. Supervision: J-XQ, D-JH, Q-YG, and TL. Critical revision of the manuscript for important intellectual content: All authors.

## Funding

This work was supported by grants from the National Natural Science Foundation of China (No. 81871818) and Tangdu Hospital Seed Talent Program (F-LW). The funding body had no role in the design of the study, data collection, analysis, interpretation or in writing the manuscript.

## Conflict of Interest

The authors declare that the research was conducted in the absence of any commercial or financial relationships that could be construed as a potential conflict of interest.

## Publisher's Note

All claims expressed in this article are solely those of the authors and do not necessarily represent those of their affiliated organizations, or those of the publisher, the editors and the reviewers. Any product that may be evaluated in this article, or claim that may be made by its manufacturer, is not guaranteed or endorsed by the publisher.
