# Supplementary material for: Clinical Efficacy and Safety of Surgical Treatments in Patients With Pure Cervical Radiculopathy
Source: Front Public Health. 2022 Jul 14;10:892042. doi: 10.3389/fpubh.2022.892042 (PMC9330161; doi:10.3389/fpubh.2022.892042)
Supplement: Supplementary file 1 [file Data_Sheet_1.docx]

**Supplementary files**

**1. Figures** (Supplementary Figure 1- Supplementary Figure 49)

**Supplementary Figure 1.** Risk of bias summary of included studies.

**Supplementary Figure 2.** Risk of bias of included studies.

**Supplementary Figure 3.** ABG compared with ACDF on postoperative success rates.

**Supplementary Figure 4.** ACD compared with ACDF on postoperative success rates.

**Supplementary Figure 5.** PCF compared with ACDF on postoperative success rates.

**Supplementary Figure 6.** PMMA compared with ABG on postoperative success rates.

**Supplementary Figure 7.** PMMA compared with ACDF on postoperative success rates.

**Supplementary Figure 8.** ABG compared with ABGP on postoperative complication rates.

**Supplementary Figure 9.** ABG compared with ACDF on postoperative complication rates.

**Supplementary Figure 10.** ACD compared with ABG on postoperative complication rates.

**Supplementary Figure 11.** ACD compared with ABGP on postoperative complication rates.

**Supplementary Figure 12.** ACD compared with ACDF on postoperative complication rates.

**Supplementary Figure 13.** CDR compared with ACDF on postoperative complication rates.

**Supplementary Figure 14.** PCF compared with ACDF on postoperative complication rates.

**Supplementary Figure 15.** PMMA compared with ACDF on postoperative complication rates.

**Supplementary Figure 16.** Network plots of comparison-based network meta-analyses on post-operative reoperation rates.

**Supplementary Figure 17.** ABG compared with ABGP on postoperative reoperation rates.

**Supplementary Figure 18.** ABG compared with ACD on postoperative reoperation rates.

**Supplementary Figure 19.** ABG compared with ACDF on postoperative reoperation rates.

**Supplementary Figure 20.** ACD compared with ABGP on postoperative reoperation rates.

**Supplementary Figure 21.** ACD compared with ACDF on postoperative reoperation rates.

**Supplementary Figure 22.** ACD compared with CDR on postoperative reoperation rates.

**Supplementary Figure 23.** CDR compared with ACDF on postoperative reoperation rates.

**Supplementary Figure 24.** PCF compared with ACDF on postoperative reoperation rates.

**Supplementary Figure 25.** Network plots of comparison-based network meta-analyses on post-operative work status.

**Supplementary Figure 26.** ACD compared with ABG on postoperative work status.

**Supplementary Figure 27.** ACD compared with ACDF on postoperative work status.

**Supplementary Figure 28.** PCF compared with ACDF on postoperative work status.

**Supplementary Figure 29.** Network plots of comparison-based network meta-analyses on scores for arm pain.

**Supplementary Figure 30.** Network plots of comparison-based network meta-analyses on scores for neck pain.

**Supplementary Figure 31.** ACD compared with ACDF on scores for arm pain.

**Supplementary Figure 32.** ACD compared with CDR on scores for arm pain.

**Supplementary Figure 33.** CDR compared with ACDF on scores for arm pain.

**Supplementary Figure 34.** CDR compared with ACDF on scores for neck pain.

**Supplementary Figure 35.** ACD compared with CDR on scores for neck pain.

**Supplementary Figure 36.** CDR compared with ACDF on scores for neck pain.

**Supplementary Figure 37.** Forest plots depicting the direct and indirect results of scores for arm and neck pain of head-to-head comparisons.

**Supplementary Figure 38.** Network plots of comparison-based network meta-analyses on neck disability index (NDI).

**Supplementary Figure 39.** ACD compared with ACDF on neck disability index (NDI).

**Supplementary Figure 40.** ACD compared with CDR on neck disability index (NDI).

**Supplementary Figure 41.** ACDF compared with CDR on neck disability index (NDI).

**Supplementary Figure 42.** Forest plots depicting the direct and indirect results of neck disability index (NDI) and surgery time of head-to-head comparisons.

**Supplementary Figure 43.** Network plots of comparison-based network meta-analyses on surgery time.

**Supplementary Figure 44.** ABG compared with ACDF on surgery time. **Supplementary Figure 45.** ABG compared with PMMA on surgery time.

**Supplementary Figure 46.** ABGP compared with ACDF on surgery time.

**Supplementary Figure 47.** ACD compared with ACDF on surgery time.

**Supplementary Figure 48.** CDR compared with ACDF on surgery time.

**Supplementary Figure 49.** PMMA compared with ACDF on surgery time.

**2. Tables** (Supplementary Table 1- Supplementary Table 19)

**Supplementary Table 1.** Search strategy.

**Supplementary Table 2.** Inclusion/exclusion criteria of literature.

**Supplementary Table 3.** Risk of bias table.

**Supplementary Table 4.** Characteristics of the Included Trials and Participants.

**Supplementary Table 5.** Node splitting analyses on postoperative success rates.

**Supplementary Table 6.** Rank possibility of postoperative success rates.

**Supplementary Table 7.** Node splitting analyses on postoperative complication rates.

**Supplementary Table 8.** Rank possibility of postoperative complication rates.

**Supplementary Table 9.** Node splitting analyses on postoperative reoperation rates.

**Supplementary Table 10.** Rank possibility of postoperative reoperation rates.

**Supplementary Table 11.** Node splitting analyses on postoperative work status.

**Supplementary Table 12.** Rank possibility of postoperative work status.

**Supplementary Table 13.** Node splitting analyses on scores for arm pain.

**Supplementary Table 14.** Rank possibility of scores for arm pain.

**Supplementary Table 15.** Node splitting analyses on scores for neck pain.

**Supplementary Table 16.** Rank possibility of scores for neck pain.

**Supplementary Table 17.** Rank possibility of scores for neck disability index (NDI).

**Supplementary Table 18.** Node splitting analyses on surgery time.

**Supplementary Table 19.** Rank possibility of scores for surgery time.

**Supplementary Figure 1. Risk of bias summary of included studies.**


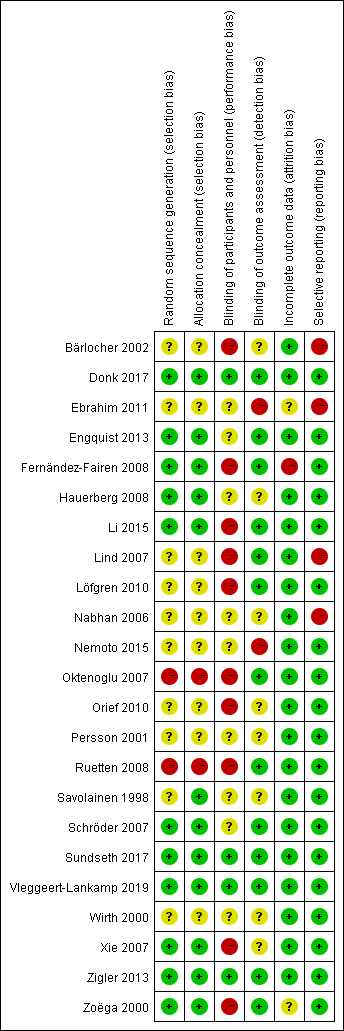


**Supplementary Figure 2.** **Risk of bias of included studies.**


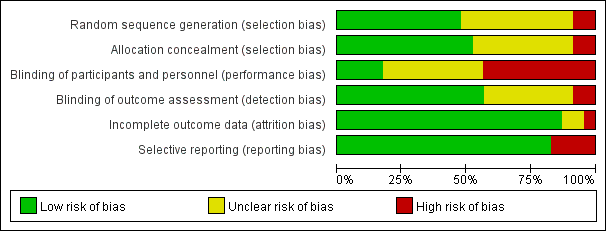


**Supplementary Figure 3.** **ABG compared with ACDF on postoperative success rates.**
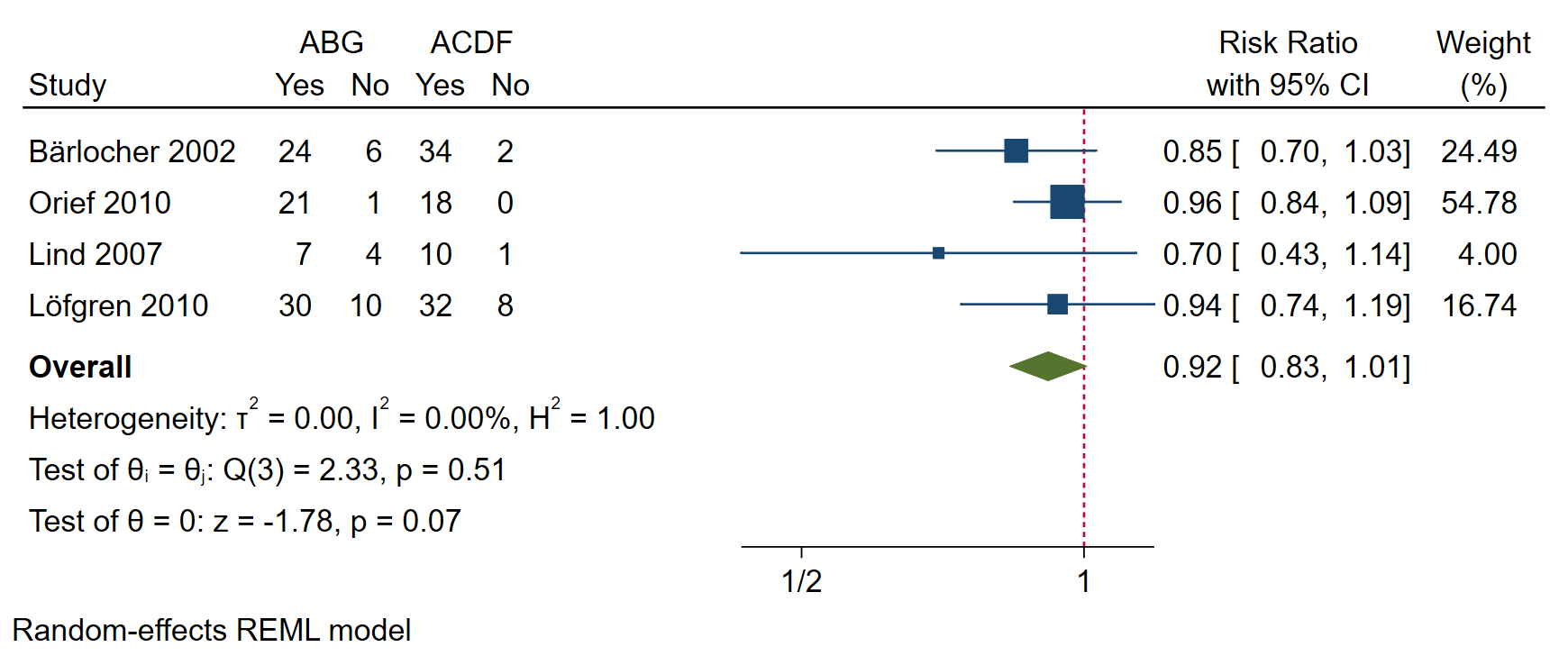


**Supplementary Figure 4.** **ACD compared with ACDF on postoperative success rates.**


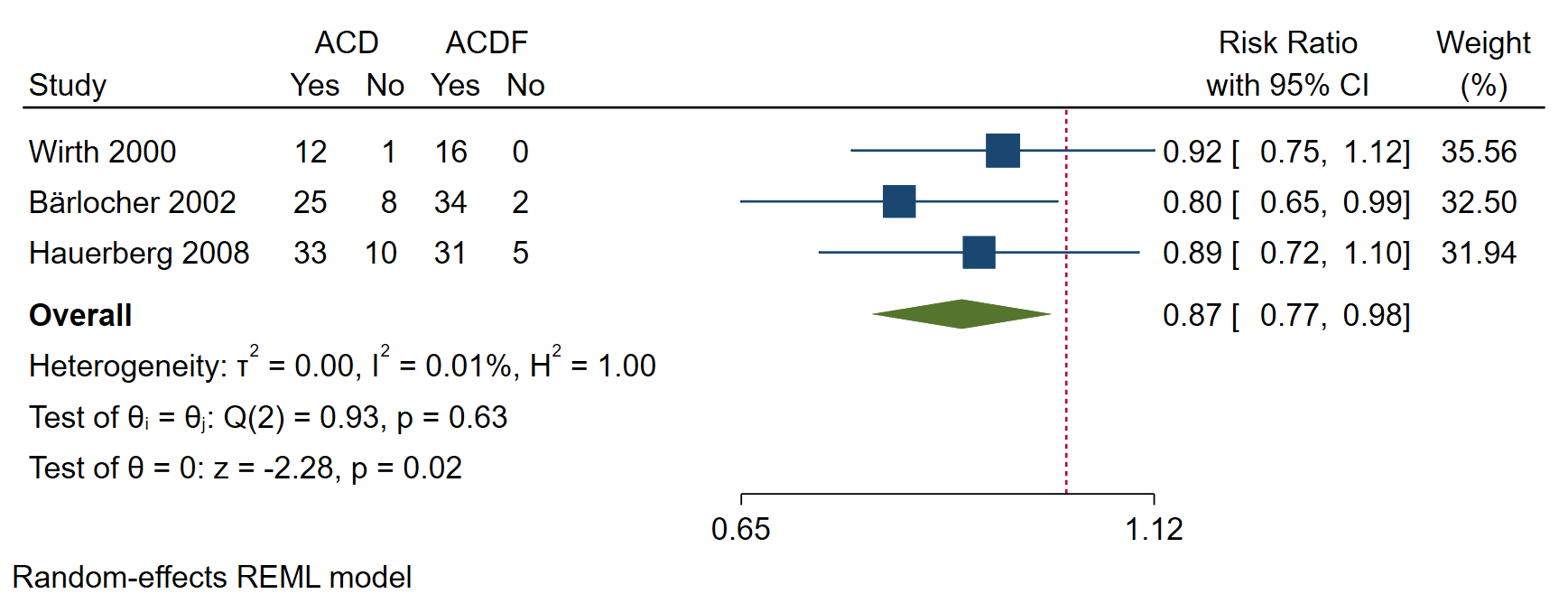


**Supplementary Figure 5.** **PCF compared with ACDF on postoperative success rates.**
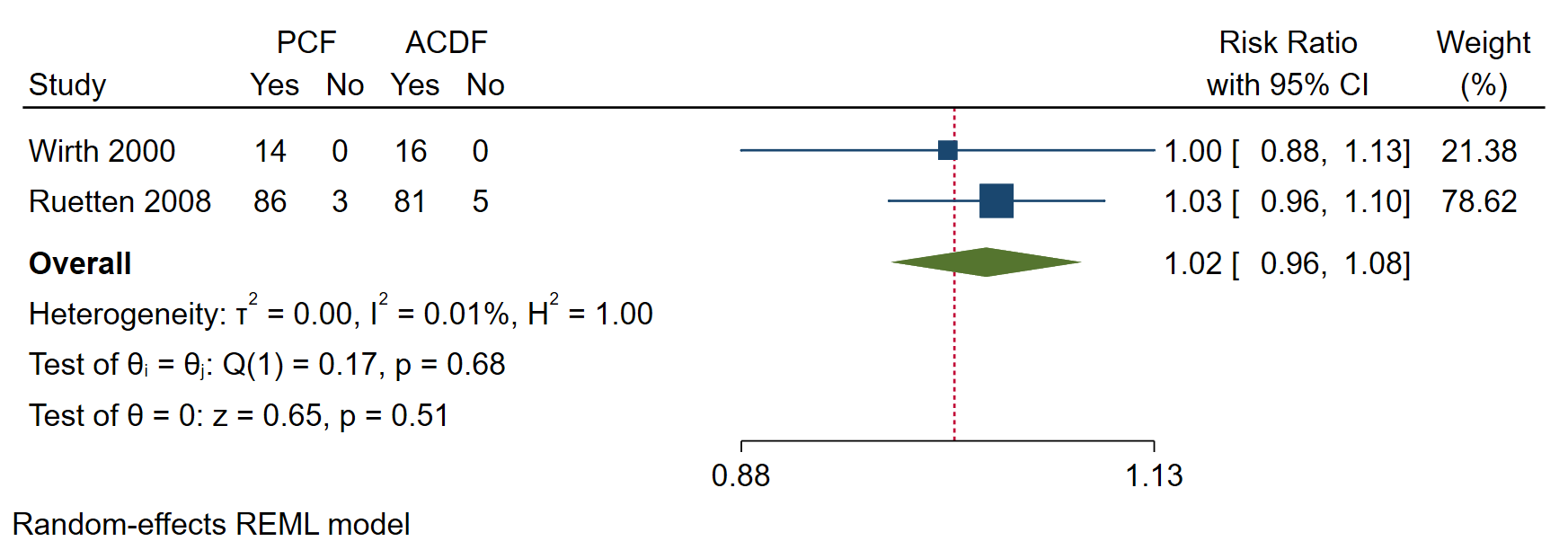


**Supplementary Figure 6.** **PMMA compared with ABG on postoperative success rates.**


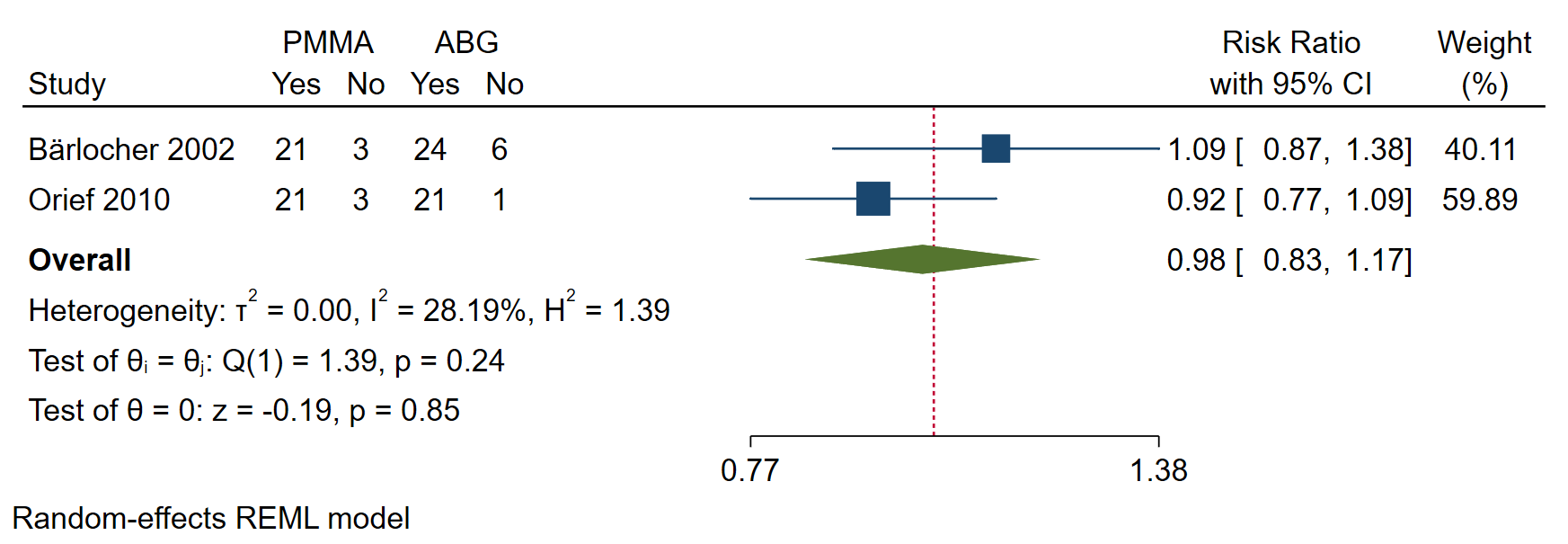


**Supplementary Figure 7.** **PMMA compared with ACDF on postoperative success rates.**


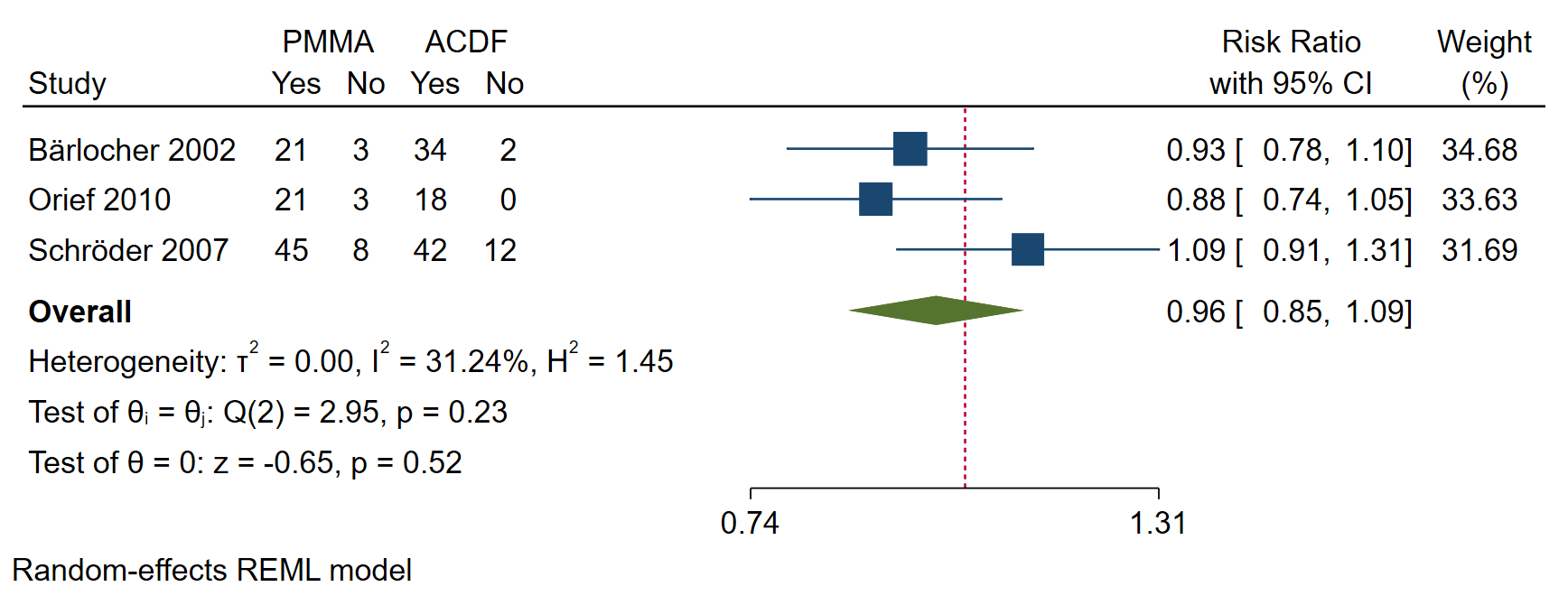


**Supplementary Figure 8.** **ABG compared with ABGP on postoperative complication rates.**
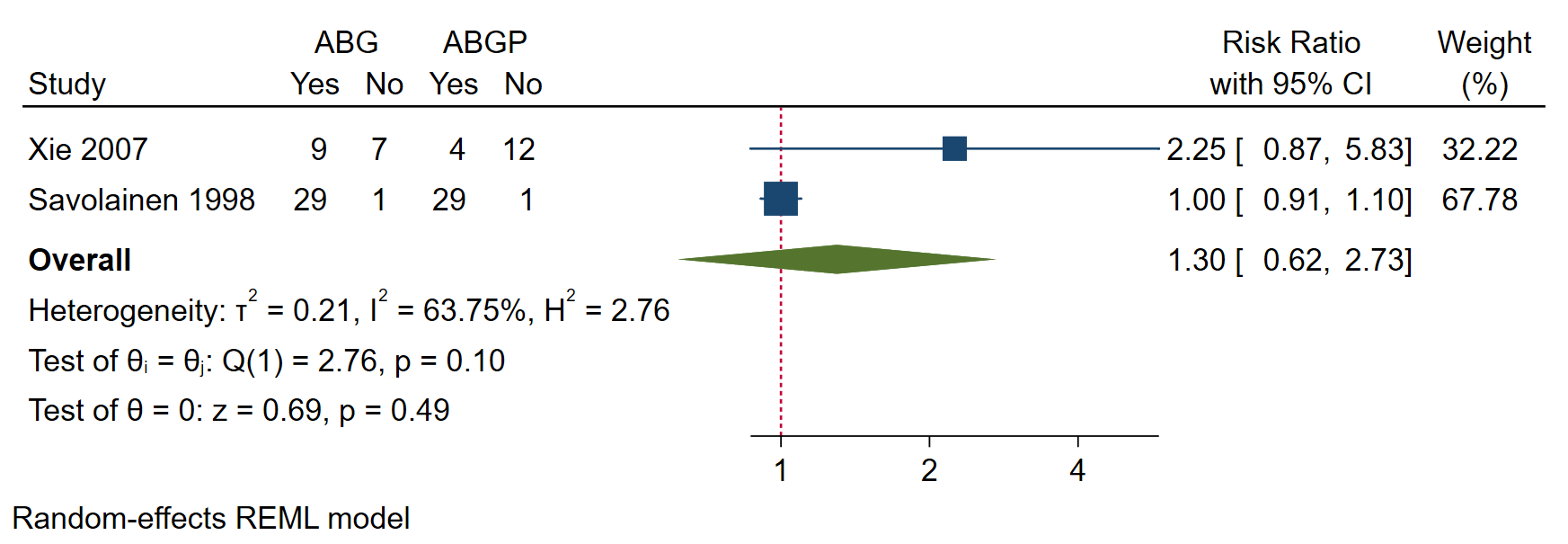


**Supplementary Figure 9.** **ABG compared with ACDF on postoperative complication rates.**


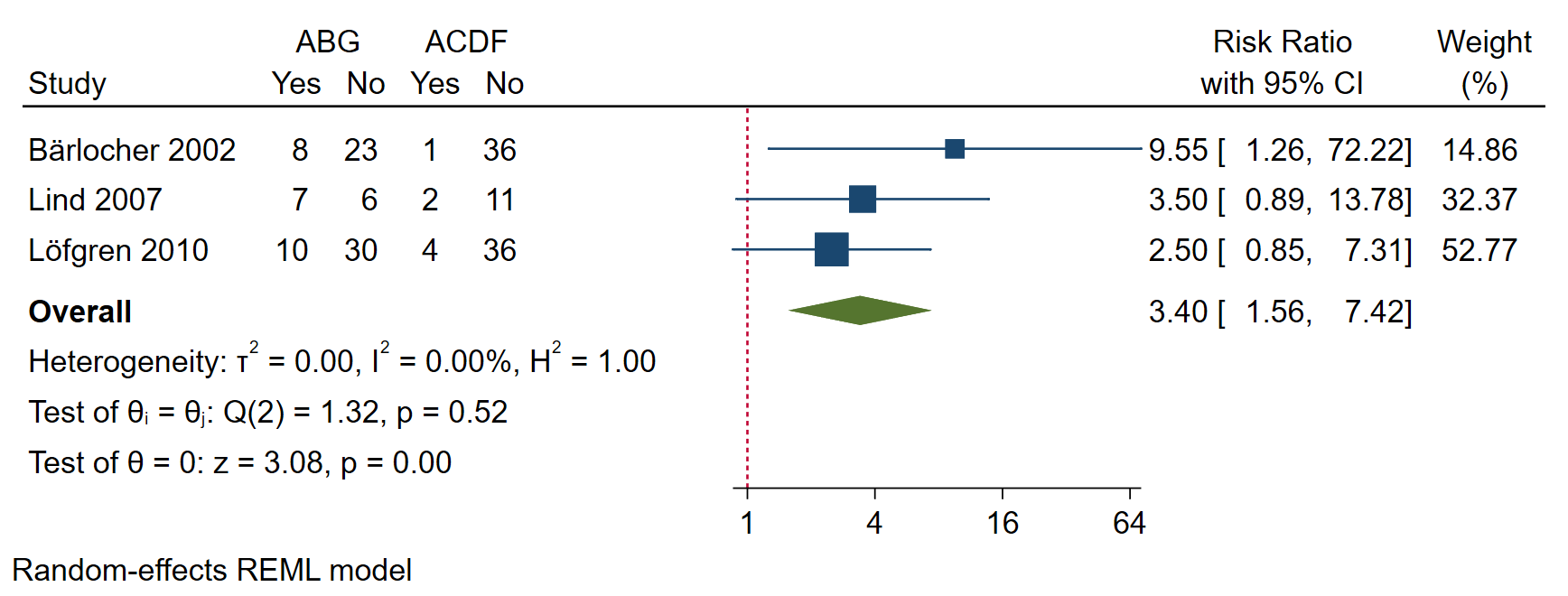


**Supplementary Figure 10.** **ACD compared with ABG on postoperative complication rates.**


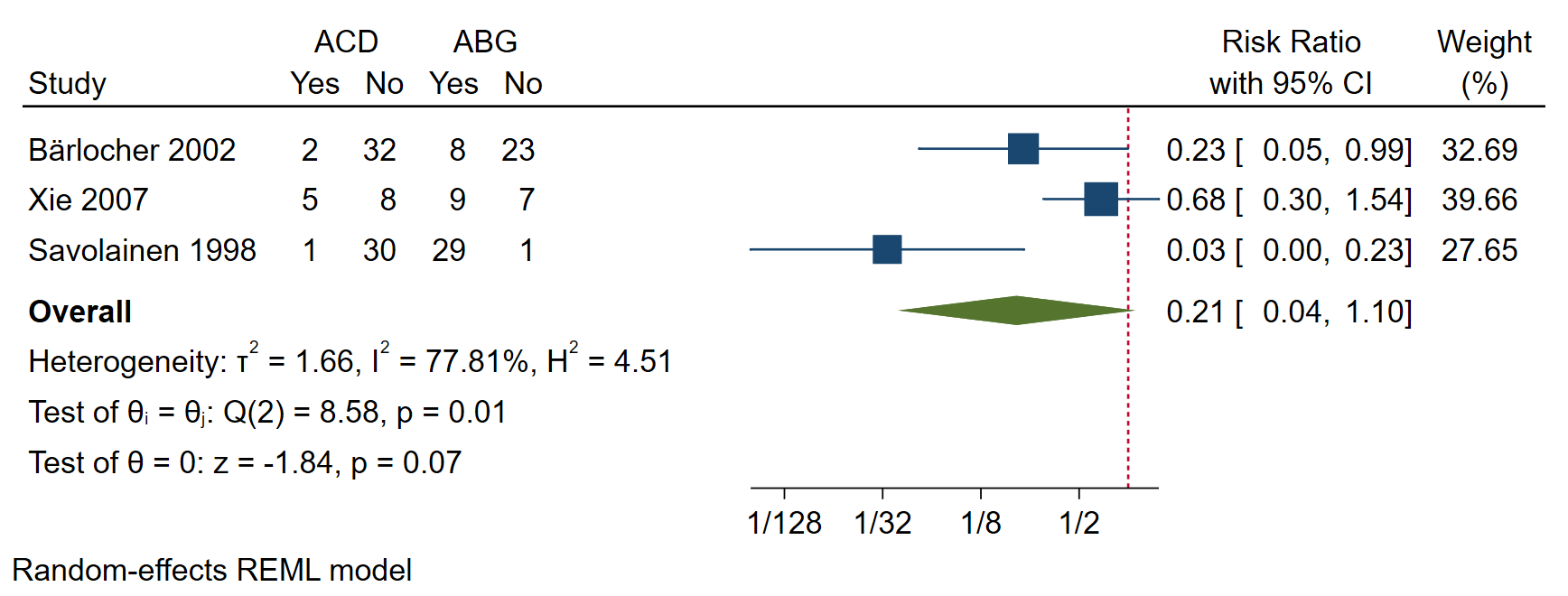


**Supplementary Figure 11.** **ACD compared with ABGP on postoperative complication rates.**


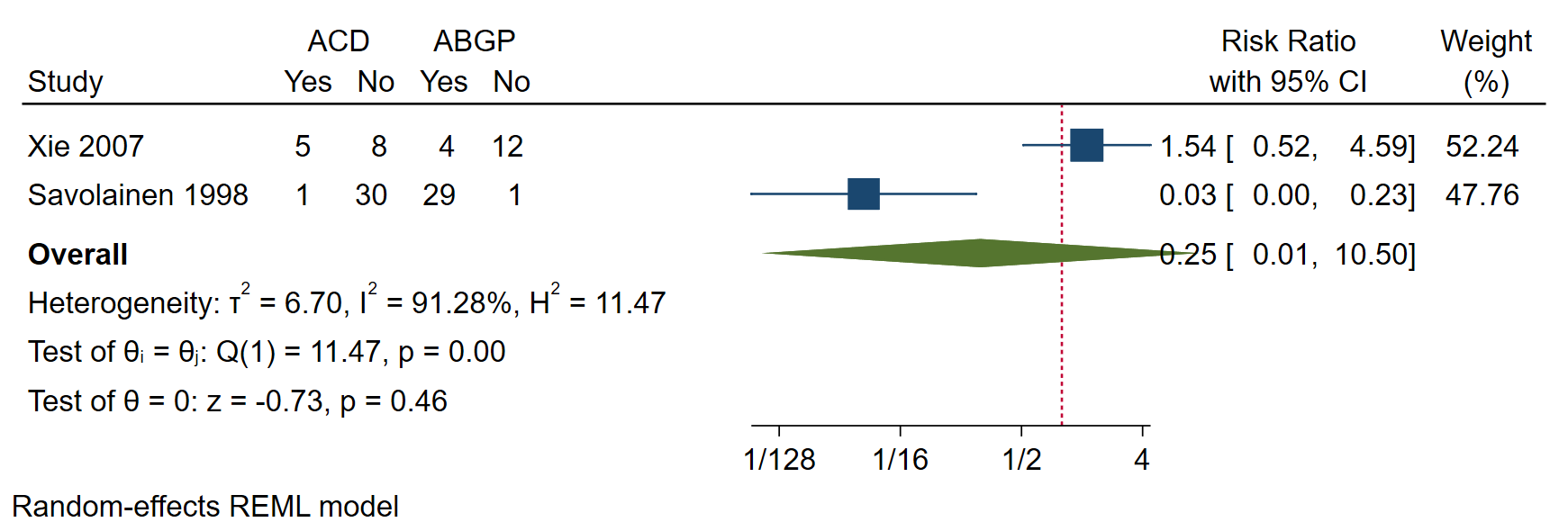


**Supplementary Figure 12.** **ACD compared with ACDF on postoperative complication rates.**


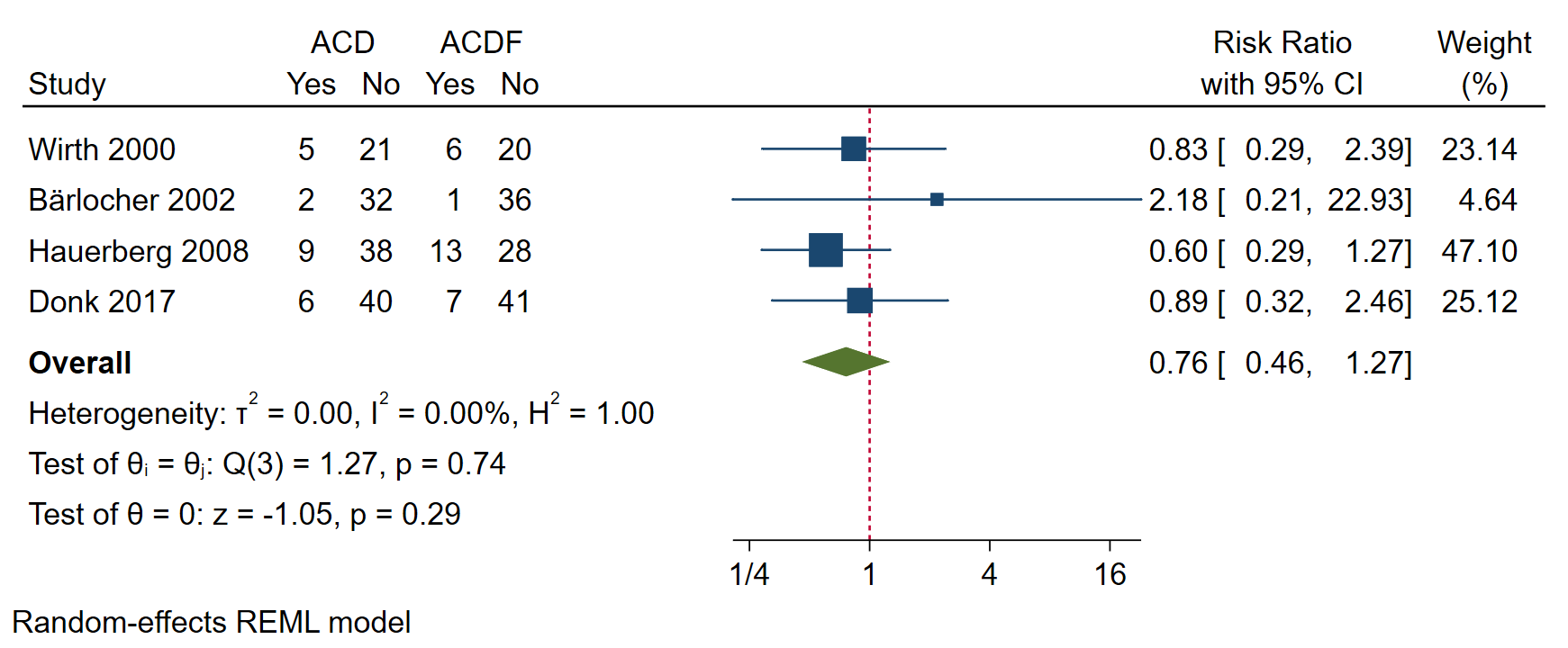


**Supplementary Figure 13.** **CDR compared with ACDF on postoperative complication rates.**
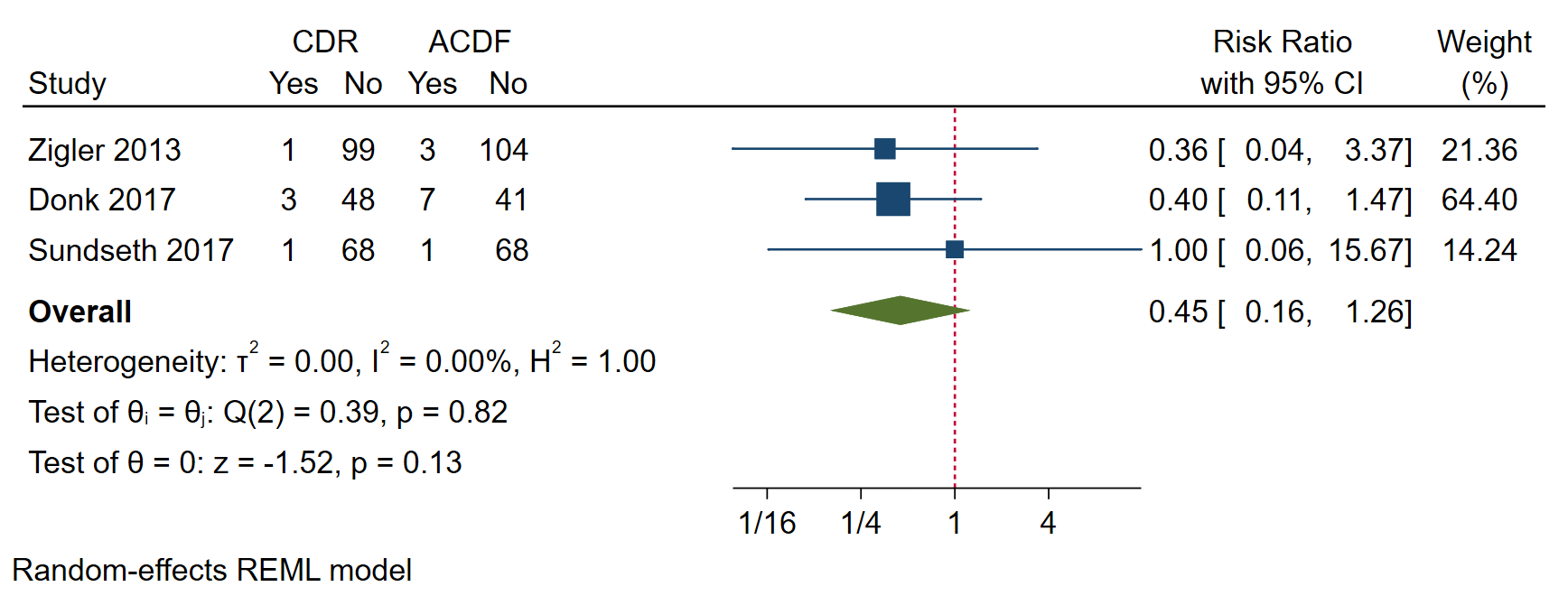


**Supplementary Figure 14.** **PCF compared with ACDF on postoperative complication rates.**
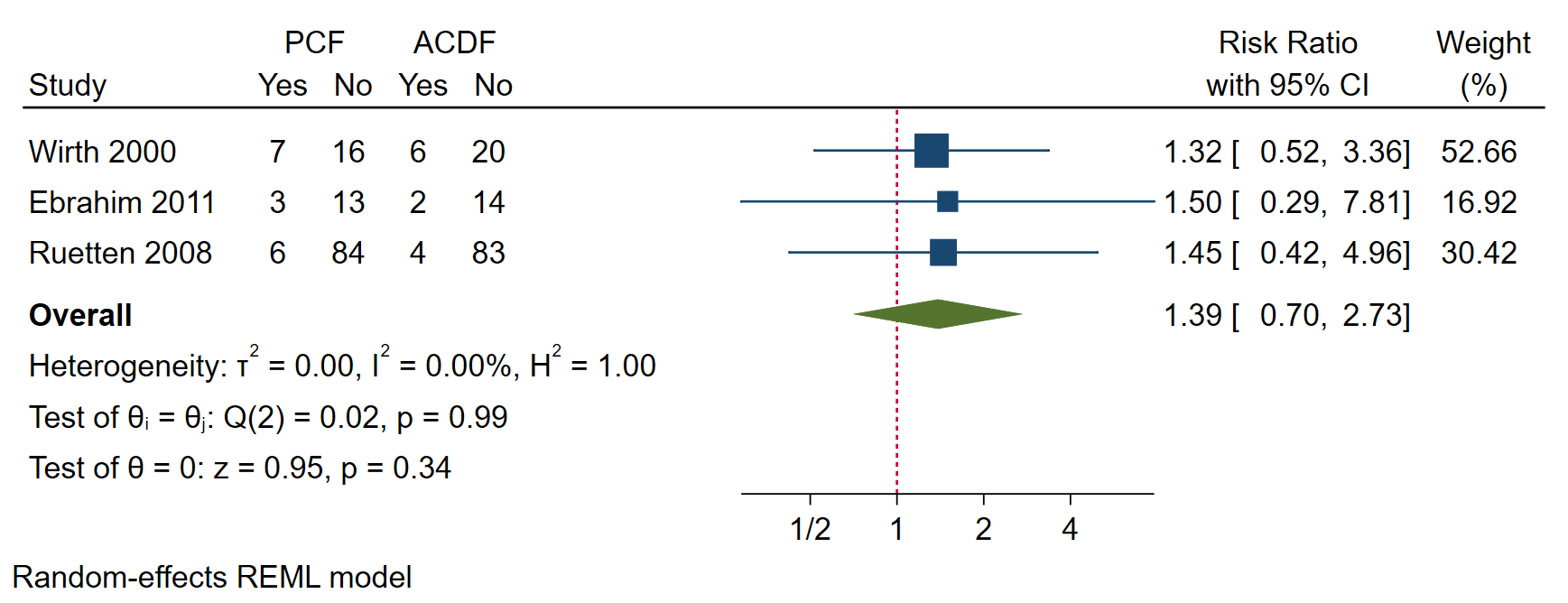


**Supplementary Figure 15.** **PMMA compared with ACDF on postoperative complication rates.**
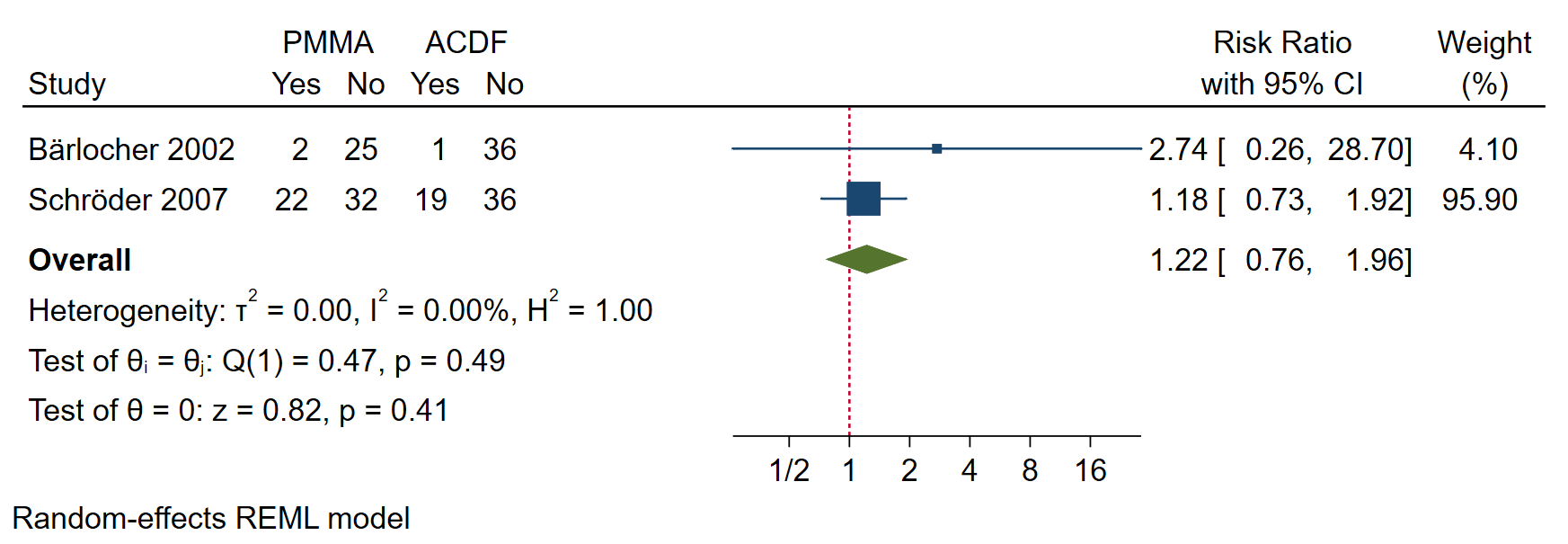


**Supplementary Figure 16.** **Network plots of comparison-based network meta-analyses on post-operative reoperation rates.**
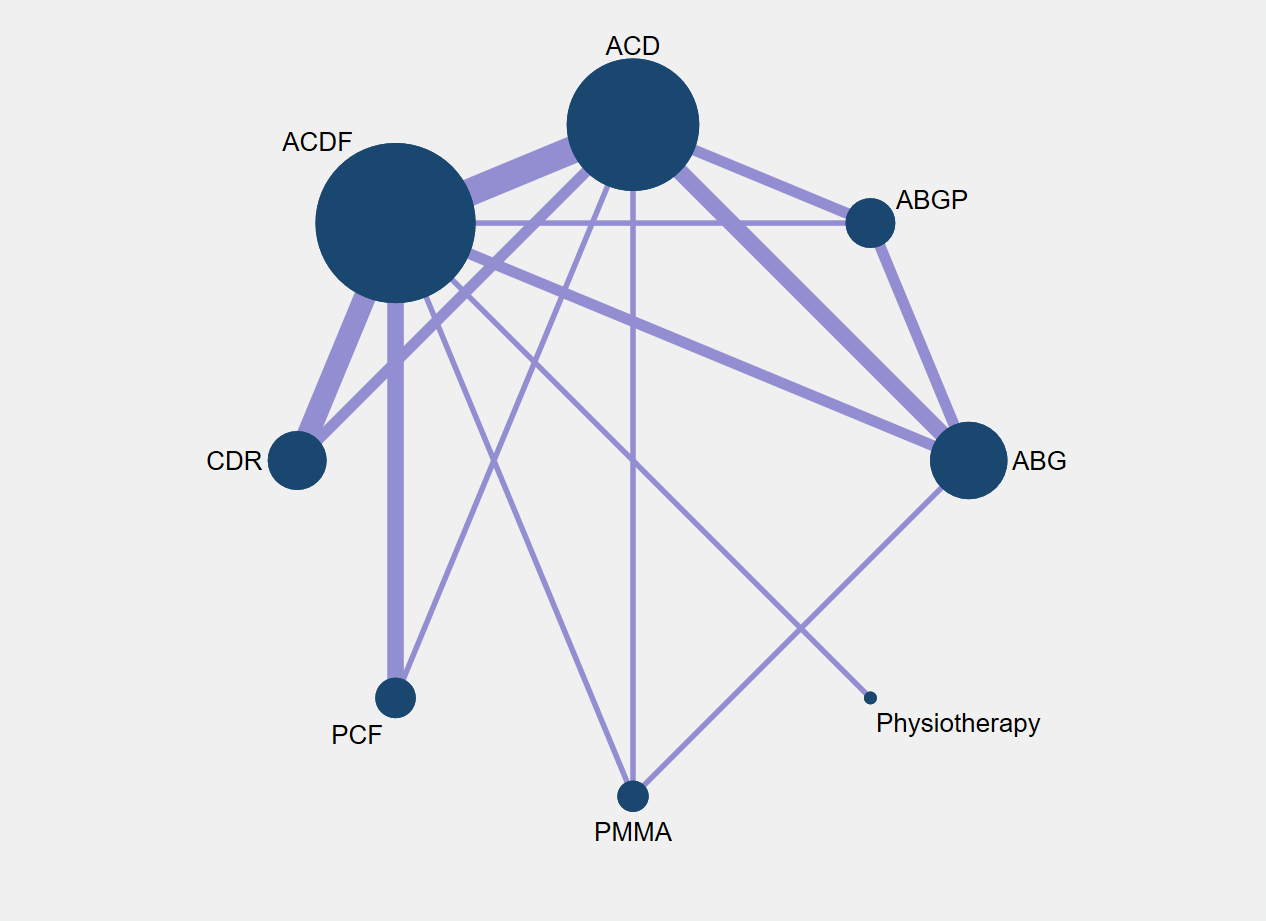
 Each circular node represents a type of treatment. The circle size is proportional to the total number of patients. The width of lines is proportional to the number of studies performing head-to-head comparisons in the same study. ABG: Anterior cervical discectomy with autologous bone graft, ABGP: Anterior cervical discectomy with allograft bone graft plus plating, ACD: Anterior cervical discectomy, ACDF: Anterior cervical discectomy and fusion, CDR: Cervical disc replacement, PCF: Posterior cervical foraminotomy, PMMA: Anterior cervical discectomy with polymethylmethacrylate.

**Supplementary Figure 17.** **ABG compared with ABGP on postoperative reoperation rates.**
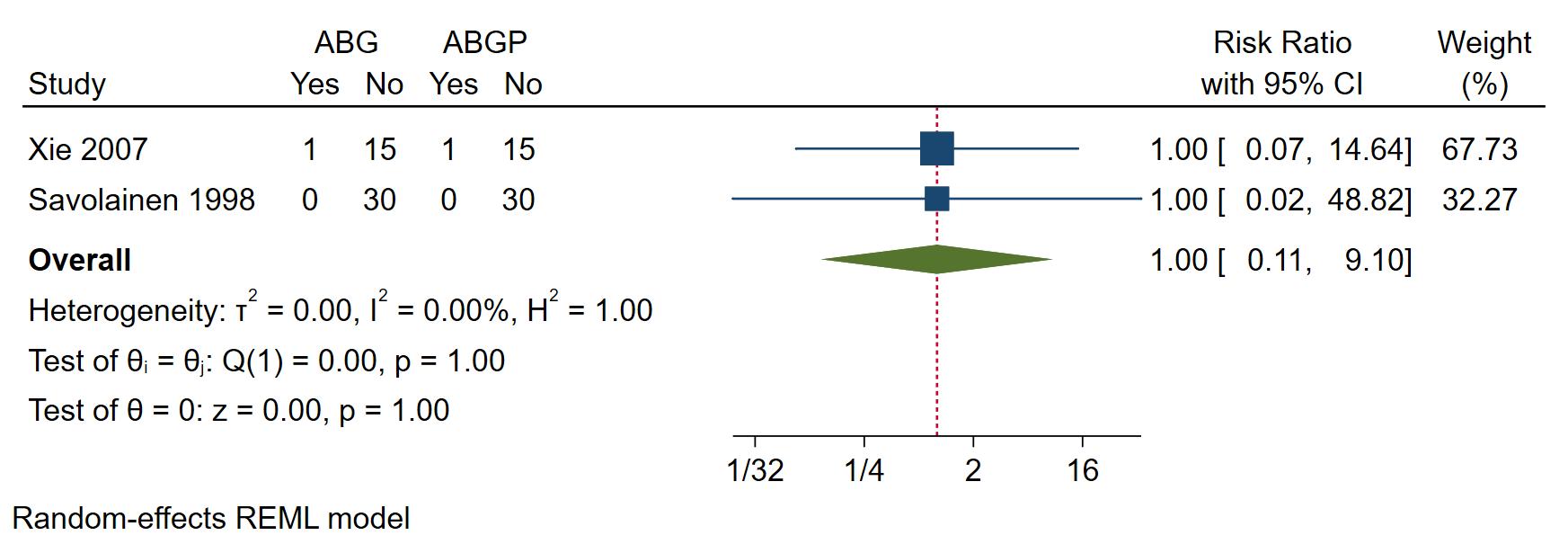


**Supplementary Figure 18.** **ABG compared with ACD on postoperative reoperation rates.**
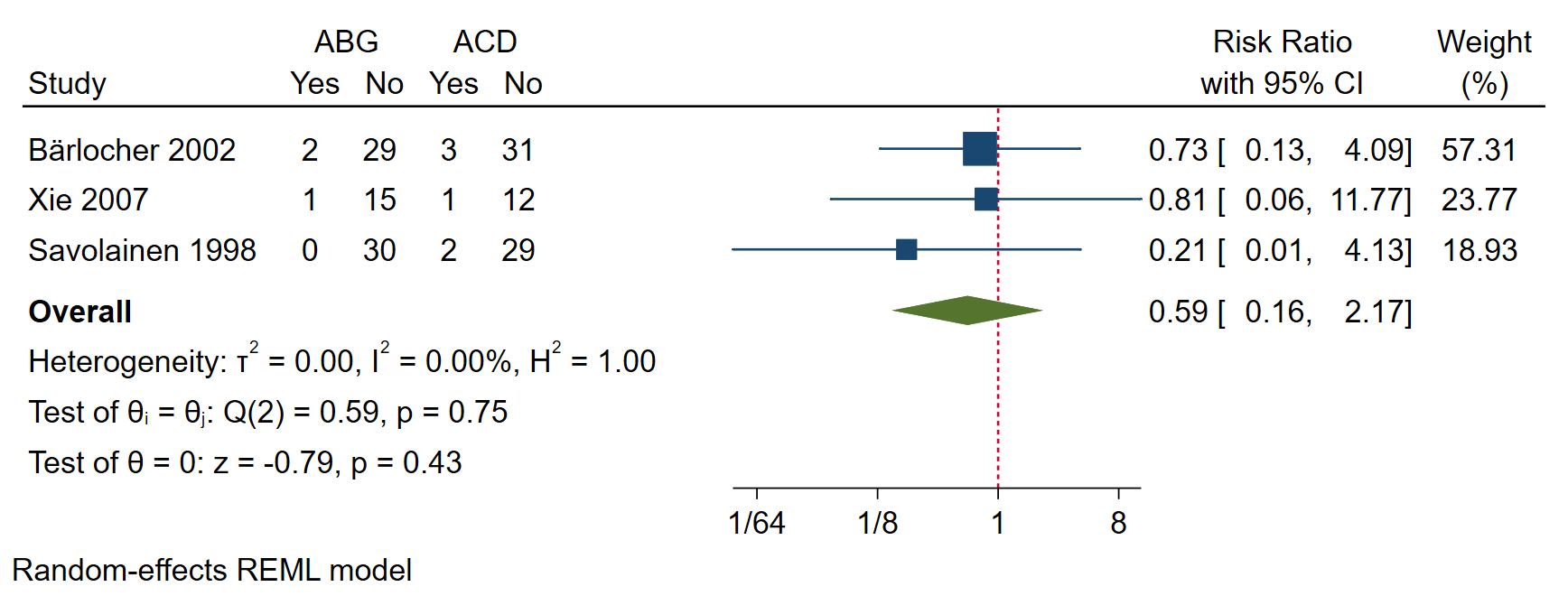


**Supplementary Figure 19.** **ABG compared with ACDF on postoperative reoperation rates.**
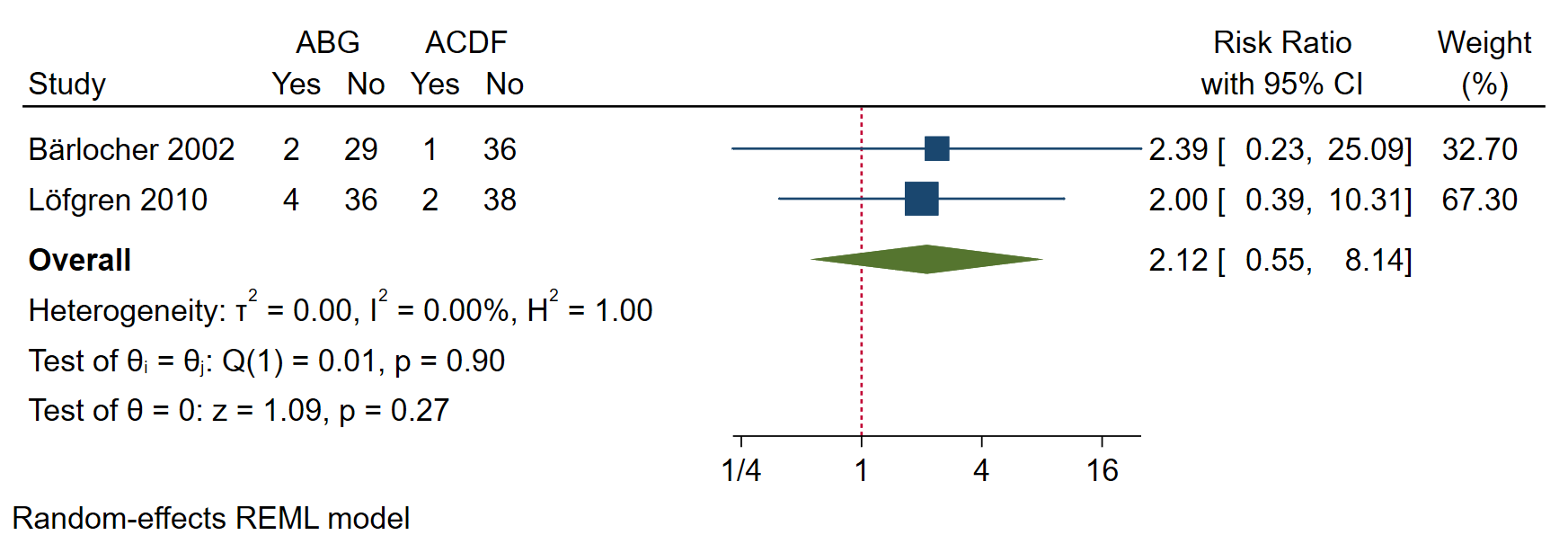


**Supplementary Figure 20.** **ACD compared with ABGP on postoperative reoperation rates.**
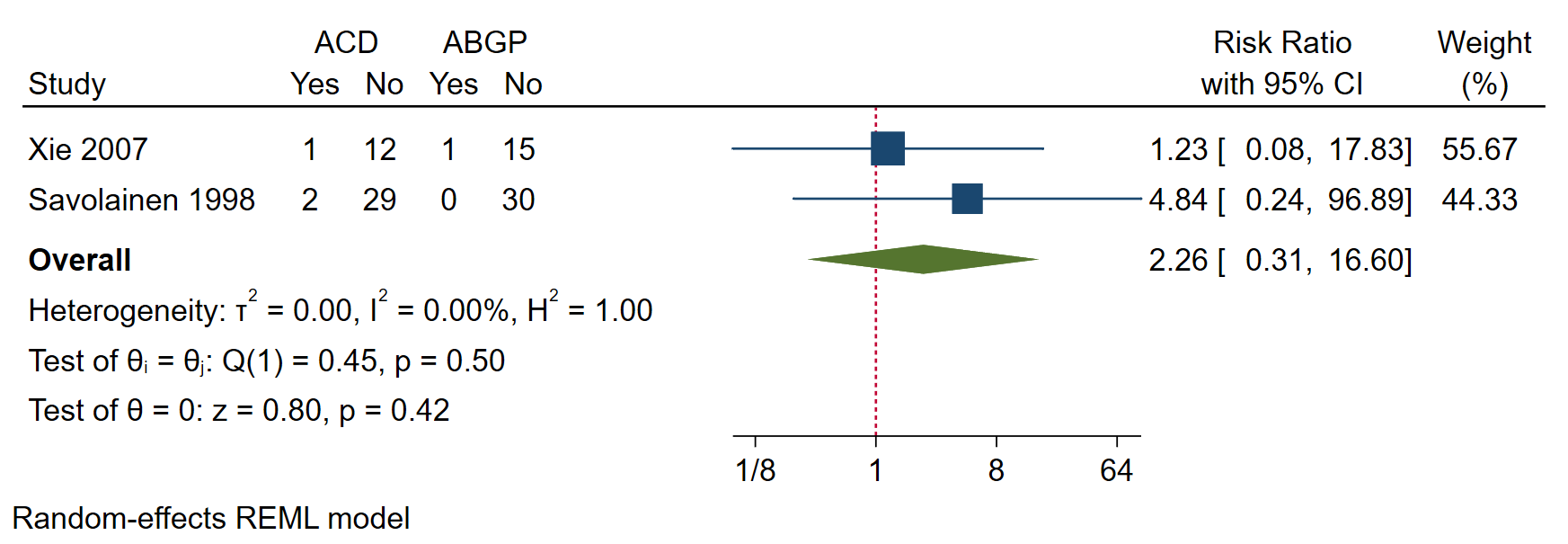


**Supplementary Figure 21.** **ACD compared with ACDF on postoperative reoperation rates.**
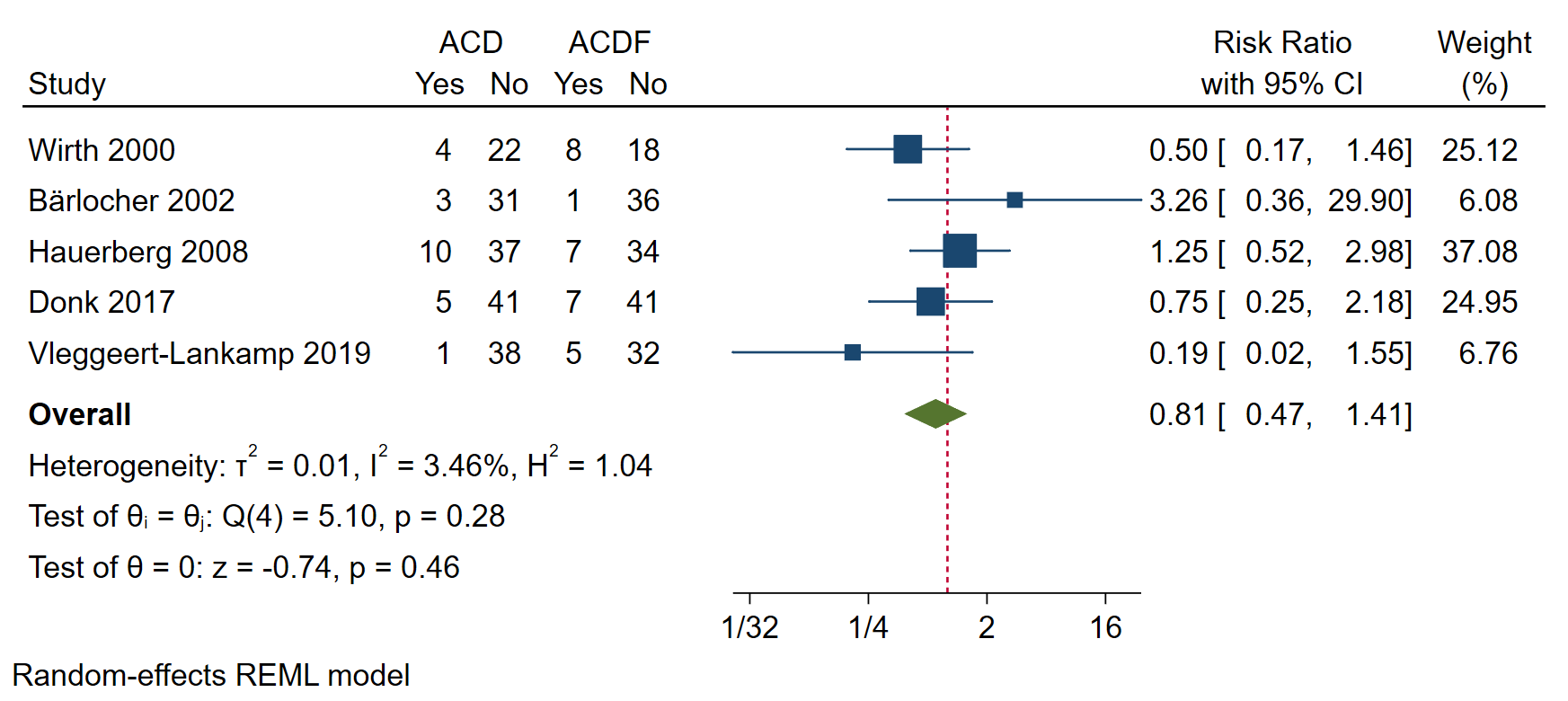


**Supplementary Figure 22.** **ACD compared with CDR on postoperative reoperation rates.**
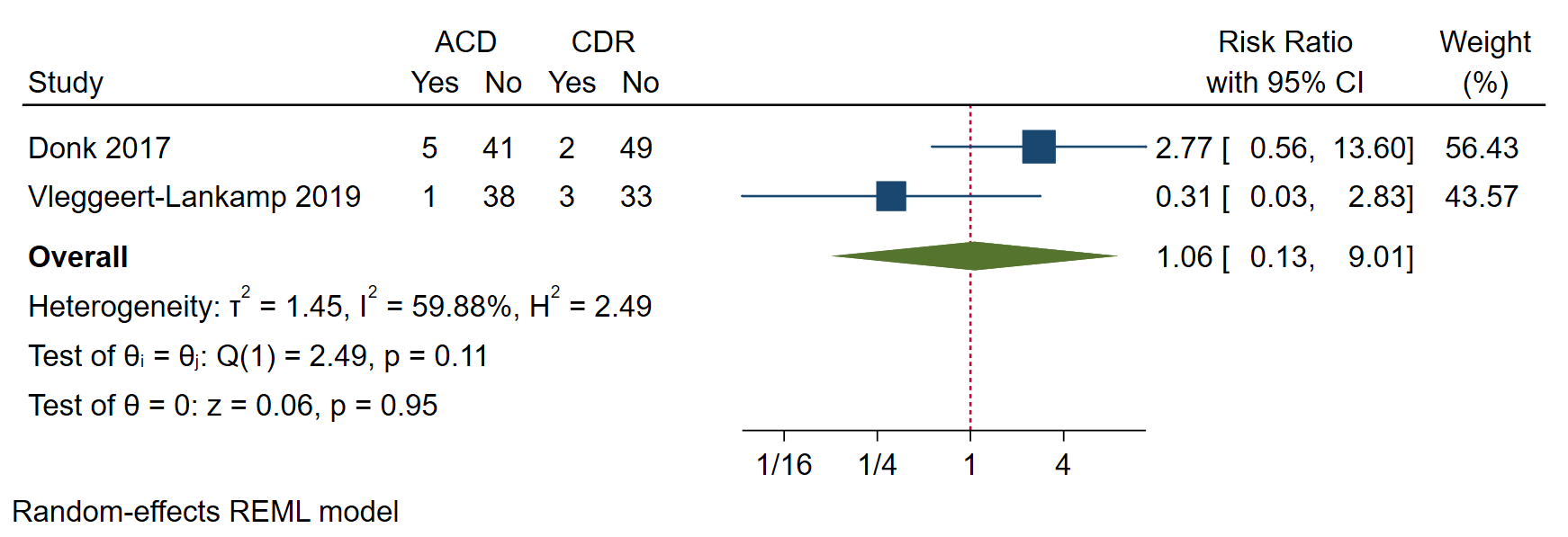


**Supplementary Figure 23.** **CDR compared with ACDF on postoperative reoperation rates.**
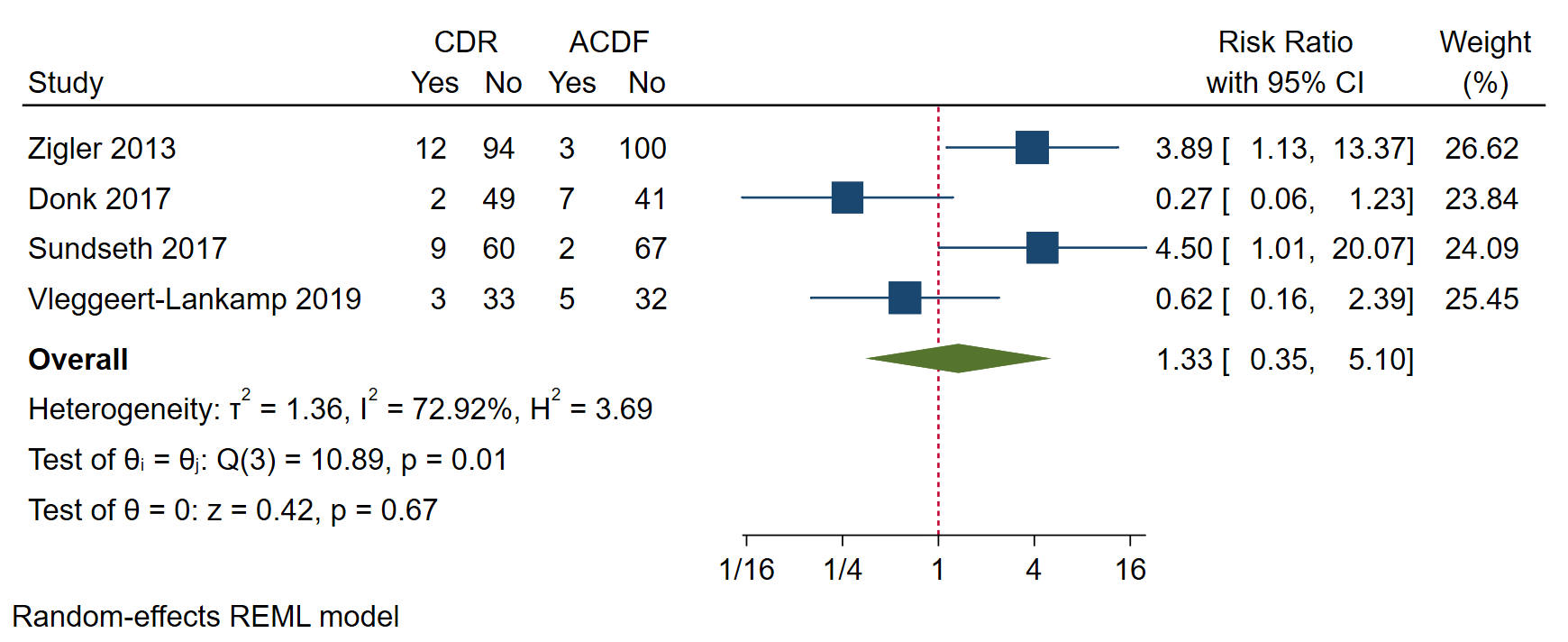


**Supplementary Figure 24.** **PCF compared with ACDF on postoperative reoperation rates.**
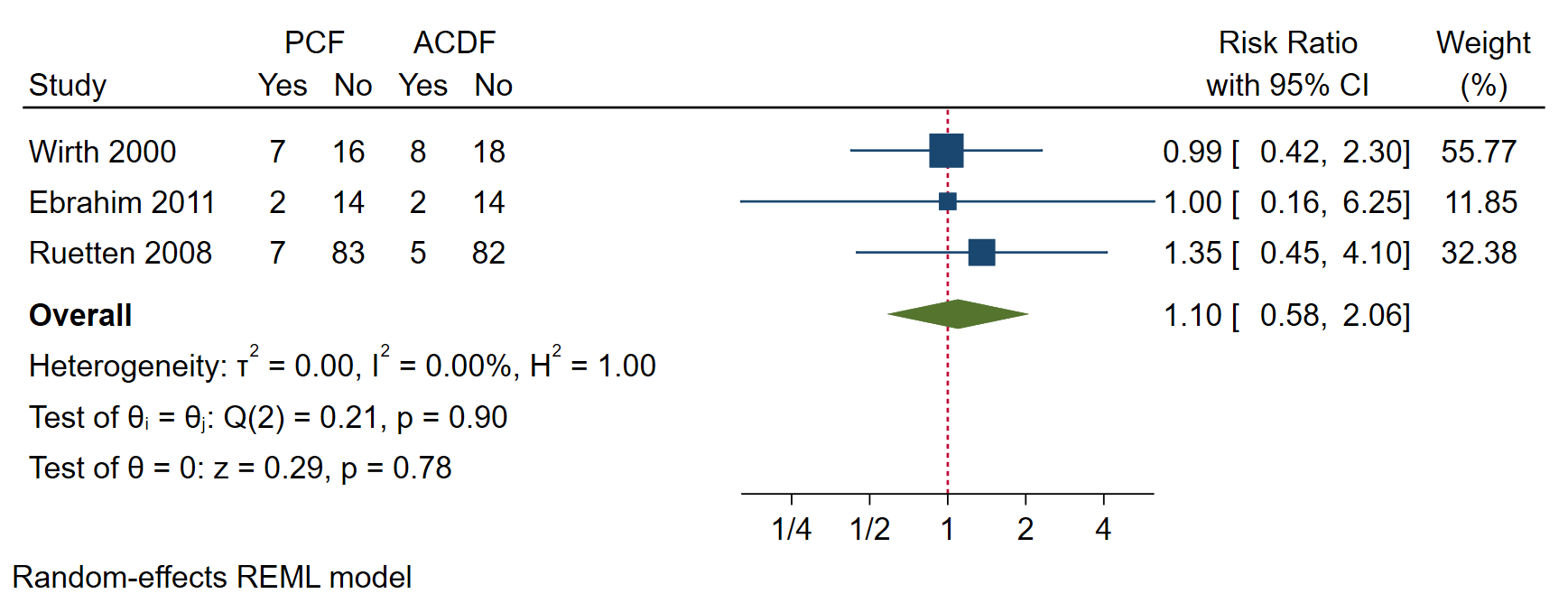


**Supplementary Figure 25.** **Network plots of comparison-based network meta-analyses on post-operative work status.**
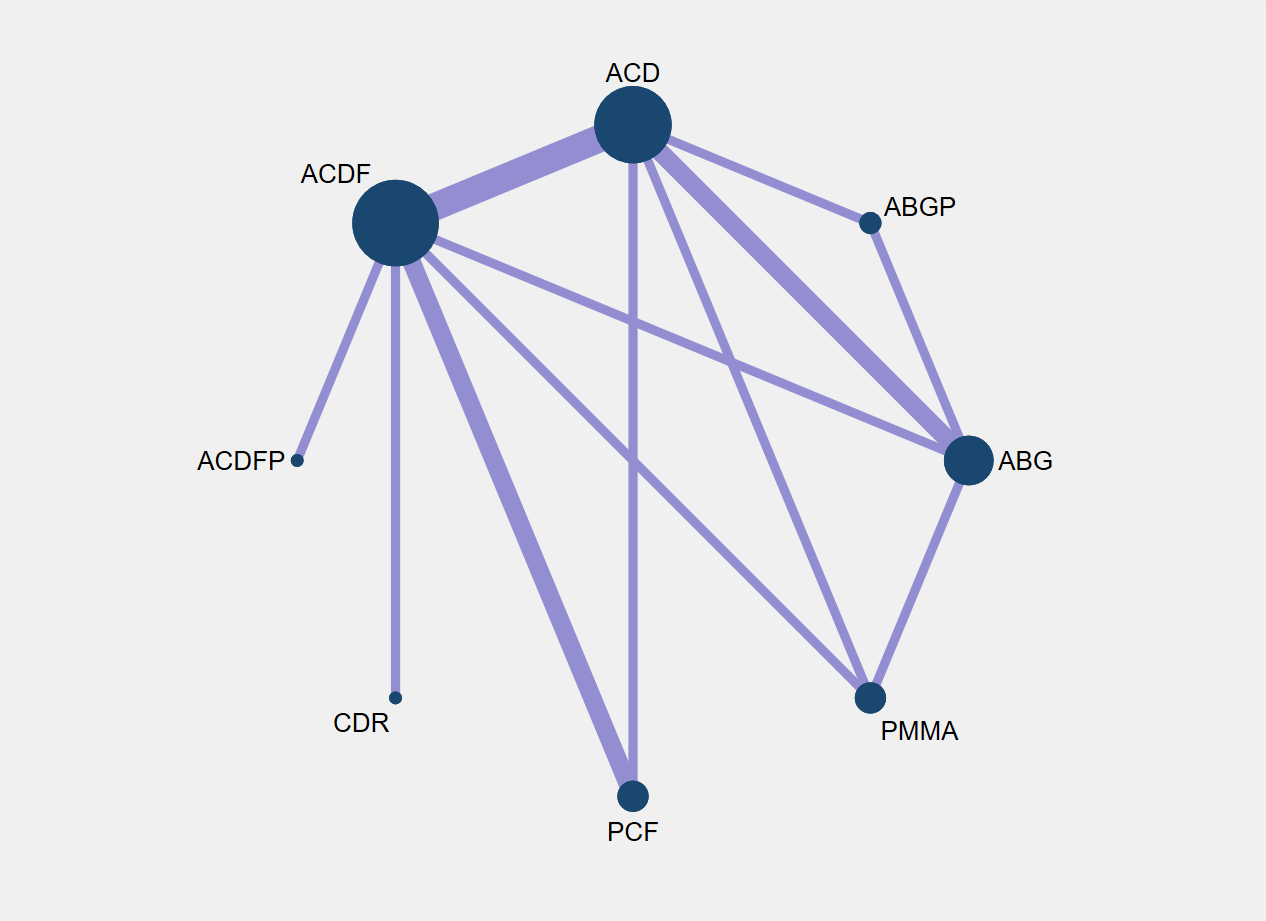
 Each circular node represents a type of treatment. The circle size is proportional to the total number of patients. The width of lines is proportional to the number of studies performing head-to-head comparisons in the same study. ABG: Anterior cervical discectomy with autologous bone graft, ABGP: Anterior cervical discectomy with allograft bone graft plus plating, ACD: Anterior cervical discectomy, ACDF: Anterior cervical discectomy and fusion, ACDFP: Anterior cervical discectomy with fusion and additional plating, CDR: Cervical disc replacement, PCF: Posterior cervical foraminotomy, PMMA: Anterior cervical discectomy with polymethylmethacrylate.

**Supplementary Figure 26.** **ACD compared with ABG on postoperative work status.**
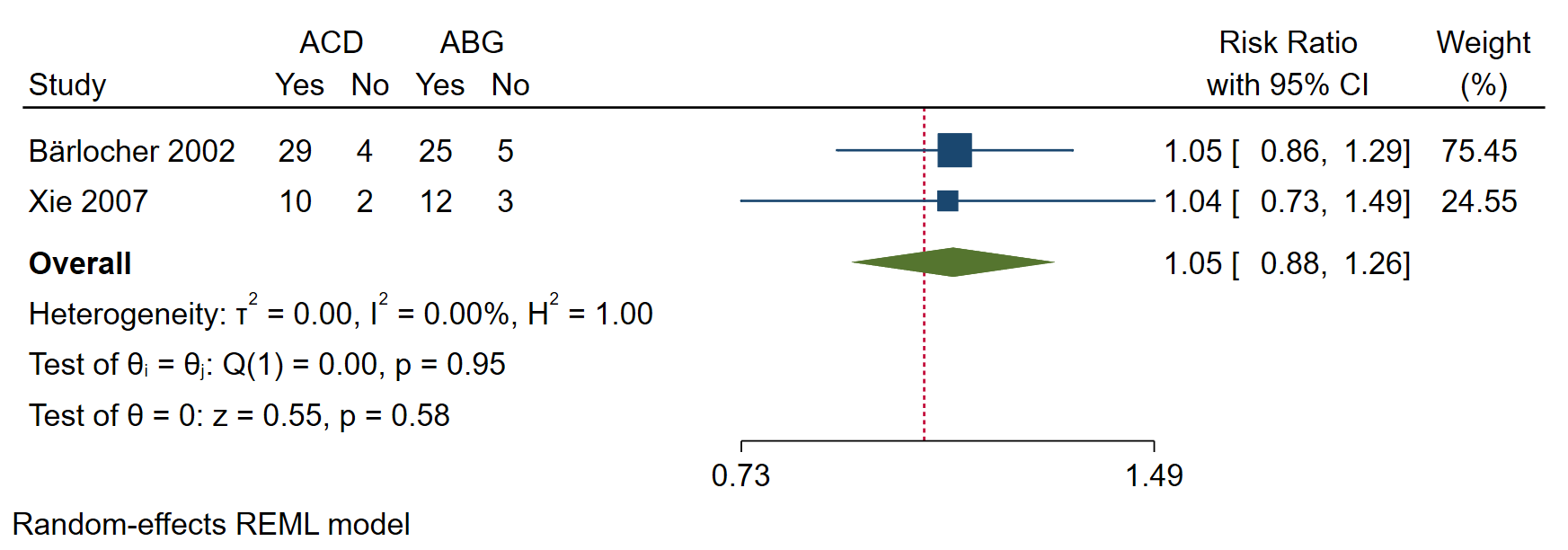


**Supplementary Figure 27.** **ACD compared with ACDF on postoperative work status.**
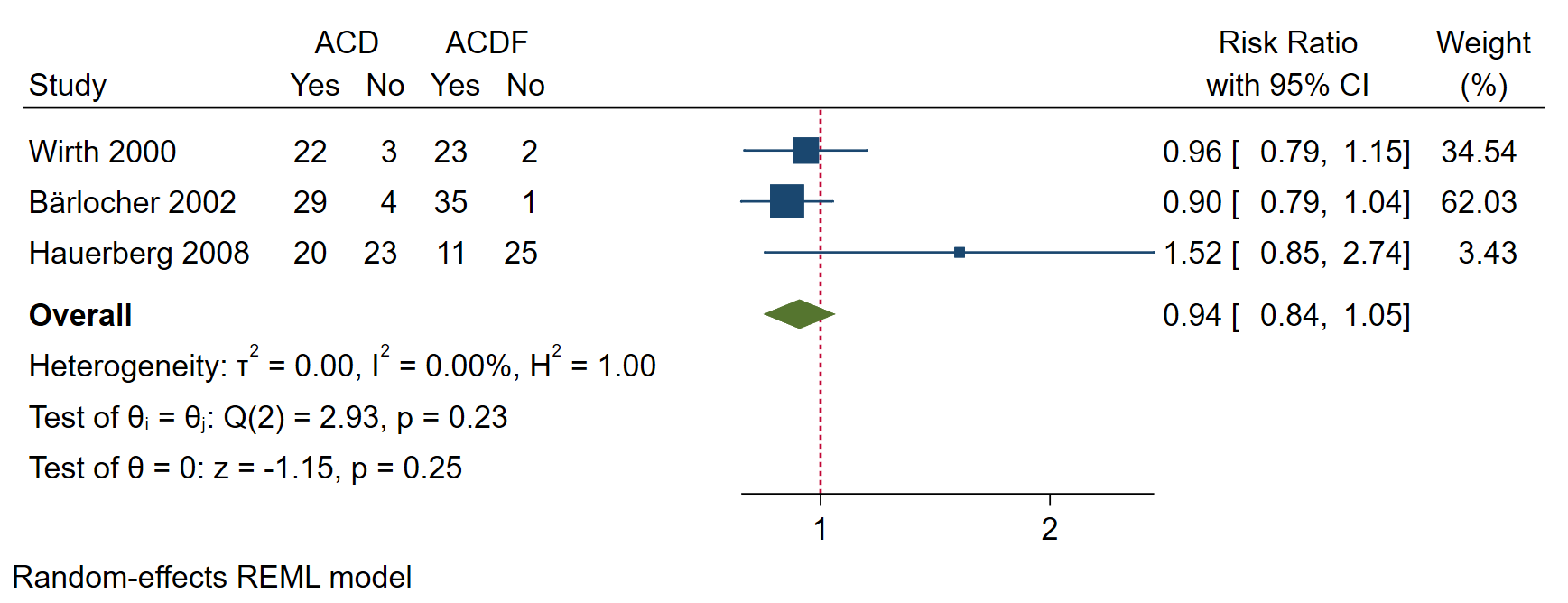


**Supplementary Figure 28.** **PCF compared with ACDF on postoperative work status.**
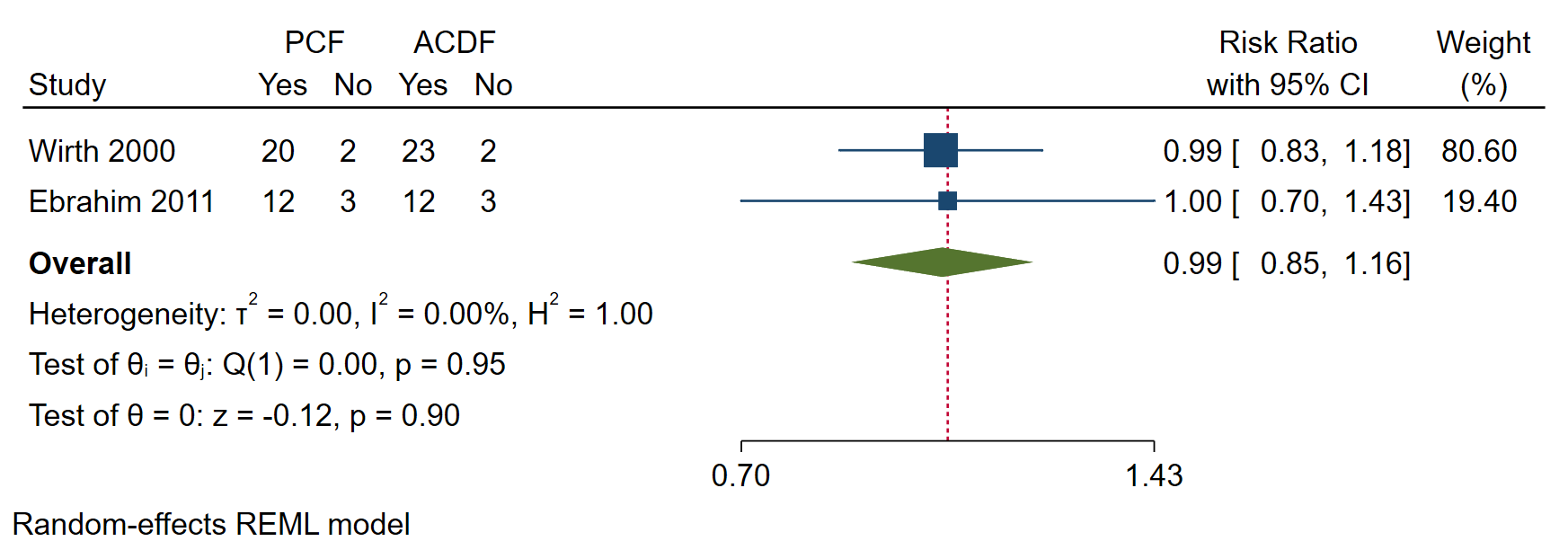


**Supplementary Figure 29.** **Network plots of comparison-based network meta-analyses on scores for arm pain.**
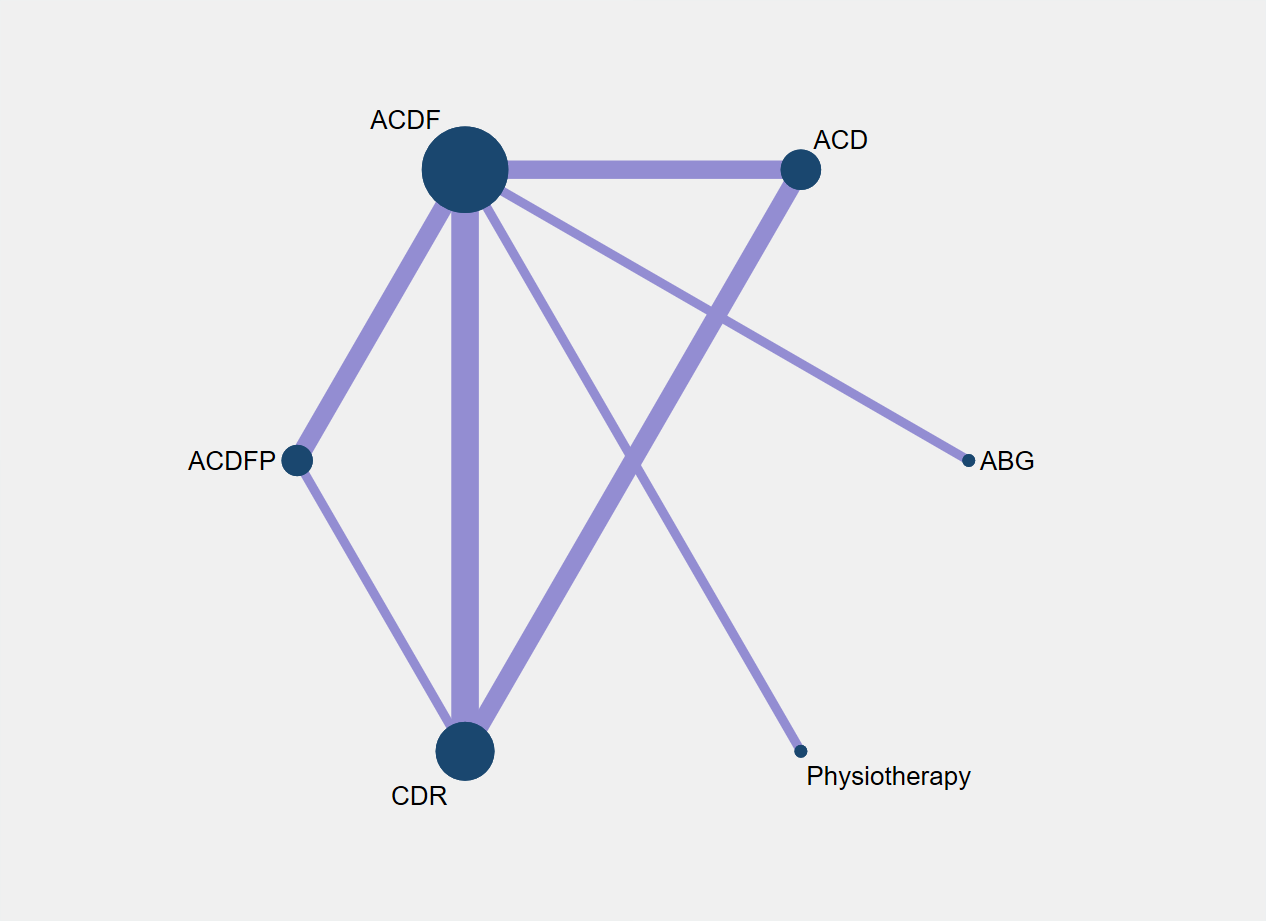
 Each circular node represents a type of treatment. The circle size is proportional to the total number of patients. The width of lines is proportional to the number of studies performing head-to-head comparisons in the same study. ABG: Anterior cervical discectomy with autologous bone graft, ACD: Anterior cervical discectomy, ACDF: Anterior cervical discectomy and fusion, ACDFP: Anterior cervical discectomy with fusion and additional plating, CDR: Cervical disc replacement.

**Supplementary Figure 30.** **Network plots of comparison-based network meta-analyses on scores for neck pain.**
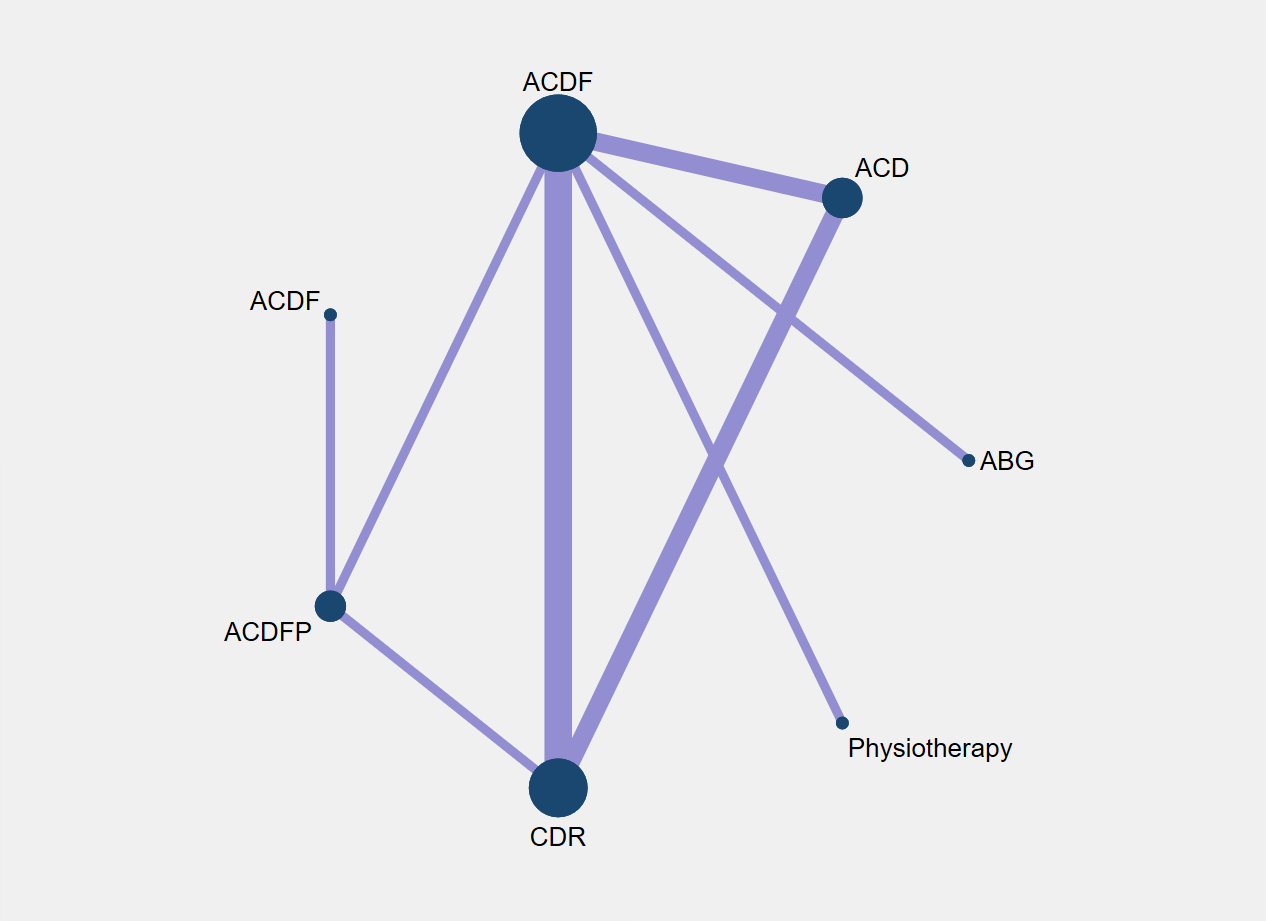
 Each circular node represents a type of treatment. The circle size is proportional to the total number of patients. The width of lines is proportional to the number of studies performing head-to-head comparisons in the same study. ABG: Anterior cervical discectomy with autologous bone graft, ABGP: Anterior cervical discectomy with allograft bone graft plus plating, ACD: Anterior cervical discectomy, ACDF: Anterior cervical discectomy and fusion, ACDFP: Anterior cervical discectomy with fusion and additional plating, CDR: Cervical disc replacement, PMMA: Anterior cervical discectomy with polymethylmethacrylate.

**Supplementary Figure 31.** **ACD compared with ACDF on scores for arm pain.**


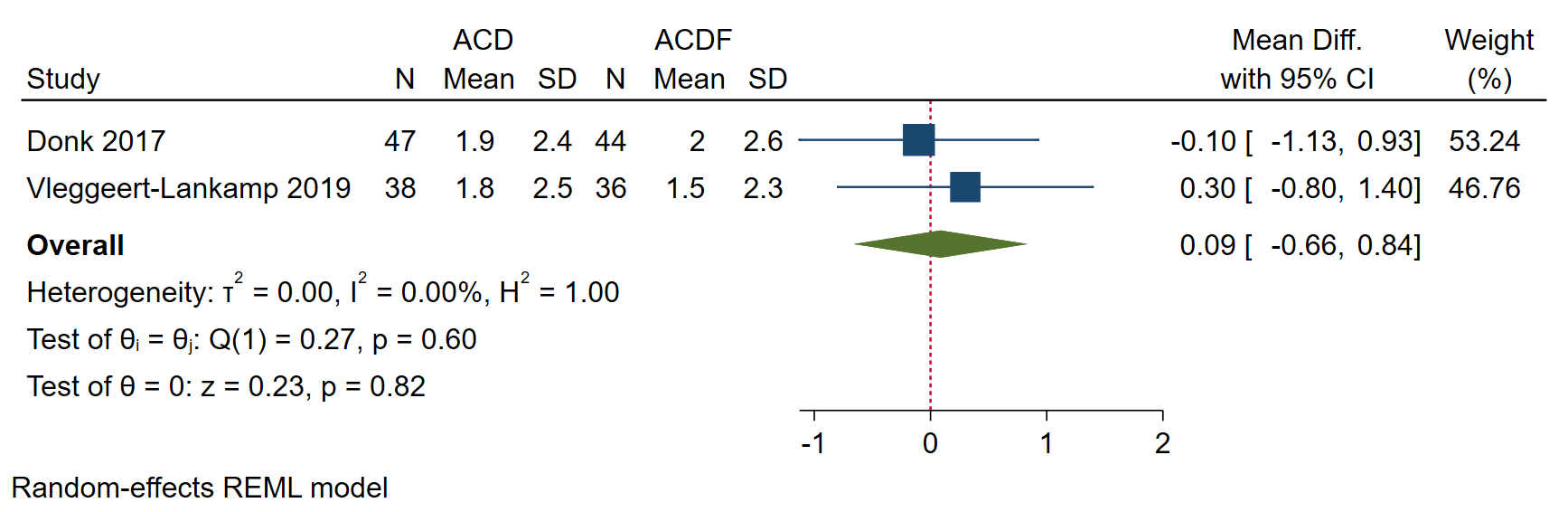


**Supplementary Figure 32.** **ACD compared with CDR on scores for arm pain.**


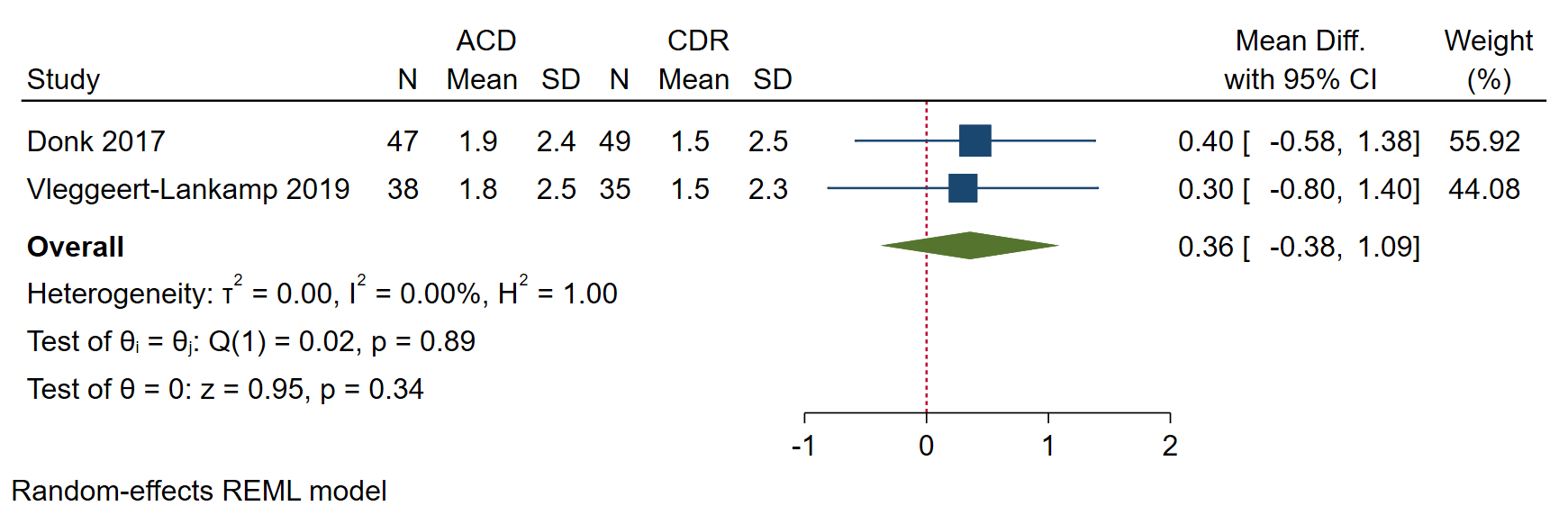


**Supplementary Figure 33.** **CDR compared with ACDF on scores for arm pain.**
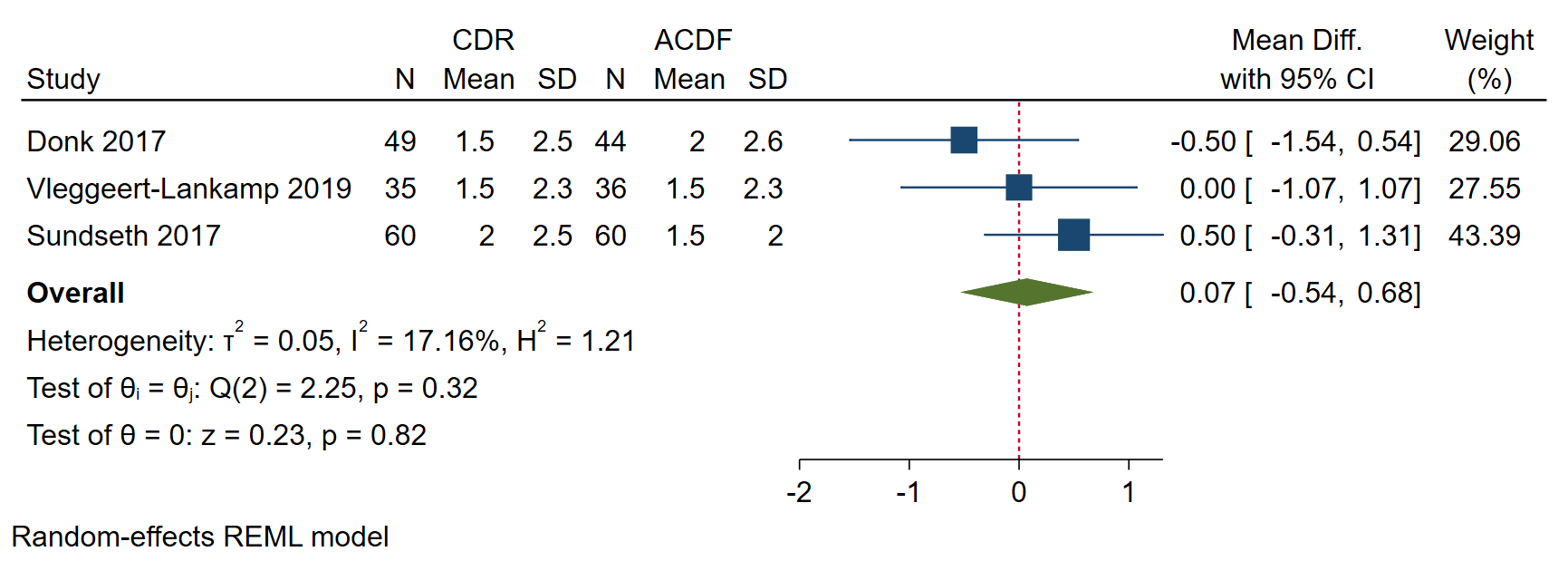


**Supplementary Figure 34.** **CDR compared with ACDF on scores for neck pain.**
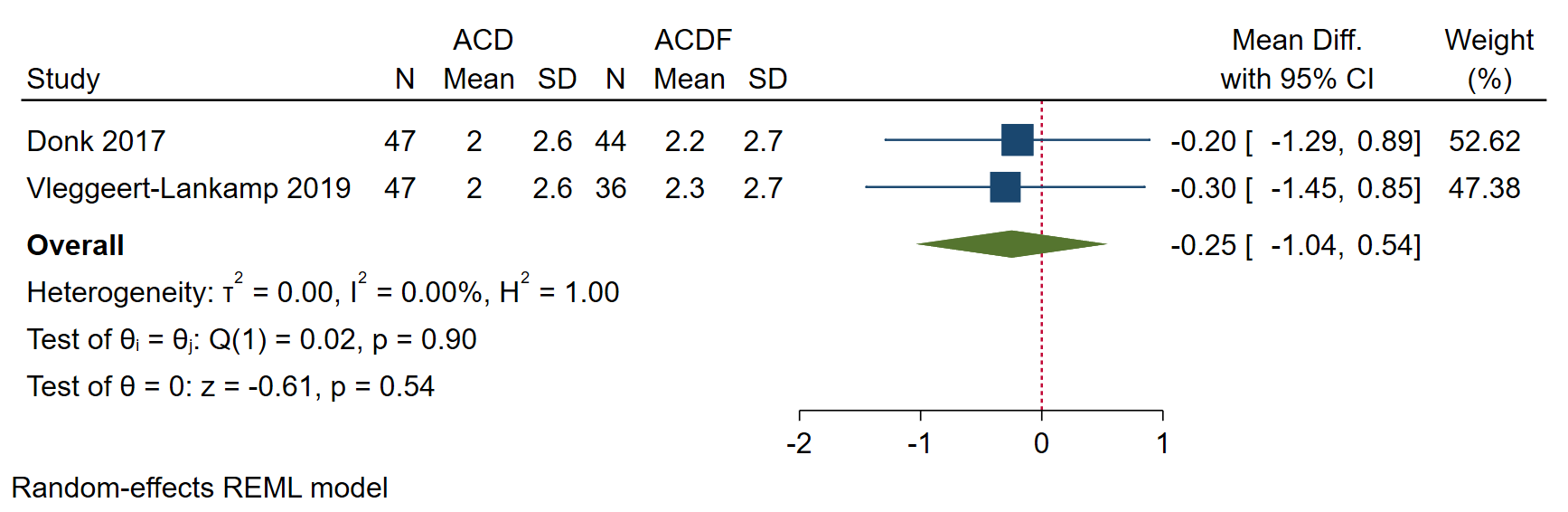


**Supplementary Figure 35.** **ACD compared with CDR on scores for neck pain.**
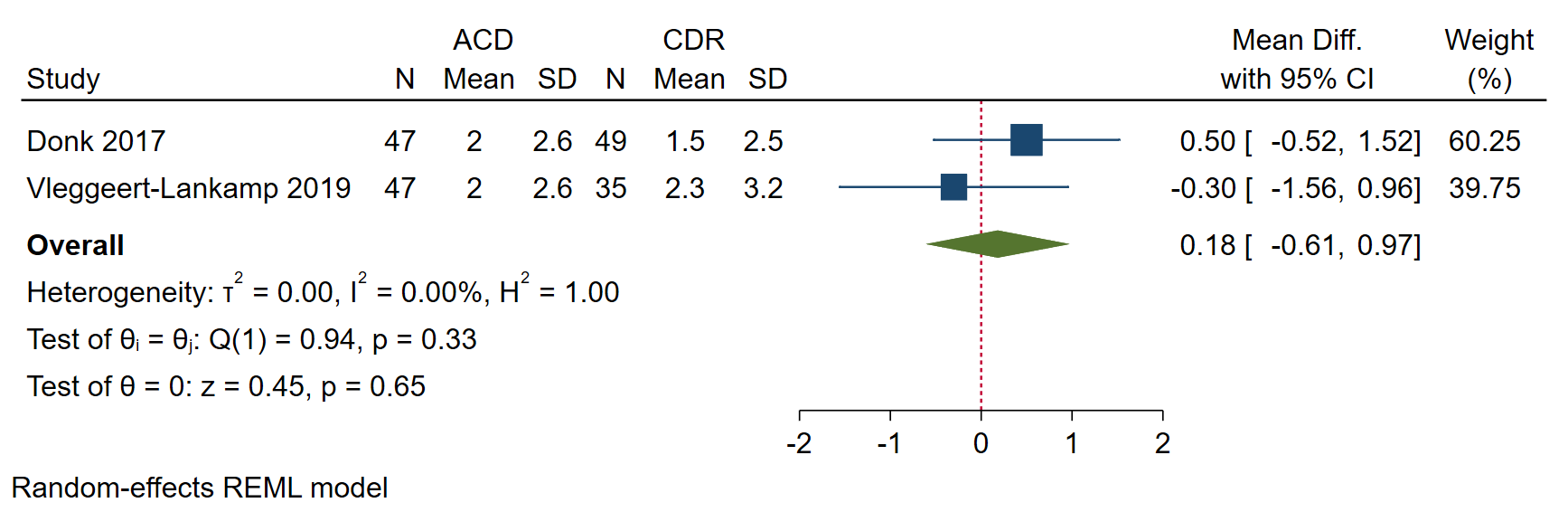


**Supplementary Figure 36.** **CDR compared with ACDF on scores for neck pain.**
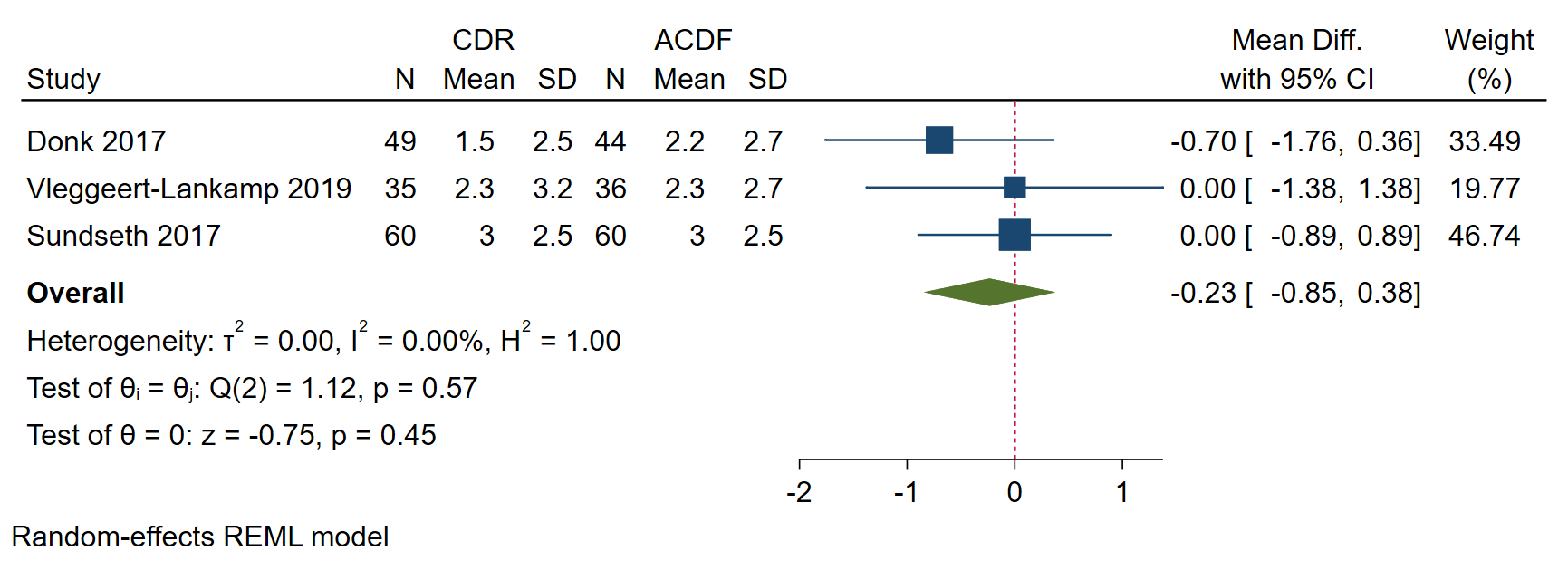


**Supplementary Figure 37.** **Forest plots depicting the direct and indirect results of scores for arm and neck pain of head-to-head comparisons.**


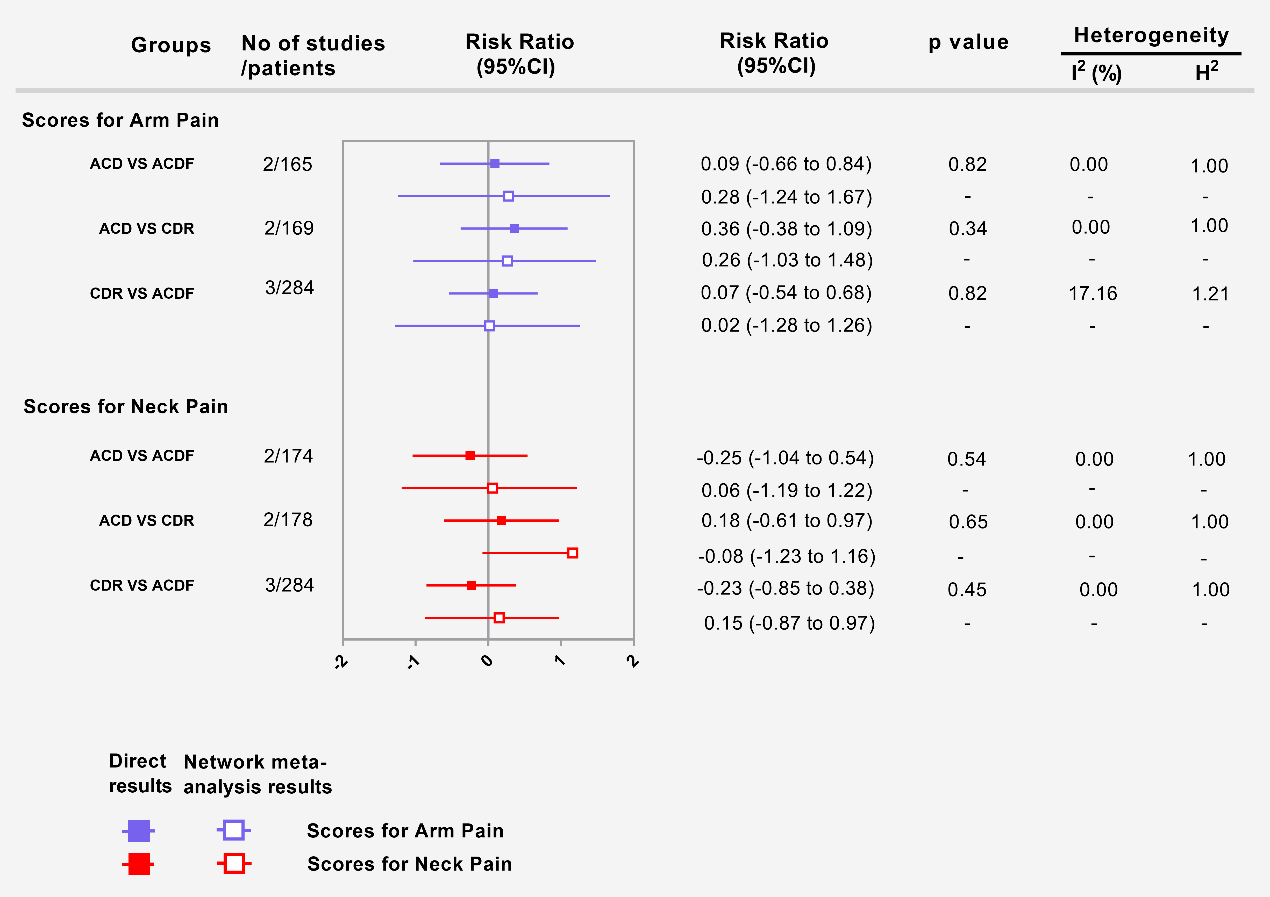
 ACD: Anterior cervical discectomy, ACDF: Anterior cervical discectomy and fusion, CDR: Cervical disc replacement. *Values in brackets are 95% CI.

**Supplementary Figure 38.** **Network plots of comparison-based network meta-analyses on neck disability index (NDI).**
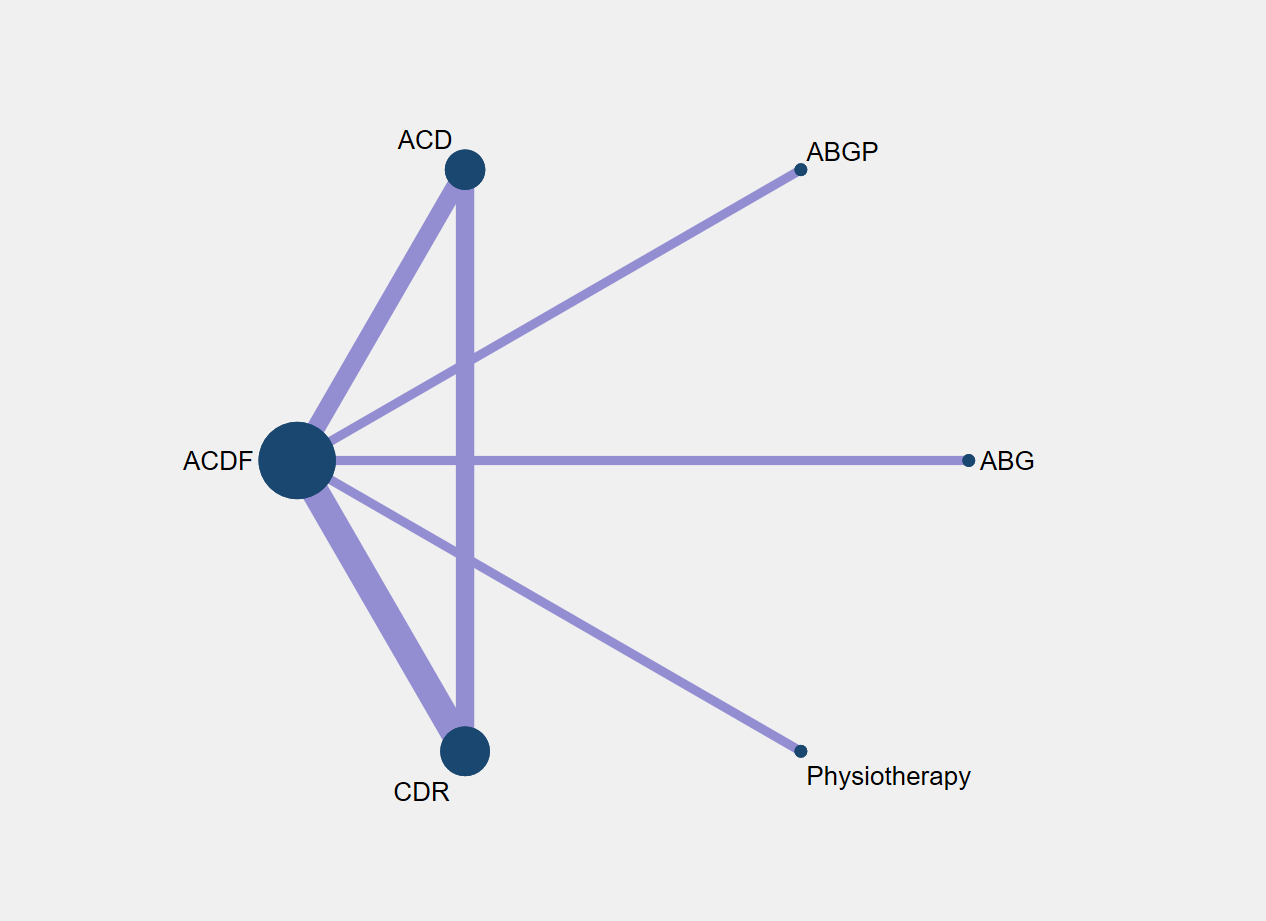
 Each circular node represents a type of treatment. The circle size is proportional to the total number of patients. The width of lines is proportional to the number of studies performing head-to-head comparisons in the same study. ABG: Anterior cervical discectomy with autologous bone graft, ABGP: Anterior cervical discectomy with allograft bone graft plus plating, ACD: Anterior cervical discectomy, ACDF: Anterior cervical discectomy and fusion, CDR: Cervical disc replacement.

**Supplementary Figure 39.** **ACD compared with ACDF on neck disability index (NDI).**
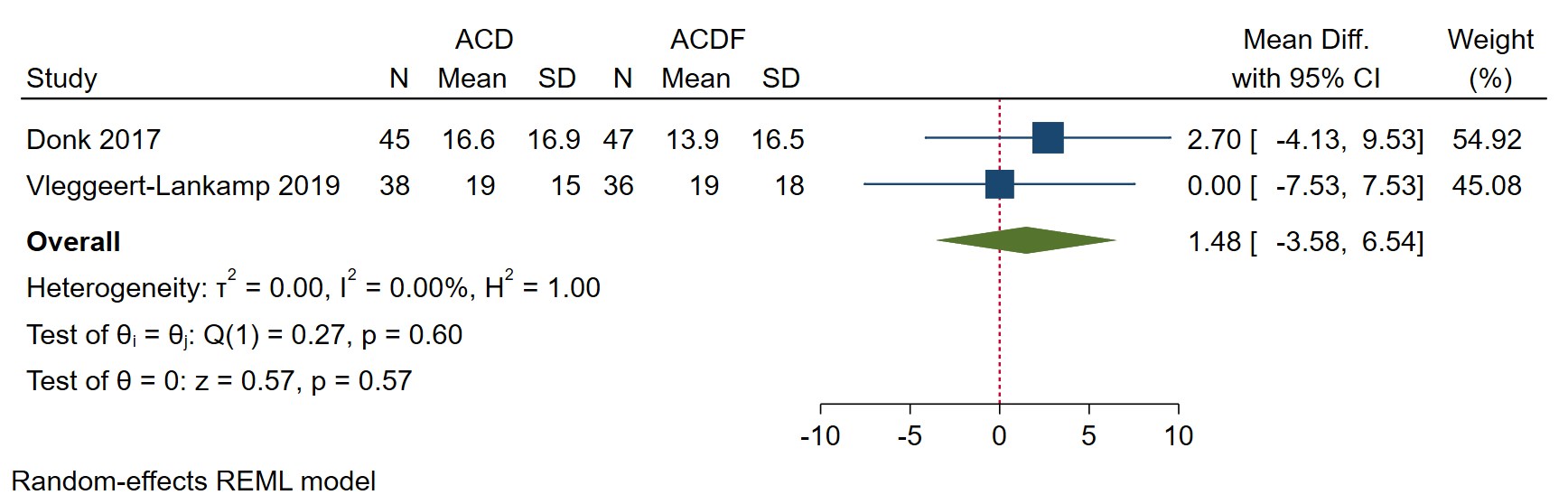


**Supplementary Figure 40.** **ACD compared with CDR on neck disability index (NDI).**
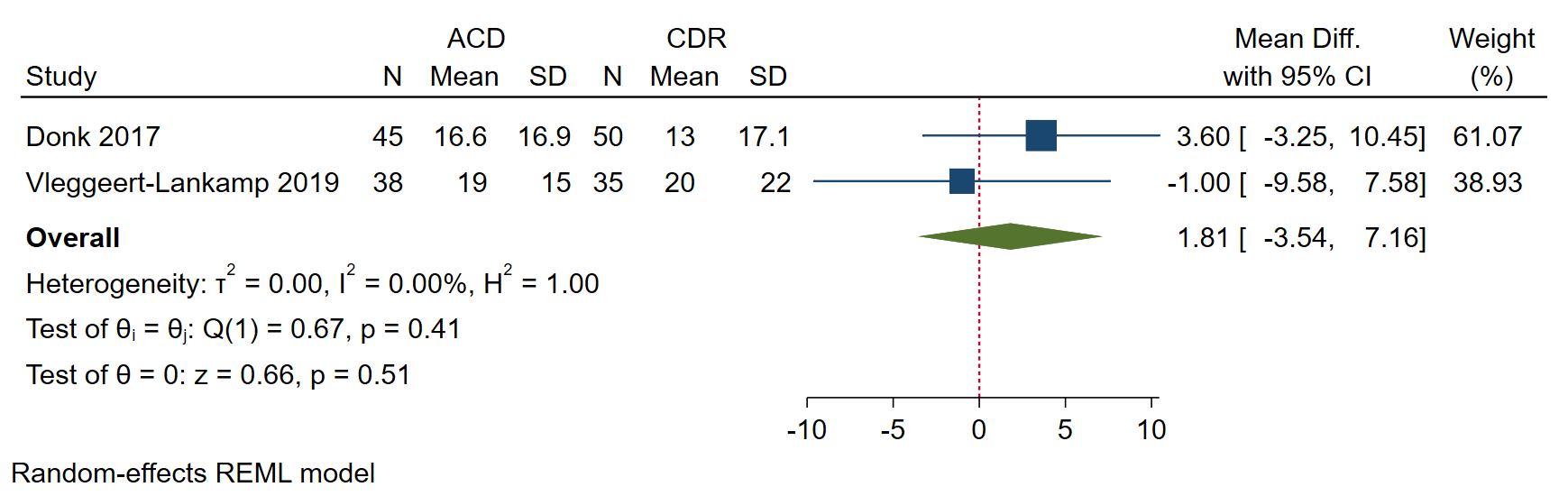


**Supplementary Figure 41.** **ACDF compared with CDR on neck disability index (NDI).**
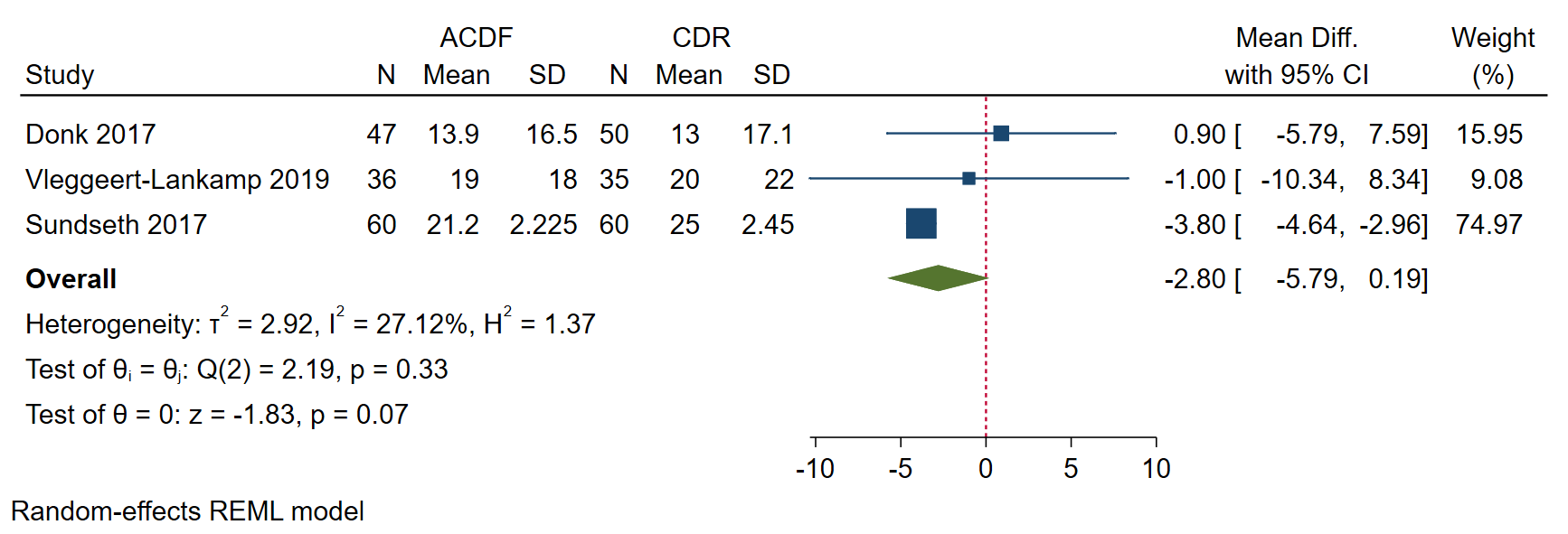


**Supplementary Figure 42.** **Forest plots depicting the direct and indirect results of neck disability index (NDI) and surgery time of head-to-head comparisons.**
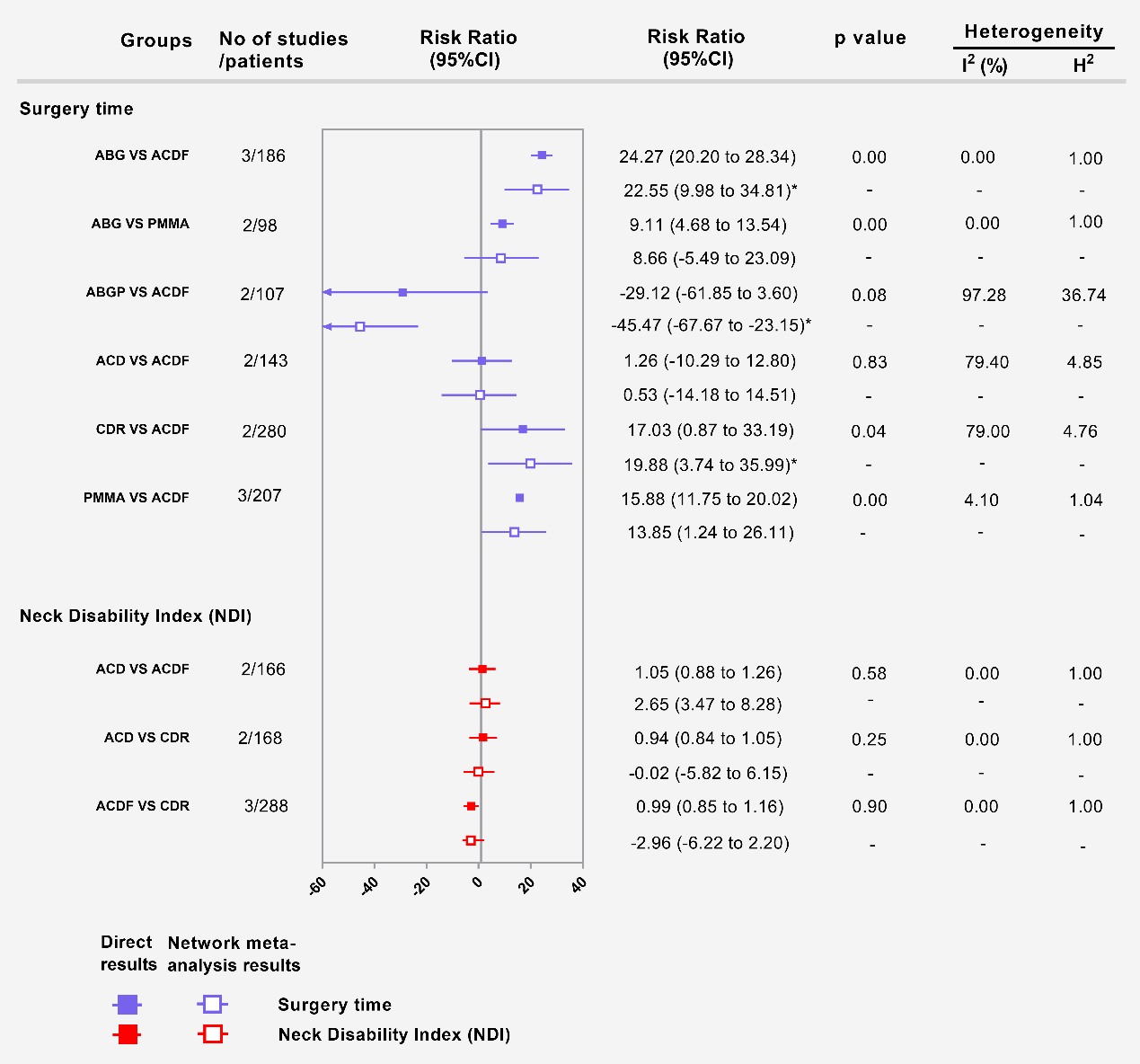
 ABG: Anterior cervical discectomy with autologous bone graft, ABGP: Anterior cervical discectomy with allograft bone graft plus plating, ACD: Anterior cervical discectomy, ACDF: Anterior cervical discectomy and fusion, CDR: Cervical disc replacement, PMMA: Anterior cervical discectomy with polymethylmethacrylate. *Values in brackets are 95% CI.

**Supplementary Figure 43.** **Network plots of comparison-based network meta-analyses on surgery time.**
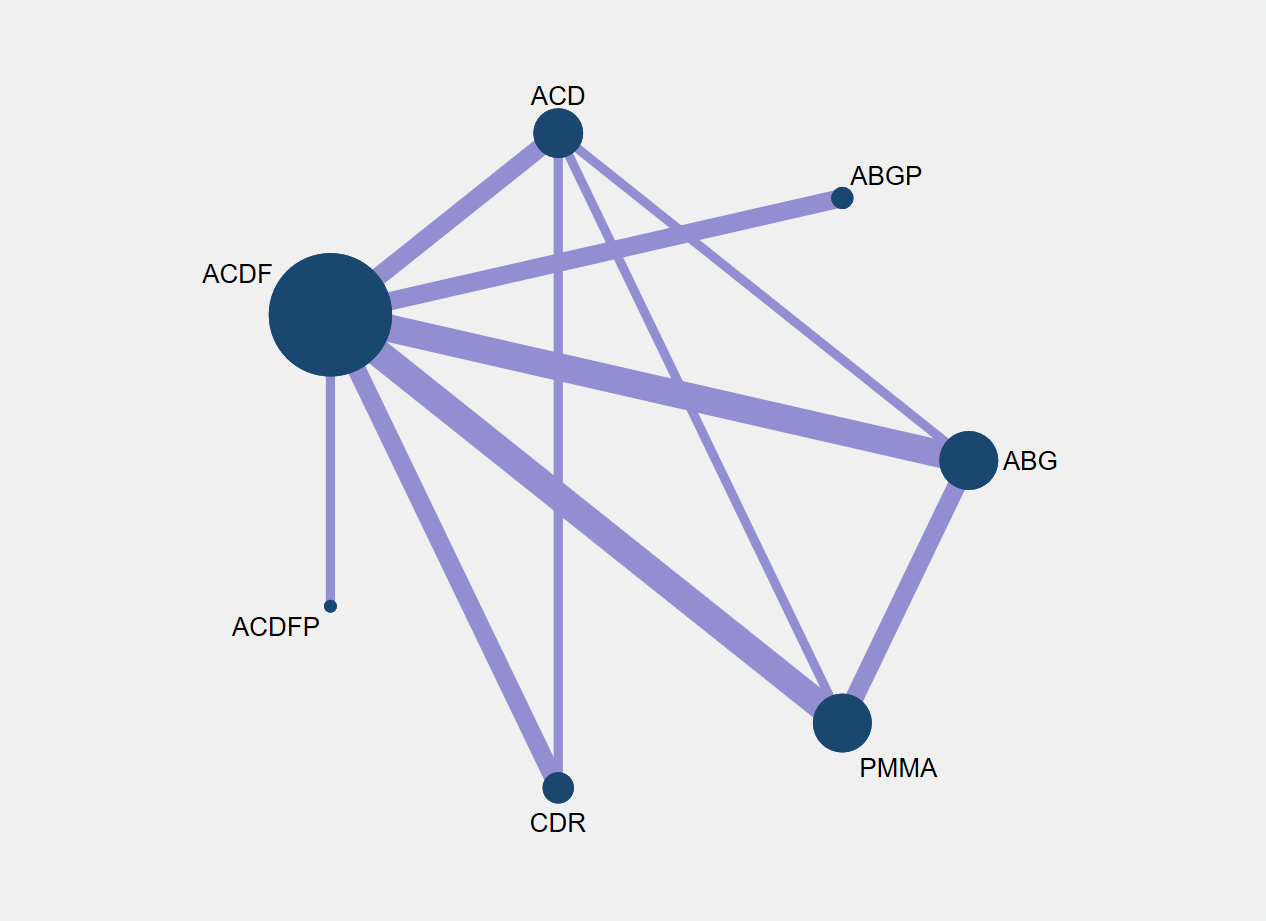
 Each circular node represents a type of treatment. The circle size is proportional to the total number of patients. The width of lines is proportional to the number of studies performing head-to-head comparisons in the same study. ABG: Anterior cervical discectomy with autologous bone graft, ABGP: Anterior cervical discectomy with allograft bone graft plus plating, ACD: Anterior cervical discectomy, ACDF: Anterior cervical discectomy and fusion, ACDFP: Anterior cervical discectomy with fusion and additional plating, CDR: Cervical disc replacement, PMMA: Anterior cervical discectomy with polymethylmethacrylate.

**Supplementary Figure 44.** **ABG compared with ACDF on surgery time.**
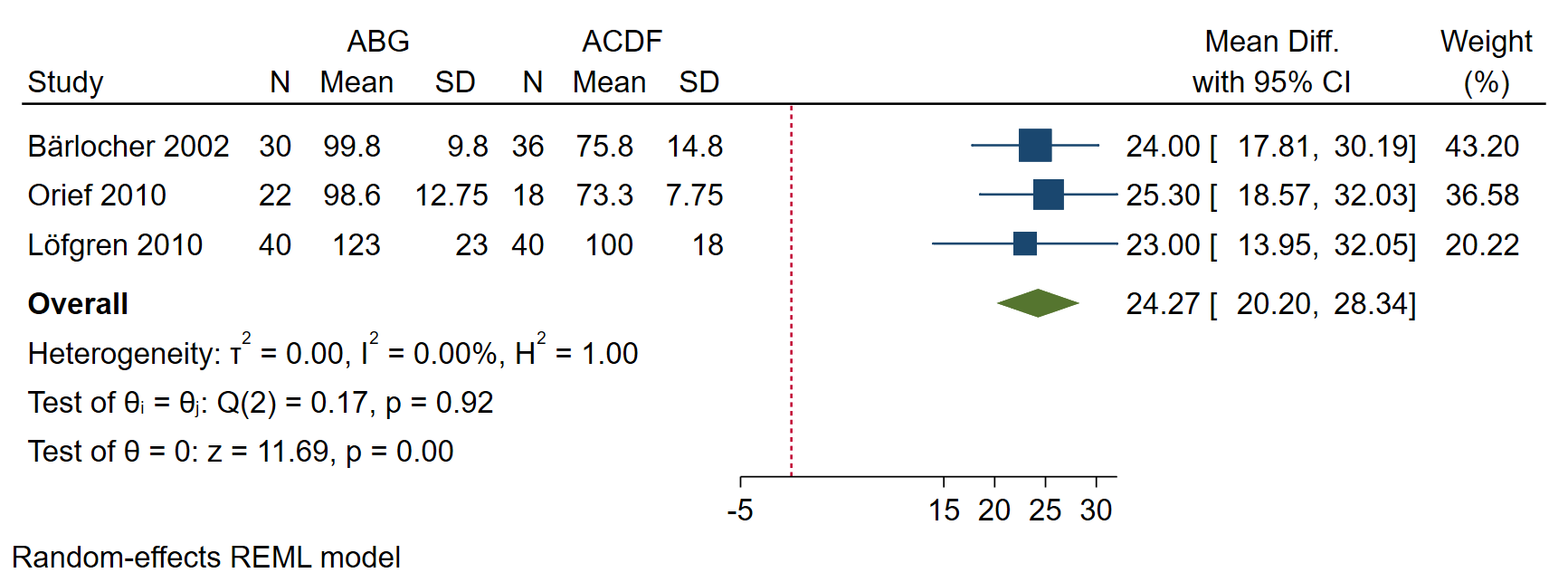


**Supplementary Figure 45.** **ABG compared with PMMA on surgery time.**
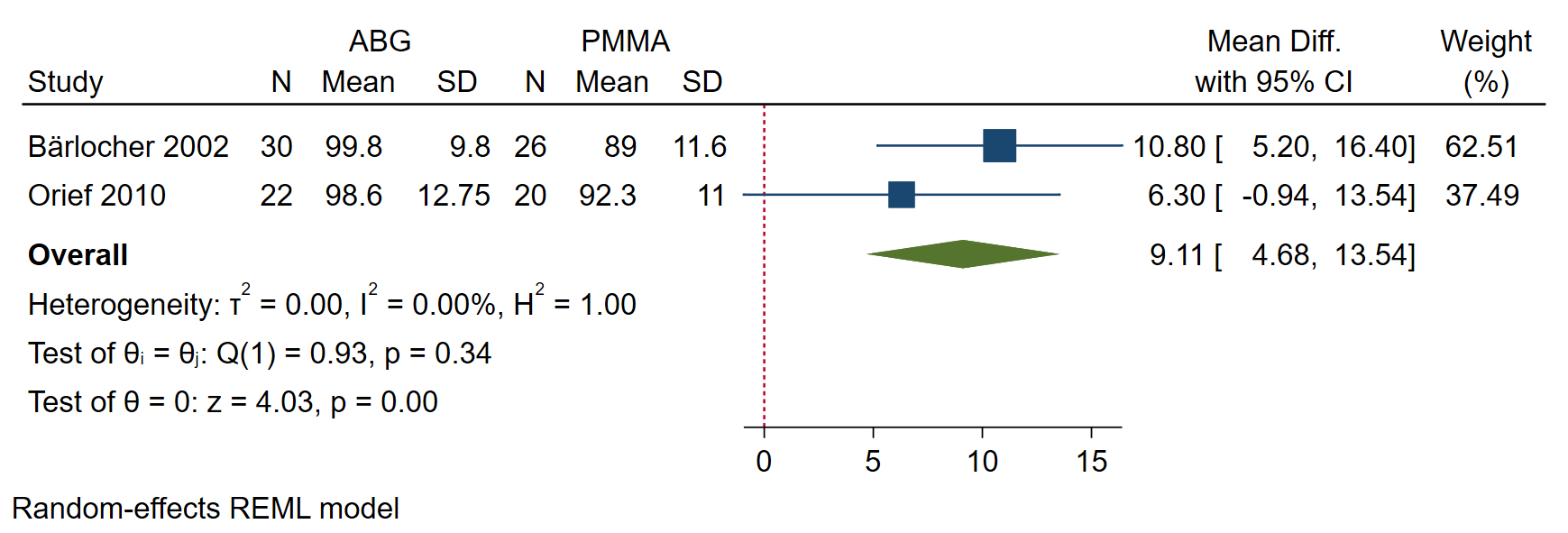


**Supplementary Figure 46.** **ABGP compared with ACDF on surgery time.**
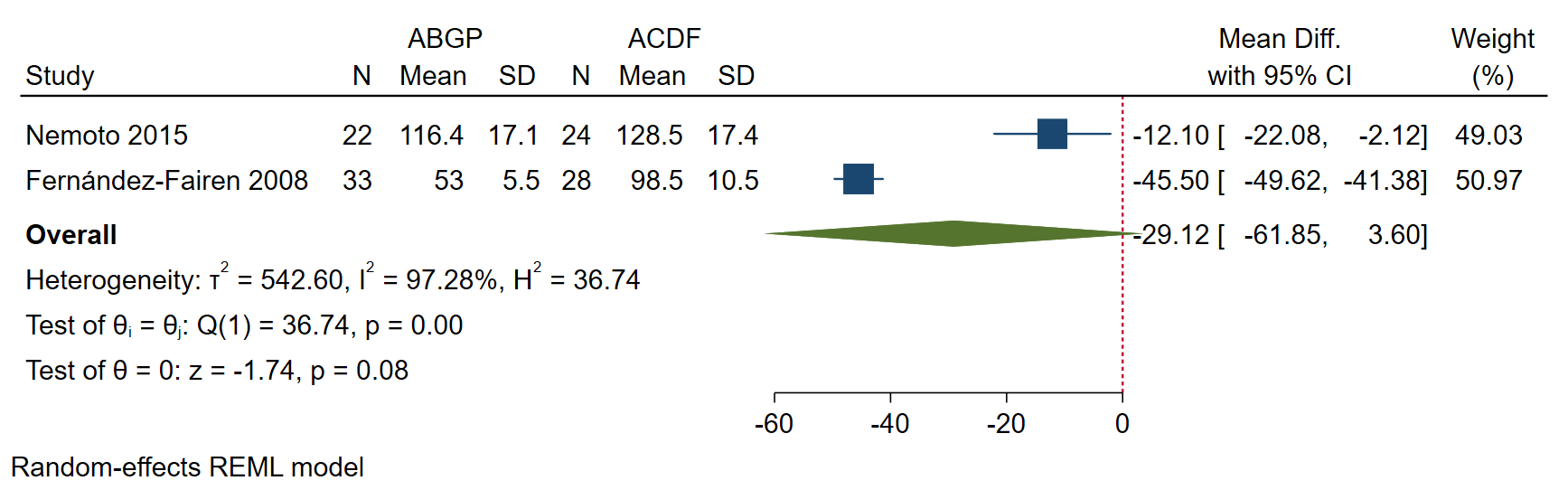


**Supplementary Figure 47.** **ACD compared with ACDF on surgery time.**
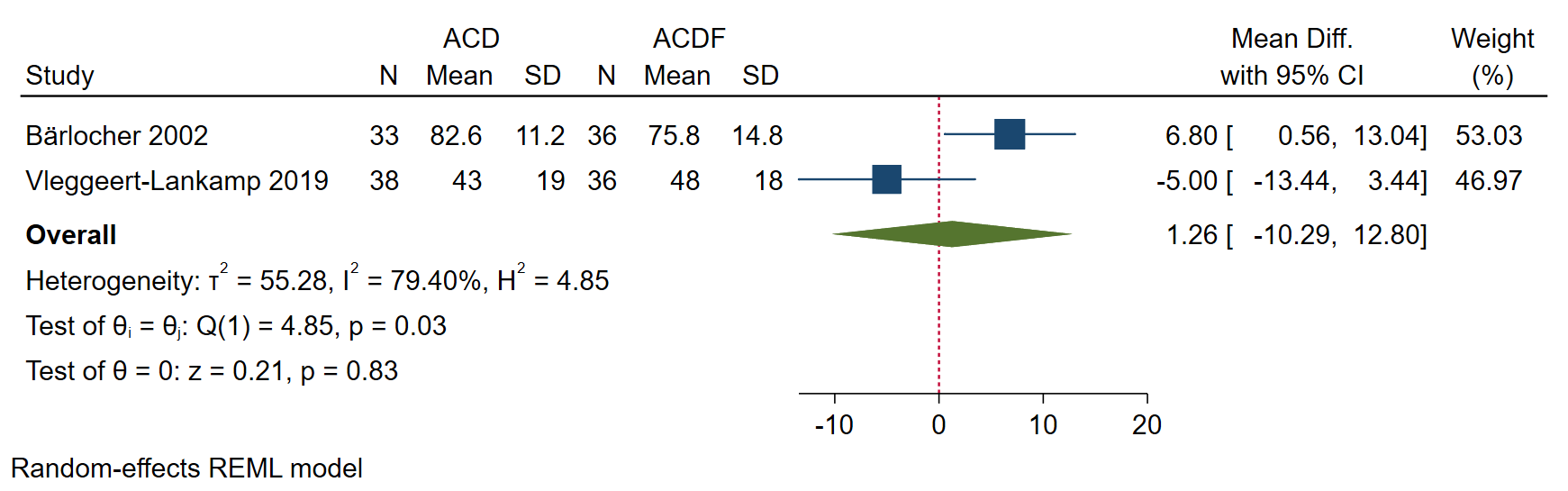


**Supplementary Figure 48.** **CDR compared with ACDF on surgery time.**
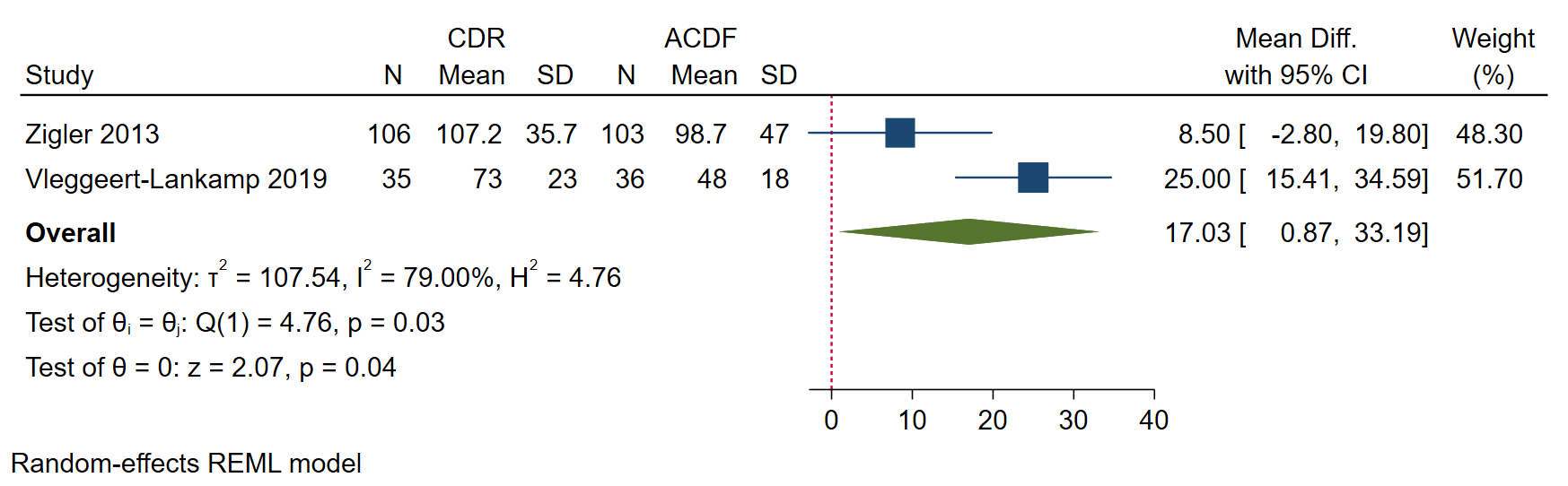


**Supplementary Figure 49.** **PMMA compared with ACDF on surgery time.**
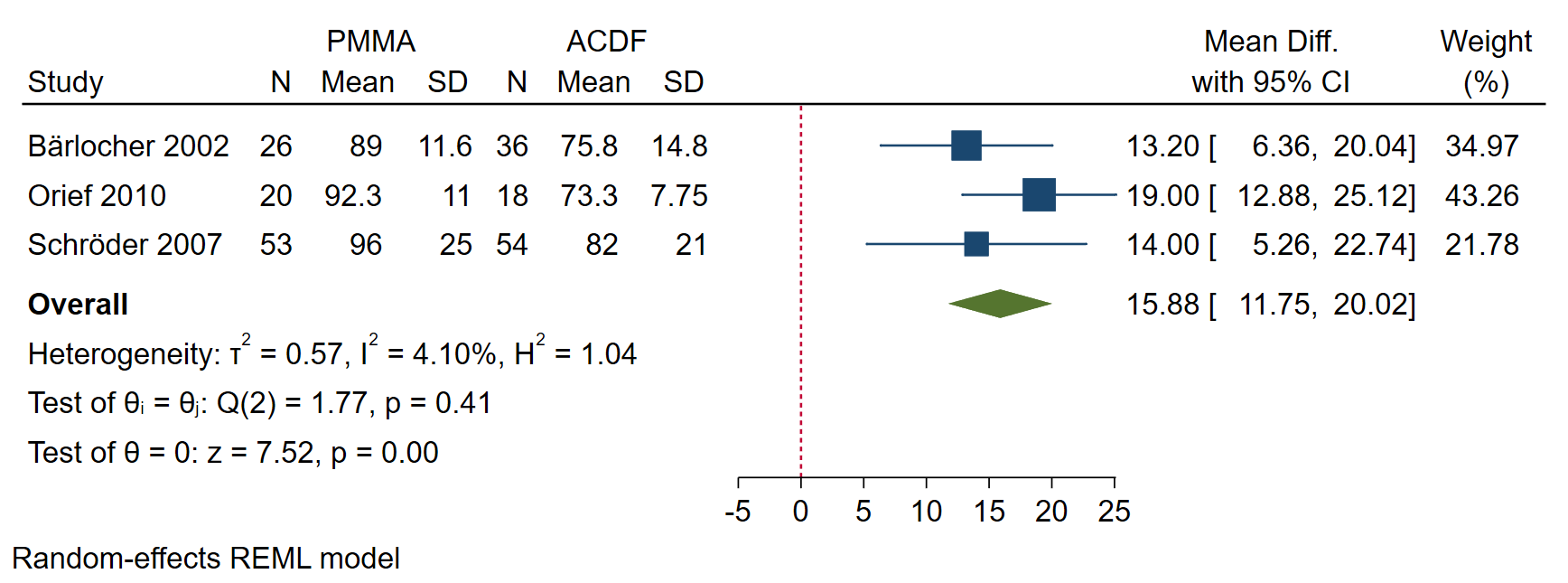


**Supplementary Table 1. Search strategy**

| # 1 | cervical vertebrae [MeSH Terms] |
| --- | --- |
| #2 | ((((((((((((diskectomy[MeSH Terms]) OR (foraminotomy[MeSH Terms])) OR (diskectomy[Title/Abstract])) OR (discectomy[Title/Abstract])) OR (foraminotomy[Title/Abstract])) OR (frykholm[Title/Abstract])) OR (fusion[Title/Abstract])) OR (disc replacement[Title/Abstract])) OR (disk replacement[Title/Abstract])) OR (disc arthroplast[Title/Abstract])) OR (disk arthroplast[Title/Abstract]) |
| #3 | (((((Randomized Controlled Trial [Publication Type]) OR (Controlled Clinical Trial [Publication Type])) OR (randomized [Title/Abstract])) OR (randomized [Title/Abstract])) OR (placebo [Title/Abstract])) OR (randomly [Title/Abstract]) |
| #4 | #1 AND #2 AND #3 |

**Supplementary Table 2. Inclusion/exclusion criteria of literature**

| **PICOS** | **Inclusion** | **Exclusion** |
| --- | --- | --- |
| P | Patients with pure cervical radiculopathy. | Patients with symptoms of myelopathy, radiculomyelopathy, or pure axial neck pain. |
| I | 1) Anterior or posterior surgical interventions;  2) No limit on sample size. | Did not differentiate between single-level and multilevel surgery were not eligible. |
| C | Anterior, posterior surgical interventions or conservative. | Did not differentiate between single-level and multilevel surgery were not eligible. |
| O | 1) Primary outcomes including the success rate of the intervention, Postoperative complication rate, postoperative reoperation rates, postoperative work status, disability, or pain scores.  2) Secondary outcome included surgery time, superficial wound infection, and all complications (including DVT, infection, revision, wound erythema/ecchymosis and so on). | Relevant outcomes were missing. |
| S | RCT irrespective of blinding or arm. | 1) Articles without peer-reviewed or unpublished;  2) Studies that were repeatedly published or had qualitative outcomes;  3) Quasi-experimental studies, crossover, and observational studies. |

**Supplementary Table 3. Risk of bias table.**

| 1 | Random sequence generation (selection bias) |
| --- | --- |
| 2 | Allocation concealment (selection bias) |
| 3 | Blinding of participants and personnel (performance bias) |
| 4 | Blinding of outcome assessment (detection bias) |
| 5 | Incomplete outcome data (attrition bias) |
| 6 | Selective reporting (reporting bias) |

**Supplementary Table 4. Characteristics of the included trials and participants.**

| **Number** | **Studies** | **Country** | **No. of**  **Centers** | **Total**  **Participants** | **Age† (yr)** | **Male/**  **Female** | **No. of**  **Arms** | **Control**  **Groups** | **Experimental**  **Groups** | **Follow-up (mo)** |
| --- | --- | --- | --- | --- | --- | --- | --- | --- | --- | --- |
| 1 | Savolainen 1998 | Finland | 1 | 91 | 47.8 | 63/28 | 3 | ACD: Anterior cervical discectomy | ABG: Anterior cervical discectomy with autologous bone graft  ABGP: Anterior cervical discectomy with autologous bone graft plus plating | 48 |
| 2 | Zoëga 2000 | Sweden | 1 | 27 | 41 (25-60) | 15/12 | 2 | ACDF: Anterior cervical discectomy and fusion (Solis cage (Stryker)) | ACDFP: Anterior cervical discectomy with fusion and additional plating | 24 |
| 3 | Wirth 2000 | USA | 1 | 72 | 43.5 (28-67) | 36/36 | 3 | ABG: Anterior cervical discectomy with autologous bone graft | PCF: Posterior cervical foraminotomy  ACD: Anterior cervical discectomy | 60 |
| 4 | Persson 2001 | Sweden | 1 | 81 | 47.5 ±7.9  (28-64) | 44/37 | 3 | ACDF: Anterior cervical discectomy with fusion (cow bone) | Physiotherapy  Cervical collar | 12 |
| 5 | Bärlocher 2002 | Switzerland | 1 | 125 | 50.5 ±11.4  (24-84) | 74/51 | 4 | ACDF: Anterior cervical discectomy with fusion（titanium cage） | PMMA: microdiscectomy followed by injection of polymethylmethacrylate  ACD: Anterior cervical discectomy  ABG: Anterior cervical discectomy with autologous bone graft | 12 |
| 6 | Nabhan 2006 | Germany | 1 | 33 | 45 ± 11 | 19/14 | 2 | ACDFP: ACDF and additional plating | CDR: Cervical disc replacement | 6 |
| 7 | Xie 2007 | Canada | Uncelar | 42 | 43 ± 8 (26-59) | 28/14 | 3 | ABG：Anterior cervical discectomy with autologous bone graft | ACD：Anterior cervical discectomy with autologous bone graft  ABGP：ABG with additional plating | 24 |
| 8 | Lind 2007 | Sweden | 1 | 24 | 42 (29-57) | 11/13 | 2 | ACDF: Anterior cervical discectomy with fusion (titanium cage) | ABG: Anterior cervical discectomy with autologous bone graft | 24 |
| 9 | Oktenoglu 2007 | Turkey | >1 | 20 | Median, 40.05 | 11/9 | 2 | ABGP: Anterior cervical discectomy with allograft bone graft plus plating | ACD: Anterior cervical microdiscectomy | 12 |
| 10 | Schröder 2007 | Germany | Unclear | 107 | 44.5 ± 8.5 | 62/45 | 2 | ACDF (titanium cage) | PMMA: Anterior cervical discectomy with polymethylmethacrylate | 24 |
| 11 | Fernändez-Fairen 2008 | Spain | 1 | 61 | 48.4 (22-65) | 22/39 | 2 | ABGP：Anterior cervical discectomy with allograft bone graft plus plating | ACDF: Anterior cervical discectomy with fusion (tantalum interbody implants) | 24 |
| 12 | Hauerberg 2008 | Denmark | 1 | 86 | Median, 45.5  (IQR, 10.5) | 43/43 | 2 | ACDF：Anterior cervical discectomy with fusion (titanium cage) | ACD: Anterior cervical discectomy | 24 |
| 13 | Ruetten 2008 | Germany | Unclear | 200 | 43 (27-62) | 68/132 | 2 | ACDF：Anterior cervical discectomy with fusion (polyetheretherketone (PEEK) cage) | PCF: posterior cervical foraminotomy) | 24 |
| 14 | Löfgren 2010 | Sweden | 1 | 80 | 49 (27-70) | 50/30 | 2 | ABG: Anterior cervical discectomy with autologous bone graft | ACDF: Anterior cervical discectomy with fusion (carbon fiber cage) | 24 |
| 15 | Orief 2010 | Egypt | 1 | 60 | 46.7 (28-68) | 34/26 | 2 | ABG: Anterior cervical discectomy with autologous bone graft | PMMA: Anterior cervical discectomy with polymethylmethacrylate  ACDF: Anterior cervical discectomy with fusion (PEEK cage) | 6 |
| 16 | Ebrahim 2011 | Egypt | 1 | 30 | 44.4 (29-62) | 14/16 | 2 | ACF: anterior cervical foraminotomy | PCF：Posterior cervical foraminotomy | 24 |
| 17 | Engquist 2013 | Sweden | 1 | 63 | 46.5 ± 8.5 | 33/30 | 2 | ACDF: Anterior cervical discectomy with fusion (titanium cage) | Physiotherapy | 24 |
| 18 | Zigler 2013 2015 janssen | USA | 13 | 209 | 42.8 ±7.8 | 95/114 | 2 | ACDF: anterior cervical discectomy and fusion | CDR: Cervical disc replacement | 60 |
| 19 | Nemoto 2015 | Japan | 1 | 46 | 41.3 ± 7.1  (31-54) | 42/4 | 2 | ACDFP: Anterior cervical discectomy with fusion and additional plating (PEEK cage) | ACDF (Zero-P): ACDF with zeroprofile cage as intervertebral spacer | 24 |
| 20 | Li 2015 | China | 1 | 23** | 48.7 ± 7.1  (25-65) | 11/12 | 2 | ACDFP: Anterior cervical discectomy with fusion and additional plating | ACDF (Zero-P): ACDF with zeroprofile cage as intervertebral spacer | 12 |
| 21 | Donk 2017 | Netherlands | 1 | 142 | 44.9 ± 6.5 | 71/71 | 3 | ACDF: Anterior cervical discectomy and fusion | ACD: Anterior cervical discectomy  CDR: Cervical disc replacement | 60 |
| 22 | Sundseth 2017 | Norway | 5 | 136 | 44.1 ± 7 | 63/73 | 2 | ACDF: Anterior cervical discectomy and fusion | CDR: Cervical disc replacement | 24 |
| 23 | Vleggeert-Lankamp 2019 | Netherlands | 1 | 109 | 46.8 ± 8 | 51/58 | 2 | ACDF: Anterior cervical discectomy and fusion (PEEK cage) | ACD: Anterior cervical discectomy  CDR: Cervical disc replacement | 24 |

**Supplementary Table 5. Node splitting analyses on postoperative success rates.**

| **Name** | **Direct Effect** | **Indirect Effect** | **Overall** | **P-Value** |
| --- | --- | --- | --- | --- |
| ABG, ACD | -0.36 (-2.70, 1.98) | -0.30 (-2.87, 2.16) | -0.43 (-2.04, 1.10) | 0.95 |
| ABG, PMMA | -0.22 (-2.05, 1.57) | 1.50 (-0.71, 4.15) | 0.38 (-1.22, 1.77) | 0.17 |
| ACD, PCF | 18.52 (0.26, 58.21) | 1.72 (-0.76, 4.64) | 1.95 (-0.30, 4.79) | 0.11 |
| ACD, PMMA | 0.84 (-1.52, 3.44) | 0.81 (-1.65, 2.85) | 0.82 (-0.92, 2.39) | 0.97 |

**Supplementary Table 6. Rank possibility of postoperative success rates.**

| **Interventions** | **Rank 1** | **Rank 2** | **Rank 3** | **Rank 4** | **Rank 5** | **Rank 6** | **Rank 7** | **Rank 8** | **Rank 9** | **Rank 10** |
| --- | --- | --- | --- | --- | --- | --- | --- | --- | --- | --- |
| ABG | 0.00 | 0.01 | 0.02 | 0.04 | 0.10 | 0.14 | 0.21 | 0.22 | 0.16 | 0.07 |
| ABGP | 0.04 | 0.07 | 0.10 | 0.09 | 0.09 | 0.10 | 0.11 | 0.12 | 0.13 | 0.14 |
| ACD | 0.00 | 0.00 | 0.01 | 0.02 | 0.03 | 0.06 | 0.11 | 0.19 | 0.29 | 0.29 |
| ACDF | 0.01 | 0.06 | 0.17 | 0.30 | 0.26 | 0.15 | 0.05 | 0.01 | 0.00 | 0.00 |
| ACDFP | 0.21 | 0.18 | 0.16 | 0.09 | 0.07 | 0.07 | 0.06 | 0.05 | 0.05 | 0.05 |
| ACF | 0.38 | 0.15 | 0.08 | 0.05 | 0.04 | 0.04 | 0.04 | 0.04 | 0.05 | 0.13 |
| CDR | 0.07 | 0.10 | 0.13 | 0.12 | 0.10 | 0.11 | 0.11 | 0.10 | 0.08 | 0.07 |
| PCF | 0.24 | 0.33 | 0.16 | 0.08 | 0.06 | 0.04 | 0.03 | 0.02 | 0.02 | 0.01 |
| PMMA | 0.02 | 0.05 | 0.08 | 0.13 | 0.15 | 0.17 | 0.16 | 0.12 | 0.07 | 0.03 |
| Physiotherapy | 0.03 | 0.05 | 0.08 | 0.07 | 0.08 | 0.10 | 0.11 | 0.12 | 0.14 | 0.21 |

**Supplementary Table 7. Node splitting analyses on postoperative complication rates.**

| **Name** | **Direct Effect** | **Indirect Effect** | **Overall** | **P-Value** |
| --- | --- | --- | --- | --- |
| ABG, ABGP | -0.47 (-2.99, 2.05) | 0.93 (-3.09, 5.42) | -0.07 (-2.36, 2.29) | 0.53 |
| ABG, ACD | -2.95 (-5.13, -1.04) | -2.51 (-5.14, -0.02) | -2.75 (-4.66, -0.95) | 0.76 |
| ABG, ACDF | -1.97 (-4.32, 0.23) | -2.80 (-5.45, -0.31) | -2.36 (-4.18, -0.68) | 0.60 |
| ABG, PMMA | -1.68 (-5.83, 2.28) | -2.11 (-6.31, 1.99) | -1.91 (-4.67, 0.70) | 0.87 |
| ABGP, ACD | -2.91 (-5.66, -0.27) | -1.88 (-4.88, 1.03) | -2.68 (-5.06, -0.47) | 0.56 |
| ABGP, ACDF | -3.21 (-7.81, 0.85) | -1.93 (-4.97, 0.83) | -2.28 (-4.71, -0.05) | 0.60 |
| ACD, ACDF | 0.16 (-1.83, 2.11) | 0.53 (-1.76, 2.81) | 0.39 (-1.18, 1.98) | 0.78 |
| ACD, CDR | -0.93 (-4.86, 2.90) | -0.33 (-3.90, 3.20) | -0.57 (-3.09, 1.94) | 0.80 |
| ACD, PCF | 0.68 (-3.17, 4.54) | 0.81 (-3.38, 4.97) | 0.77 (-1.83, 3.39) | 0.96 |
| ACD, PMMA | 0.48 (-3.86, 4.67) | 0.64 (-3.41, 4.75) | 0.84 (-1.77, 3.49) | 0.95 |

**Supplementary Table 8. Rank possibility of postoperative complication rates.**

| **Interventions** | **Rank 1** | **Rank 2** | **Rank 3** | **Rank 4** | **Rank 5** | **Rank 6** | **Rank 7** | **Rank 8** | **Rank 9** |
| --- | --- | --- | --- | --- | --- | --- | --- | --- | --- |
| ABG | 0.37 | 0.41 | 0.15 | 0.05 | 0.01 | 0.00 | 0.00 | 0.00 | 0.00 |
| ABGP | 0.38 | 0.35 | 0.16 | 0.07 | 0.03 | 0.01 | 0.00 | 0.00 | 0.00 |
| ACD | 0.00 | 0.00 | 0.03 | 0.07 | 0.14 | 0.20 | 0.26 | 0.22 | 0.09 |
| ACDF | 0.00 | 0.00 | 0.04 | 0.15 | 0.26 | 0.28 | 0.19 | 0.07 | 0.01 |
| ACF | 0.08 | 0.06 | 0.10 | 0.11 | 0.09 | 0.07 | 0.09 | 0.14 | 0.25 |
| CDR | 0.00 | 0.01 | 0.03 | 0.05 | 0.07 | 0.10 | 0.16 | 0.27 | 0.32 |
| PCF | 0.02 | 0.05 | 0.16 | 0.22 | 0.19 | 0.14 | 0.12 | 0.09 | 0.03 |
| PMMA | 0.03 | 0.05 | 0.20 | 0.20 | 0.15 | 0.12 | 0.10 | 0.09 | 0.05 |
| Physiotherapy | 0.12 | 0.07 | 0.13 | 0.09 | 0.07 | 0.07 | 0.08 | 0.12 | 0.25 |

**Supplementary Table 9. Node splitting analyses on postoperative reoperation rates.**

| **Name** | **Direct Effect** | **Indirect Effect** | **Overall** | **P-Value** |
| --- | --- | --- | --- | --- |
| ABG, ABGP | -0.32 (-2.88, 1.99) | -0.32 (-4.49, 3.97) | -0.35 (-2.44, 1.60) | 0.99 |
| ABG, ACD | 0.42 (-1.14, 2.12) | -1.03 (-3.16, 0.92) | -0.09 (-1.49, 1.32) | 0.20 |
| ABG, ACDF | -0.91 (-3.02, 0.93) | 0.68 (-1.24, 2.61) | -0.12 (-1.53, 1.31) | 0.19 |
| ABGP, ACD | 0.58 (-1.66, 2.93) | -0.25 (-2.71, 2.12) | 0.27 (-1.57, 2.27) | 0.53 |
| ABGP, ACDF | 0.19 (-3.78, 4.24) | 0.31 (-2.01, 2.83) | 0.25 (-1.67, 2.26) | 0.95 |
| ACD, ACDF | 0.32 (-0.58, 1.16) | -1.03 (-2.32, 1.24) | -0.01 (-0.94, 0.87) | 0.20 |
| ACD, CDR | -0.51 (-1.87, 0.77) | 1.81 (0.46, 3.25) | 0.43 (-0.89, 1.67) | 0.02 |
| ACD, PCF | 0.61 (-1.52, 2.99) | 0.27 (-2.10, 2.70) | 0.36 (-1.19, 1.92) | 0.81 |

**Supplementary Table 10. Rank possibility of postoperative reoperation rates.**

| **Interventions** | **Rank 1** | **Rank 2** | **Rank 3** | **Rank 4** | **Rank 5** | **Rank 6** | **Rank 7** | **Rank 8** | **Rank 9** |
| --- | --- | --- | --- | --- | --- | --- | --- | --- | --- |
| ABG | 0.02 | 0.10 | 0.14 | 0.14 | 0.13 | 0.13 | 0.16 | 0.14 | 0.05 |
| ABGP | 0.02 | 0.08 | 0.09 | 0.09 | 0.08 | 0.09 | 0.12 | 0.23 | 0.21 |
| ACD | 0.00 | 0.03 | 0.07 | 0.14 | 0.19 | 0.21 | 0.19 | 0.13 | 0.03 |
| ACDF | 0.00 | 0.01 | 0.05 | 0.13 | 0.21 | 0.25 | 0.21 | 0.11 | 0.03 |
| ACF | 0.15 | 0.23 | 0.10 | 0.06 | 0.05 | 0.05 | 0.07 | 0.11 | 0.17 |
| CDR | 0.03 | 0.20 | 0.22 | 0.19 | 0.14 | 0.09 | 0.07 | 0.05 | 0.02 |
| PCF | 0.03 | 0.15 | 0.22 | 0.16 | 0.13 | 0.11 | 0.10 | 0.07 | 0.03 |
| PMMA | 0.03 | 0.08 | 0.06 | 0.06 | 0.05 | 0.06 | 0.07 | 0.14 | 0.45 |
| Physiotherapy | 0.72 | 0.13 | 0.05 | 0.03 | 0.02 | 0.01 | 0.01 | 0.01 | 0.01 |

**Supplementary Table 11. Node splitting analyses on postoperative work status.**

| **Name** | **Direct Effect** | **Indirect Effect** | **Overall** | **P-Value** |
| --- | --- | --- | --- | --- |
| ABG, ACDF | 2.32 (-0.30, 5.82) | 0.06 (-2.15, 2.41) | 0.91 (-0.94, 3.02) | 0.16 |
| ACD, ACDF | 0.14 (-1.14, 1.75) | -0.26 (-26.12, 26.39) | 0.16 (-1.10, 1.76) | 0.98 |

**Supplementary Table 12. Rank possibility of postoperative work status.**

| **Interventions** | **Rank 1** | **Rank 2** | **Rank 3** | **Rank 4** | **Rank 5** | **Rank 6** | **Rank 7** | **Rank 8** | **Rank 9** |
| --- | --- | --- | --- | --- | --- | --- | --- | --- | --- |
| ABG | 0.00 | 0.02 | 0.03 | 0.05 | 0.08 | 0.11 | 0.17 | 0.26 | 0.28 |
| ABGP | 0.10 | 0.13 | 0.11 | 0.10 | 0.09 | 0.09 | 0.11 | 0.13 | 0.15 |
| ACD | 0.02 | 0.05 | 0.10 | 0.17 | 0.21 | 0.20 | 0.15 | 0.08 | 0.02 |
| ACDF | 0.02 | 0.08 | 0.16 | 0.21 | 0.21 | 0.17 | 0.11 | 0.04 | 0.01 |
| ACDFP | 0.20 | 0.18 | 0.14 | 0.10 | 0.08 | 0.08 | 0.07 | 0.07 | 0.07 |
| ACF | 0.19 | 0.14 | 0.10 | 0.08 | 0.06 | 0.07 | 0.08 | 0.10 | 0.18 |
| CDR | 0.04 | 0.06 | 0.07 | 0.09 | 0.10 | 0.12 | 0.15 | 0.17 | 0.20 |
| PCF | 0.09 | 0.17 | 0.16 | 0.11 | 0.10 | 0.10 | 0.11 | 0.11 | 0.06 |
| PMMA | 0.34 | 0.17 | 0.12 | 0.09 | 0.07 | 0.07 | 0.06 | 0.05 | 0.03 |

**Supplementary Table 13. Node splitting analyses on scores for arm pain.**

| **Name** | **Direct Effect** | **Indirect Effect** | **Overall** | **P-Value** |
| --- | --- | --- | --- | --- |
| ACD, ACDF | -0.10 (-2.20, 2.04) | -0.82 (-4.20, 2.42) | -0.28 (-1.67, 1.24) | 0.51 |

**Supplementary Table 14. Rank possibility of scores for arm pain.**

| **Interventions** | **Rank 1** | **Rank 2** | **Rank 3** | **Rank 4** | **Rank 5** | **Rank 6** | **Rank 7** |
| --- | --- | --- | --- | --- | --- | --- | --- |
| ABG | 0.41 | 0.17 | 0.09 | 0.08 | 0.10 | 0.10 | 0.05 |
| ABGP | 0.08 | 0.04 | 0.03 | 0.03 | 0.04 | 0.08 | 0.71 |
| ACD | 0.18 | 0.30 | 0.24 | 0.13 | 0.09 | 0.05 | 0.01 |
| ACDF | 0.02 | 0.11 | 0.22 | 0.33 | 0.26 | 0.05 | 0.00 |
| ACDFP | 0.03 | 0.04 | 0.05 | 0.08 | 0.17 | 0.47 | 0.15 |
| CDR | 0.05 | 0.13 | 0.23 | 0.23 | 0.21 | 0.13 | 0.03 |
| Physiotherapy | 0.23 | 0.21 | 0.13 | 0.12 | 0.13 | 0.13 | 0.05 |

**Supplementary Table 15. Node splitting analyses on scores for neck pain.**

| **Name** | **Direct Effect** | **Indirect Effect** | **Overall** | **P-Value** |
| --- | --- | --- | --- | --- |
| ACDF, ACDFP | 0.23 (-0.74, 1.21) | -1.04 (-2.54, 0.46) | -0.17 (-1.16, 0.85) | 0.11 |
| ACDF, CDR | -0.25 (-1.10, 0.58) | 1.02 (-0.41, 2.44) | 0.15 (-0.87, 0.97) | 0.09 |
| ACDFP, CDR | 0.79 (-0.35, 1.96) | -0.46 (-1.77, 0.75) | 0.33 (-0.89, 1.30) | 0.10 |

**Supplementary Table 16. Rank possibility of scores for neck pain.**

| **Drug** | **Rank 1** | **Rank 2** | **Rank 3** | **Rank 4** | **Rank 5** | **Rank 6** | **Rank 7** |
| --- | --- | --- | --- | --- | --- | --- | --- |
| ABG | 0.21 | 0.07 | 0.03 | 0.02 | 0.03 | 0.18 | 0.46 |
| ABGP | 0.05 | 0.06 | 0.04 | 0.03 | 0.04 | 0.33 | 0.45 |
| ACD | 0.06 | 0.21 | 0.21 | 0.17 | 0.19 | 0.12 | 0.03 |
| ACDF | 0.01 | 0.12 | 0.24 | 0.33 | 0.22 | 0.07 | 0.01 |
| ACDFP | 0.02 | 0.08 | 0.13 | 0.19 | 0.33 | 0.21 | 0.04 |
| CDR | 0.05 | 0.24 | 0.29 | 0.22 | 0.13 | 0.06 | 0.01 |
| Physiotherapy | 0.59 | 0.23 | 0.06 | 0.04 | 0.04 | 0.03 | 0.01 |

**Supplementary Table 17. Rank possibility of scores for neck disability index (NDI).**

| **Drug** | **Rank 1** | **Rank 2** | **Rank 3** | **Rank 4** | **Rank 5** | **Rank 6** |
| --- | --- | --- | --- | --- | --- | --- |
| ABG | 0.01 | 0.01 | 0.02 | 0.05 | 0.20 | 0.71 |
| ABGP | 0.03 | 0.06 | 0.09 | 0.17 | 0.47 | 0.19 |
| ACD | 0.22 | 0.33 | 0.27 | 0.10 | 0.06 | 0.02 |
| ACDF | 0.01 | 0.05 | 0.25 | 0.53 | 0.15 | 0.01 |
| CDR | 0.17 | 0.43 | 0.28 | 0.08 | 0.03 | 0.01 |
| Physiotherapy | 0.57 | 0.11 | 0.10 | 0.07 | 0.09 | 0.07 |

**Supplementary Table 18. Node splitting analyses on surgery time.**

| **Name** | **Direct Effect** | **Indirect Effect** | **Overall** | **P-Value** |
| --- | --- | --- | --- | --- |
| ABG, ACD | -17.08 (-37.92, 3.59) | -32.48 (-56.81, -8.74) | -22.07 (-39.21, -5.85) | 0.26 |
| ABG, PMMA | -8.56 (-26.10, 9.54) | -8.69 (-37.78, 21.06) | -8.66 (-23.09, 5.49) | 1.00 |
| ACD, CDR | 28.89 (7.82, 51.41) | 6.01 (-20.36, 32.97) | 19.36 (0.91, 38.28) | 0.14 |
| ACD, PMMA | 6.94 (-19.12, 31.97) | 16.51 (-6.65, 39.96) | 13.35 (-3.09, 30.42) | 0.49 |

**Supplementary Table 19. Rank possibility of scores for surgery time.**

| **Drug** | **Rank 1** | **Rank 2** | **Rank 3** | **Rank 4** | **Rank 5** | **Rank 6** | **Rank 7** |
| --- | --- | --- | --- | --- | --- | --- | --- |
| ABG | 0.58 | 0.35 | 0.06 | 0.01 | 0.00 | 0.00 | 0.00 |
| ABGP | 0.00 | 0.00 | 0.00 | 0.00 | 0.00 | 0.00 | 0.99 |
| ACD | 0.00 | 0.01 | 0.04 | 0.37 | 0.25 | 0.32 | 0.00 |
| ACDF | 0.00 | 0.00 | 0.01 | 0.25 | 0.50 | 0.23 | 0.00 |
| ACDFP | 0.01 | 0.02 | 0.06 | 0.26 | 0.22 | 0.44 | 0.00 |
| CDR | 0.36 | 0.40 | 0.20 | 0.03 | 0.01 | 0.00 | 0.00 |
| PMMA | 0.05 | 0.23 | 0.63 | 0.07 | 0.02 | 0.00 | 0.00 |
